# Frustrated smectic liquid crystal elastomers as multifunctional mechanical metamaterials

**DOI:** 10.1038/s41467-026-76776-x

**Published:** 2026-09-11

**Authors:** Aidan Street, Stuart R. Berrow, Zhenming Wang, Emily J. Cooper, Johan Mattsson, Helen F. Gleeson

**Affiliations:** School of Physics and Astronomy, University of Leeds, Leeds, UK

**Keywords:** Liquid crystals, Mechanical properties, Polymers, Polymers

## Abstract

Auxetics are materials that show a negative Poisson’s ratio, i.e. thickening when under tensile strain. The discovery of nematic liquid crystal elastomers (LCEs) as molecular auxetic materials was a paradigm-shift in the design of mechanical metamaterials as they are tuneable, transparent, scalable, non-porous and facile to fabricate. Here, we report that smectic LCEs extend known synthetic molecular auxetics to a second family. The LCEs are acrylate-based, lightly crosslinked ( ~ 8 mol%) and polymerised in the nematic phase of a precursor mixture. The resulting smectic-A LCEs exhibit correlation lengths of a few molecular lengths, suggesting layer frustration, which we confirm occurs via the mechanism predicted by de Gennes in 1975. Three of the LCEs described exhibit negative Poisson’s ratios at strains above ~40%, thereby marking the observation of auxetic behaviour in smectic LCEs. Shear oscillatory measurements on planar and homeotropic LCEs demonstrate highly anisotropic moduli, enabling elucidation of highly anisotropic adhesive properties, with differences as large as 20x suggested between geometries. Room temperature tack measurements reveal a 31% difference in adhesion between geometries. Furthermore, we find that in the homeotropic case, shear moduli are comparable in both nematic and smectic A LCEs, validating theoretical expectations for such systems.

## Abstract (web summary)

Auxetic liquid crystal elastomers are tunable, scalable, and transparent materials. Here the authors report acrylate based liquid crystal elastomers, which exhibit smectic phases with low correlation lengths, and thus evidence of layer frustration. The elastomers show auxetic behaviour and anisotropic adhesive properties.

## Introduction

Metamaterials are exciting systems, with properties such as negative refractive indices or coefficients of thermal expansion that are not normally found in nature. Auxetics are a class of mechanical metamaterials that exhibit negative rather than positive Poisson’s ratios, meaning they expand under tensile strain rather than contracting, offering mechanical advantages that include resistance to impact, delamination and tear^1,2^. So-called re-entrant auxetics, which have been known for several decades, are porous, honeycomb structures in which the auxetic response comes from rotation or deformation of a unit cell wherein volume is not conserved^2^. Large-scale re-entrant auxetic structures have been successfully included in some sportsgear (e.g. running shoes^3^ and tennis rackets^4^) but there is no clear route to mass-production of honeycomb structures at sub-micron scales.

**Fig. 1:**
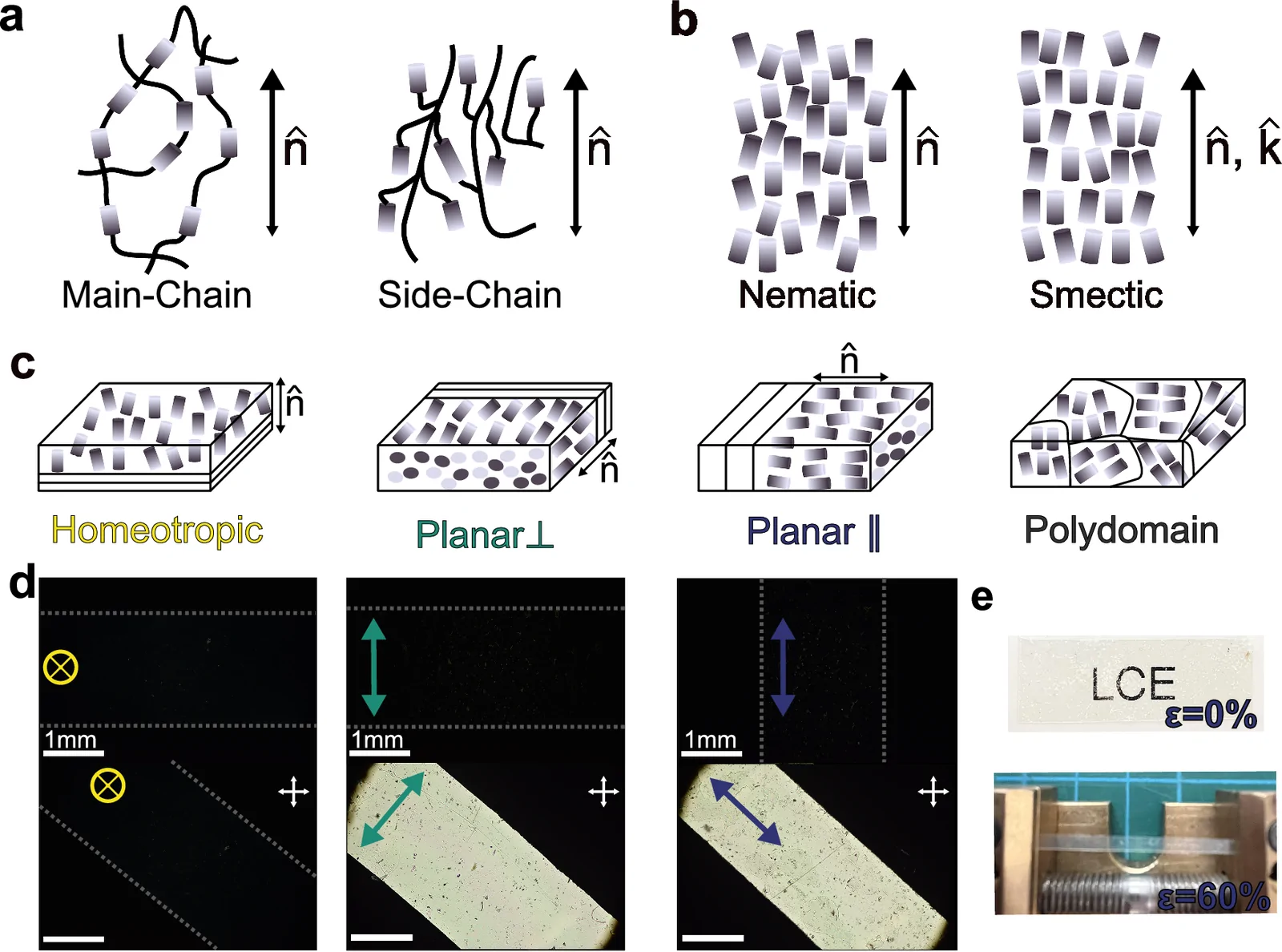
Order and Alignment of LCEs. **a** Schematic showing main-chain and side-chain liquid crystal elastomers. **b** The molecular order in the nematic and smectic-A liquid crystal phases, also indicating the director, $\hat{n}$, and the smectic layer normal, k̂. **c** Macroscopic geometries typically found in liquid crystal elastomers indicating polydomain, homeotropic and planar geometries. In dynamic mechanical analysis experiments it is appropriate to distinguish between shear perpendicular and parallel to the director in planar geometries, as shown. **d** Polarised microscopy images of LCE3 films in homeotropic and planar geometries demonstrate the high-quality monodomain alignment (a polydomain film would be opaque). The scale bar is 1 mm and the orange, green and red arrows show the director direction. The white arrows indicate the crossed polarizers. **e** (Upper) unstrained LCE3 placed above text reading ‘LCE’ to show the transparency, (lower) the same LCE in a strain rig at $\varepsilon=60\%$; the visibility of the rig and background showcases the transparency under strain.

The discovery of a family of side chain nematic liquid crystal elastomers (LCEs) (Fig. 1a, b^5^) as the first synthetic *molecular* auxetics was a breakthrough that transformed the range of possible applications for auxetic materials. In addition to the known benefits of impact and delamination resistance seen by all auxetics, LCEs are the only known transparent^6^ auxetics, are scalable from the nanometre regime upwards^7^, are highly tunable^8^, and offer the potential of facile large-scale manufacture^9^. These additional features make auxetic LCEs exciting options for applications where porous auxetics may be unsuitable. For example, the transparency of the auxetic LCEs (94% at 589 nm)^6^ makes them exciting candidates in applications where optical clarity is imperative, something that traditional porous auxetics cannot achieve. The auxetic response in nematic LCEs is mediated by a biaxial deformation of the director under strain, accompanied by an out-of-plane molecular rotation which causes a negative Poisson’s ratio above a material-dependent threshold strain (usually above ~50%)^10,11^. Intriguingly, it was recently observed that some smectic LCEs also exhibit biaxiality under strain^8,12^, but as smectics usually fail at strains above 20–30%, which is well below typical auxetic strain-thresholds, an auxetic response was expected to be unachievable. This paper shows that frustrated smectic-A (SmA) systems, which exhibit a subtle combination of smectic and nematic features, can be strained well beyond 30%. Auxetic behaviour is observed in the frustrated SmA LCEs, introducing a second class of materials to known molecular auxetic systems. The bulk mechanical properties of SmA LCEs differ substantially from nematic variants^13–15^ so this discovery represents a broadening of the suite of physical properties in LCEs that might be desirable for applications.

Liquid crystal polymers combine the order of liquid crystals with properties of polymeric systems, which when loosely cross-linked to yield LCEs, includes rubber-like behaviour typical of elastomers. Such materials have a long history, with the noteworthy discovery of the main chain high-tensile fibre, Kevlar^®^, in the 1960s followed by significant research activity over the intervening decades that saw the first LCEs synthesised by Finkelmann in the 1980s^15–17^. The field of LCEs strictly stems from de Gennes, who first predicted the extraordinary properties of an elastomer with mesogenic order^18^. A multitude of applications have been suggested to exploit the mechanical properties of LCEs, including; impact dampers^19–21^; auxetics^5,6,10^; soft-robotic actuators^22–24^; and smart adhesives^25–28^. However, a further prediction by de Gennes, suggested that templating macromolecules in various liquid crystal phases would modify the resultant mechanical properties^16^. Specifically, he suggested that polymerising a nematic low molar mass (precursor) system to form a smectic LCE would result in a frustrated smectic layer structure, with mechanical properties that would be distinct from nematic and conventional smectic LCEs. Such an approach is often known as ‘templating’ and it offers an alternative, largely unexplored, approach to modifying the bulk properties of LCEs^12,29^. We follow this route as an approach to produce SmA LCEs with negative Poisson’s ratios.

Berrow et al. reported the synthesis of frustrated and conventional SmA structures in acrylate LCEs when studying how changing the condensed phase in which polymerisation is conducted effects the properties of the resulting LCEs^12^. The frustrated nature of the SmA phase was identified by a low correlation length which was found to be less than ~5 molecular lengths in those frustrated SmA systems and more than ~10 in the ‘true’ smectics. It was clear from that work that the two different smectic systems were readily achieved by polymerising in different phases; a nematic precursor tended to form a frustrated SmA LCE, whereas polymerising in a SmA phase produced LCEs with large correlation lengths, in agreement with de Gennes’ prediction^12^. The mechanical properties of these smectic LCEs were explored up to strains of ~30%; it was shown that the frustrated materials had layer compression moduli that were a factor of ~5 lower than in the conventional SmA LCEs and that they deformed biaxially at low strain. We have now seen that some of the frustrated SmA LCEs can be strained beyond 30% and here we present the large-strain behaviour of these materials, demonstrating that a room temperature auxetic response is realised in some systems where large strains can be achieved. We also present the anisotropic shear moduli of two frustrated SmA materials, one of which is auxetic, allowing us to consider multiple applications of frustrated SmA LCEs. The shear moduli specifically allow one to consider the potential of LCEs as transparent, anisotropic pressure sensitive adhesives, which is another timely application area being considered for nematic LCEs, but which has not yet been reported for smectic systems^25,26,28,30^.

## Results

### Evolution of smectic order

**Fig. 2:**
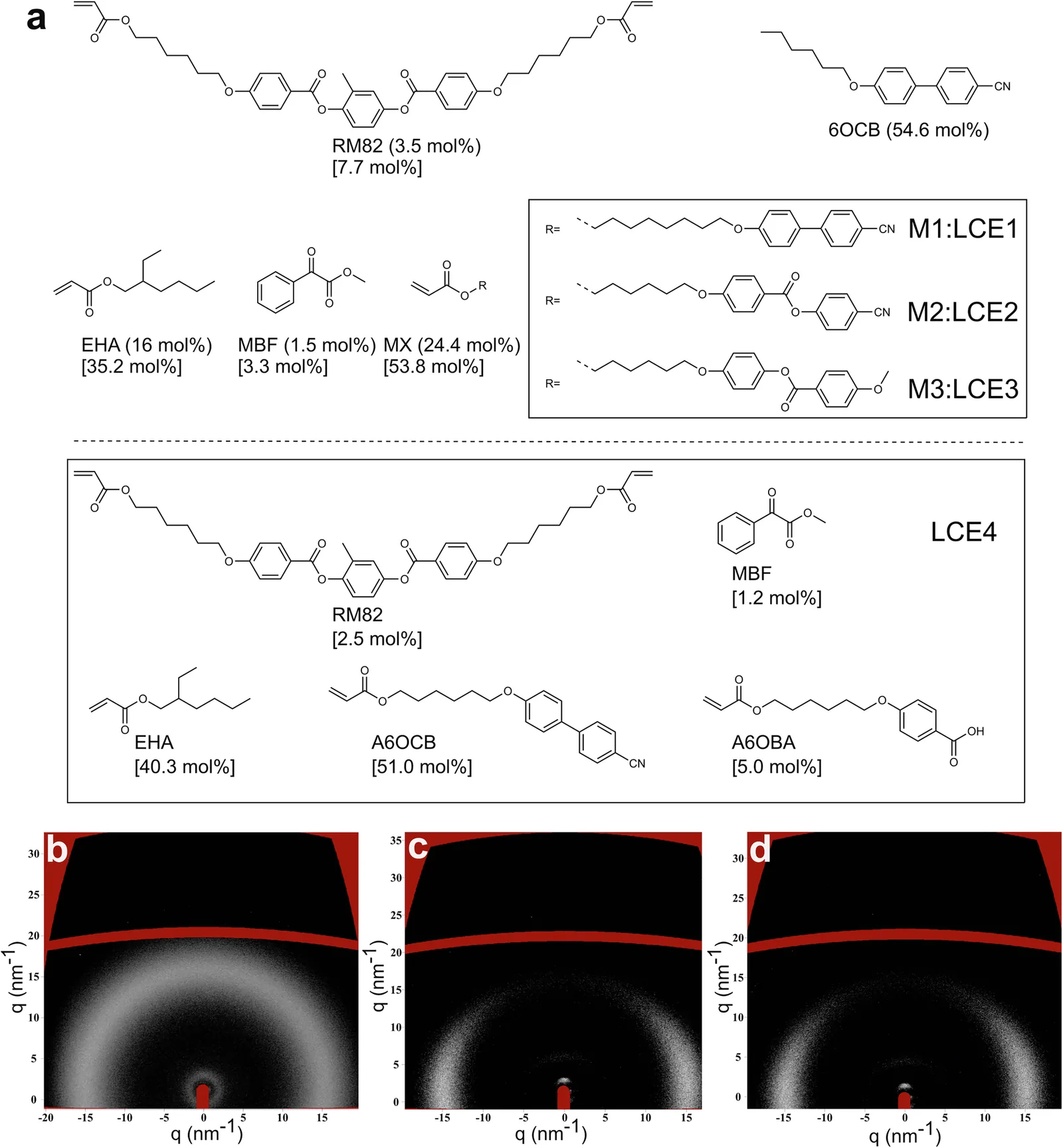
LCE compositions. **a** The composition of the LCE mixtures used for the synthesis of the smectic LCEs in this work. The quantities in curved brackets () represent the molar percentage of each component used in the LCE precursor mixtures, and the quantities in square brackets [] represent the molar percentage of the component present in the final LCEs. X-ray scattering data for LCE1 for (**b**) the precursor mixture, showing a diffuse SAXS peak (**c**) a planar LCE1 sample after curing but before the removal of 6OCB, and **d** LCE1 after removal of the 6OCB.

In three of the materials considered in this work (LCE1, LCE2, and LCE3), the films were polymerised in a precursor mixture which includes ~50% of a non-reactive liquid crystal 6OCB, included to ensure a room temperature nematic phase. We have previously shown that the final LCE films are SmA, despite the polymerisation being conducted with a nematic precursor mixture^8,12^. These LCEs exhibit a frustrated SmA phase, characterised by relatively short smectic correlation lengths (typically of the order of two to four smectic layers) and they exhibit some nematic-like mechanical properties^12^. The formation of the frustrated SmA phase is explained as a consequence of the distribution of cross-links in the final elastomer being influenced by the nematic order in the precursor mixture, as suggested by de Gennes.^16^ The unreacted 6OCB is washed out after curing and an important question is at what stage the SmA phase forms? Is it during the cure, which would make the LCE consistent with the de Gennes theory, or does it result from the washout step, i.e. the SmA phase is simply the result of a change in composition in the LCE + 6OCB phase diagram? The answers to these questions are provided through X-ray scattering studies at each step of the LCE synthesis (Fig. 2 b-d).

We examined the evolution of the smectic ordering in LCE1 through X-ray scattering studies of the unaligned nematic precursor mixture held in a capillary, and aligned samples of the LCE before and after the wash-out step (Fig. 2b–d). The correlation lengths, SmA layer spacing and order parameters of each LCE, elucidated from X-ray scattering, are given in Table 1. The precursor mixture displays an isotropic-like X-ray scattering pattern as there is no preferred director orientation in the unaligned nematic phase in the capillary. However, the nematic nature of this mixture has been confirmed by differential scanning calorimetry and polarised light optical microscopy (Figs. S1 and S3)^8^. The formation of the SmA phase upon curing and prior to the removal of 6OCB, Fig. 2c, is evidenced by a sharp Bragg (001) reflection in the small angle region at q = 1.83 nm^−1^, together with the orthogonality of the small and wide angle scattering signal (the assignment of the scattering features is indicated in Fig. S6). Upon removal of the 6OCB via solvent washing, the SmA phase is retained. The orientational order parameter and smectic correlation length in the swollen (unwashed) system (0.62 and 205 Å respectively) are both larger than those observed in the final LCE (0.56 and 161 Å respectively). The higher degree of orientational order in the unwashed sample can be attributed to its higher mesogenic content which is ~80% compared with ~60% in the final LCE. Interestingly, the layer spacing is considerably smaller in the unwashed LCE (34 Å compared with 41 Å), a value that we note is comparable to the 32 Å layer spacing reported for the interdigitated structure of 8OCB^31^. This thereby suggests an increased degree of interdigitation of the mesogens in the unwashed sample than is present after the removal of 6OCB.

**Table 1:** The correlation length, smectic layer spacing and order parameters of the LCEs for each formulation, elucidated from X-ray scattering measurements

Material	Correlation length (Å/N^o^ of layers)	SmA layer spacing (Å)	Order parameter
LCE1 unwashed film	205/6	34	0.62 ( ± 0.03)
LCE1	161/5	41	0.56 ( ± 0.03)
LCE2	100/2	45	0.61 ( ± 0.03)
LCE3	119/3	41	0.61 ( ± 0.03)
LCE4	189/4	48	0.59 ( ± 0.03)

In the case of LCE1, the evolution of these parameters throughout the fabrication process (i.e. unwashed film vs final LCE) is given.Values in brackets () show the error in the reported values of order parameter.

### Mechanical properties of frustrated smectic LCEs

Nematic molecular auxetic LCEs have been reported to exhibit negative Poisson’s ratios above a material- and temperature-dependent threshold strain which is typically ~50%^5,8,10,32^. The auxetic response has been shown to occur via a biaxial deformation that occurs upon straining perpendicular to the liquid crystal director and the circumstances under which a biaxial rather than uniaxial director reorientation occurs have recently been presented^10,11^. Intriguingly, some SmA LCEs have also been seen to deform biaxially though those studies were limited to low strain regimes as similar smectic LCEs had been seen to fail consistently at strains in excess of 30%^8,12^. Since that work, we have observed that samples of some frustrated SmA LCEs are able to withstand strains far in excess of 30% prior to failure and, excitingly, they also show an auxetic response.

**Fig. 3:**
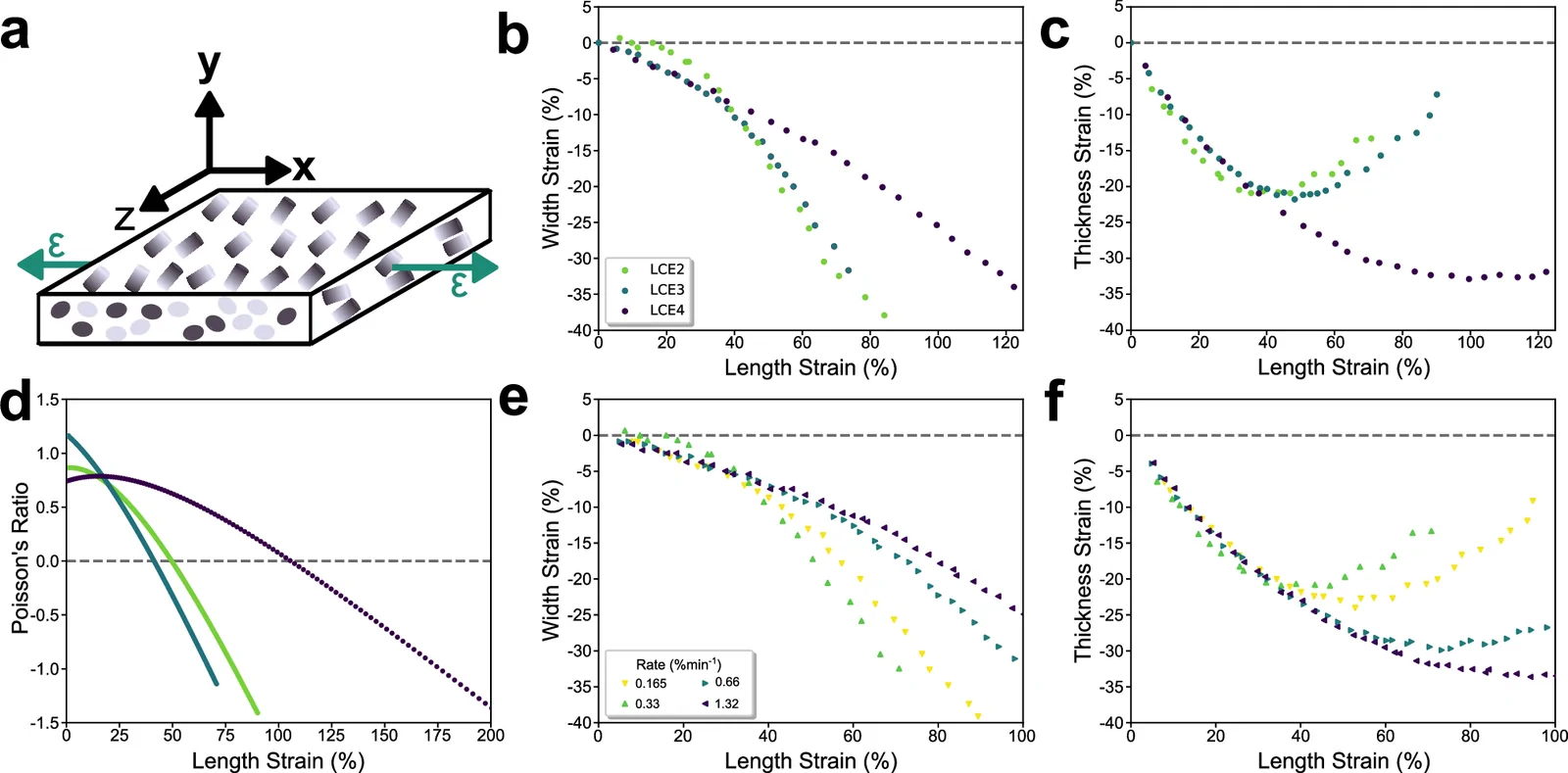
Auextic behaviour in LCEs. **a** A schematic representation of the experimental conditions employed in the analysis of auxeticity in the LCE samples, defining the naming convention of the x (length), y (thickness) and z (width) axes, where ε represents the applied strain and the rods depict the alignment of the mesogens in the LCE. **b**, **c** Show the effect of an applied length (x) strain on the sample dimensions in the width (z) and thickness (y) dimensions respectively for each sample when strained at a rate of 0.33%/min at room temperature. An increase in sample thickness occurs above threshold length strains of 41 ± 5%, 50 ± 5% and 105 ± 5% for LCE2, LCE3 and LCE4 respectively. LCE1 consistently failed at strains of ~30%. **d** Shows the instantaneous Poisson’s ratio in the y-axis (ν) deduced for each sample as a function of the applied x-strain. **e**, **f** Show the measured strain (in width and length respectively) for LCE2 strained at different rates: 0.165%/min (down triangles), 0.33%/min (upright triangles), 0.66%/min (right-facing triangles), and 1.32%/min (left-facing triangles). The auxetic thresholds become 53 ± 5%, 47 ± 5%, and 73 ± 5% for 0.5x, 1x, and 2x respectively, an auxetic threshold is not observed at a rate of 4x up to a strain of 100% although the initial strain behaviour is similar.

Figure 3 shows the effect of a strain applied perpendicular to the director (and layer normal) on the sample dimensions of LCE films of LCE2, LCE3, and LCE4 in the transverse axes. As the strain is applied perpendicular to the initial director, the sample width (*z*-axis in Fig. 3a and data in Fig. 3b), decreases throughout the entire strain range, for all the films. However, Fig. 3c which shows changes in the sample thickness (*y*-axis in Fig. 3a), demonstrate completely different behaviour which is reminiscent of the nematic LCEs. The sample thickness initially decreases for all systems but above a threshold value (50 ± 5%, 41 ± 5%, and 105 ± 5% for LCE2, LCE3, and LCE4, respectively) the sample thickness begins to increase, i.e. the Poisson’s ratios (Fig. 3d) become negative. These results therefore demonstrate that when sufficiently high strains can be achieved in some frustrated SmA side-chain LCEs, an auxetic response can be readily observed. It is noteworthy that the auxetic threshold values of LCE2 and LCE3 are lower than any previously reported for nematic LCEs (≥56%)^8,10,32^, showing an improved response of these systems.

For auxetic nematic LCEs, it has previously been observed that the auxetic threshold strain is dependent on several factors, most notably dynamics, for example the temperature or the rate at which strain is applied to the sample^8,33^. Given that the layered structure present in smectic LCEs adds more geometric considerations than in nematic LCEs, we were interested to undertake some initial studies to explore whether the strain rate similarly impacts the auxetic response in these smectic LCE systems. In these experiments, samples of LCE2 were examined at different strain rates, namely 0.165%/min, 0.33%/min, 0.66%/min and 1.32%/min, and the data are shown in Fig. 3e, f.

In nematic auxetic LCEs, it has been reported that as the strain rate is increased, the auxetic threshold of the nematic LCE increases, as faster strain rates can be thermorheologically related to lower temperatures^33^. The data reported in Fig. 3e, f suggest that in the frustrated smectic LCEs there is a similar strain-rate dependence. Taking the rate of 0.33%/min as the base strain rate as this is the rate used to compare samples of different composition (Fig. 3b–d), it is observed that as the strain rate is increased (doubled and quadrupled to 0.66%/min and 1.32%/min respectively), the auxetic threshold also increases. This increase is such that for the sample strained at 1.32%/min, no auxetic response is observed prior to sample failure. Interestingly, we find that if the strain rate is halved from the original 0.33%/min to 0.165%/min, the auxetic threshold remains comparable. One may expect a decrease in the auxetic threshold based on this change in strain rate, so this result is somewhat surprising. This finding may result from a constraint imposed by the layered structure present in the frustrated smectic sample, or it may simply be that the change in strain rate imposed was insufficient to substantially change the dynamics of the system. These initial findings suggest that a more complex relationship potentially exists between strain rate and auxetic threshold in these frustrated smectic systems than in the nematic case. A future, more detailed study considering factors such as strain rate and temperature will be conducted to deduce this relationship.

Furthermore, it has been previously reported that the auxetic response in nematic LCEs appears to be governed by proximity to the glass transition temperature (*T*_g_) of the sample, with a higher *T*_g_ leading to higher auxetic thresholds^8,33^. For the smectic auxetic LCEs reported here, there is no facile relationship between *T*_g_ and the auxetic threshold; LCE2, LCE3 and LCE4 have a *T*_g_ of 7 °C, 0 °C and 1 °C by differential scanning calorimetry respectively (Table S1), yet the auxetic thresholds follow the order LCE3 < LCE2 < LCE4, indicating a non-monotonic relationship. These results therefore suggest that the auxetic response in smectic LCEs may be influenced by more characteristics than those known to influence the auxetic response in nematic LCEs. Attempts to elucidate these characteristics will be the subject of future studies.

**Fig. 4:**
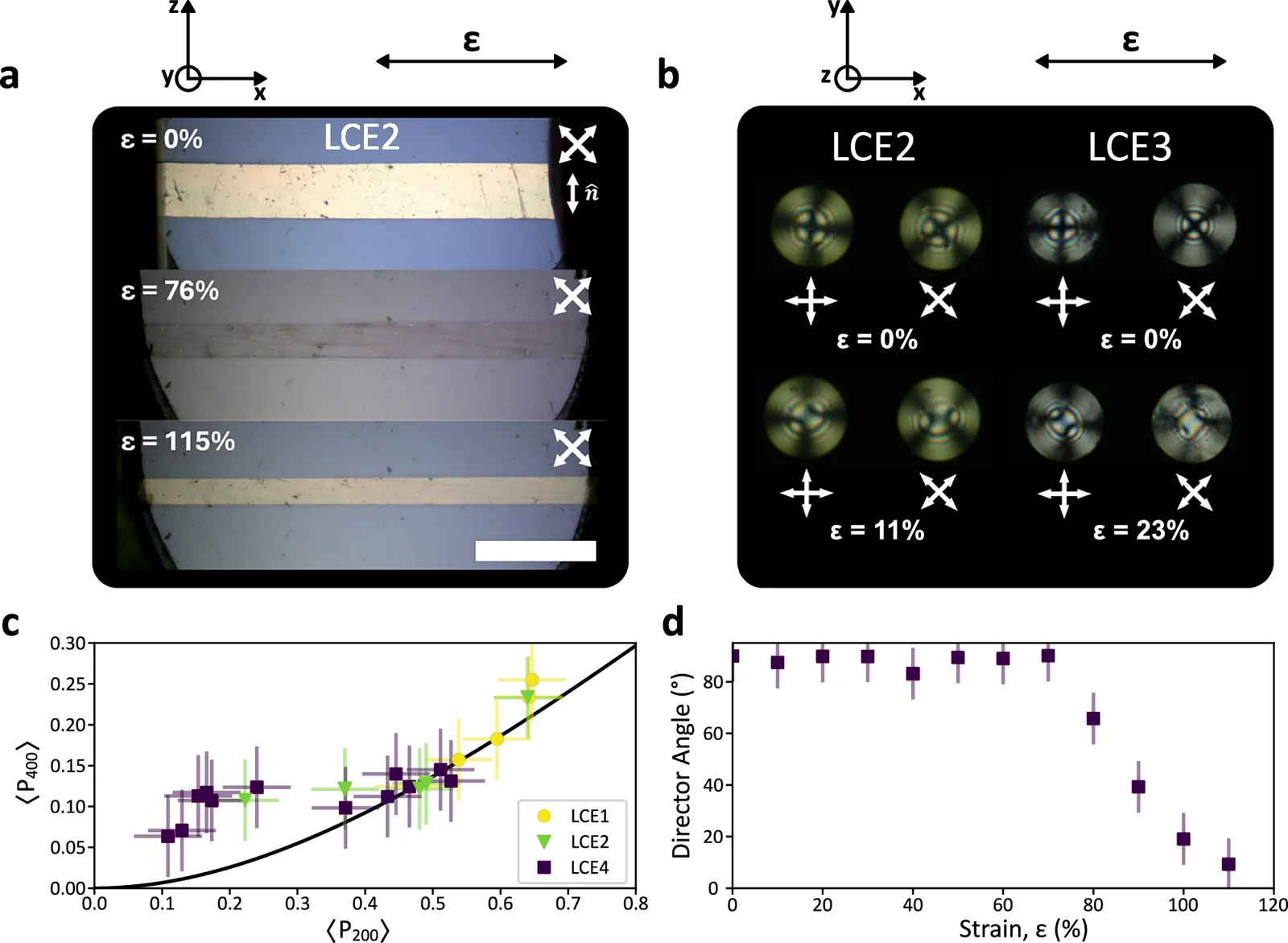
Emergence of Biaxiality in Auxetic LCEs. **a** A sample of planar LCE2 examined as a function of strain (ε) under crossed-polarisers, with the initial director ($\hat{{{{\boldsymbol{n}}}}}$) indicated by the arrow on the topmost image and the scale bar represents 0.5 cm. The transition from a bright state to a dark state, and then back to the bright state is clearly observed, consistent with behaviour observed for nematic auxetic LCE. **b** Conoscopic figures for homeotropic LCE2 and LCE3, showing a Maltese cross indicative of uniaxial order when unstrained and two melatopes when strained (ε ≠ 0%), indicative of the emergence of biaxial order. **c** The fourth-order uniaxial order parameter, <P_400_ > , as a function of the second-order uniaxial order parameter, <P_200_ > , for LCE1 (yellow circles), LCE2 (green inverted triangles), and LCE4 (purple squares) determined by polarised Raman spectroscopy. Both order parameters reduce in magnitude with increasing strain. A clear deviation from the Maier-Saupe theoretical prediction (black line) is seen as the LCE is strained, this is indicative of the emergence of biaxial order. **d** The apparent director angle as a function of strain in LCE4, an apparent rapid reorientation begins at 70% strain. Error bars are given by the uncertainty in the best-fit function.

The auxetic response in nematic LCEs is driven by a biaxial deformation, in which an increasing proportion of mesogens align in the transverse axis when strain is applied perpendicular to the initial director^10,11^. As frustrated smectic LCEs exhibit a layered structure on a molecular level, we were interested to examine if this same biaxial deformation existed in these materials, and hence was the cause of the auxetic response. A combination of polarised light optical microscopy, conoscopy, and polarised Raman spectroscopy was used to examine the emergence of biaxiality in these samples (Fig. 4).

When samples are visualised under crossed polarisers (Fig. 4a for LCE2), the samples initially show a uniform bright state. Upon the application of strain, this bright state persists until a critical strain (76%) at which point, the birefringence of the sample becomes zero and a uniform dark state is observed. Upon further strain, the sample begins to show birefringence colour again. These observations suggest a director reorientation process is occurring upon strain, and are entirely analogous to observations made for nematic auxetic LCEs^5^. The emergence of biaxiality in the samples is directly observed via conoscopic studies of homeotropically aligned samples. Prior to the application of strain, the samples show a Maltese cross in the conoscopic figure (Fig. 4b), indicative of uniaxial order. Upon application of strain, the splitting of the Maltese cross into two melatopes indicates biaxial order, consistent with observations for nematic auxetic LCEs^10^.

As conoscopy can only visualise biaxiality up to relatively low strains (after which the melatopes leave the field of view), the order parameters were measured for the LCEs at strains up to 120% using polarised Raman Spectroscopy. Figure 4 c) shows the <P_200_> and <P_400_> order parameter(s) for the LCE samples as a function of strain. Plotting <P_200_> versus <P_400_> shows that as strain is increased, the order parameters reduce in magnitude and deviate from Maier-Saupe theory, a behaviour that can also be attributed to the emergence of biaxial order during strain, and again consistent with the observations made for nematic auxetic LCEs^11^. The in-plane director angle can also be monitored from the Raman scattering data, Fig. 4d. The behaviour observed is typical of that previously reported in LCEs as a mechanical Fréedericksz transition, now known to be associated with a biaxial deformation^10,11^.

These combined studies provide conclusive evidence that a biaxial deformation is driving the auxetic response in these frustrated smectic LCEs. We attribute the layer frustration, and the resulting softening of layer compression moduli, as being a driving factor that allows the biaxial response to continue to high strains, and hence for the auxetic response to be observed. It is also of note that some of the frustrated smectic LCEs have previously been examined under strain via X-ray scattering^12^, and show minimal evidence of buckling instabilities, or changes in layer spacing (with layer spacing varying by at most 0.5% during the experiment). However, these experiments are only able to elucidate information at small strains due to experimental difficulties, with more intensive studies set to be the focus of future work.

Despite the similarity of the auxetic response to that in nematic LCEs, it is expected that other aspects of the mechanical behaviour of SmA materials will be quite different. This is because the existence of smectic layers modifies the compression and shear moduli since changes in the smectic layer spacing are generally energetically unfavourable. We chose to examine the mechanical behaviour of two frustrated smectic LCEs through their shear moduli as such measurements offer an approach to also consider their anisotropic adhesion, a behaviour that is of increasing interest as an application area for LCEs^25,26,28^. In particular, here we chose to compare LCE1 and LCE3; although the former could not be strained sufficiently to observe an auxetic response, it allows a direct comparison to adhesion behaviour in an analogous nematic LCE previously reported^28^ (with a 6-carbon spacer, denoted here AN06^8^).

The distinct mechanical behaviour of SmA LCEs is revealed by considering the free-energy density (*F*) under a shear deformation (Eq. (1)), as was originally described in detail by Adams and Warner and is reproduced here for clarity^13^.

1 $$F=\frac{1}{2}\mu {{{\rm{Tr}}}}\{\underline{\underline{{l}_{0}}}\cdot \underline{\underline{{\lambda }^{T}}}\cdot \underline{\underline{{l}^{-1}}}\cdot \underline{\underline{\lambda }}\}+\frac{1}{2}b{\left(\frac{1}{|\underline{\underline{{\lambda }^{-T}}}\cdot {n}_{0}|}-1\right)}^{2}$$

The first term is the same trace formula as in nematic LCEs where µ is the linear shear modulus, $\underline{\underline{l}}$ the step length tensor which is expressed in terms of the step length anisotropy, *r*, $\underline{\underline{l}}$ = Diag(1, 1, r), $\underline{\underline{\lambda }}$ is the deformation tensor and $\underline{\underline{{\lambda }^{T}}}$ is the transpose of $\underline{\underline{\lambda }}$ ^13^. The second term relates to the smectic layer spacing, with layer spacing modulus B=b/μ, and the initial director is defined along the *z*-axis, i.e. ${n}_{0}={({{\mathrm{0,0,1}}})}^{T}$. The expression $1/|\underline{\underline{{\lambda }^{-T}}}\cdot {n}_{0}|$ represents the smectic layer spacing *d* relative to the unstrained spacing ${d}_{0}$, $d/{d}_{0}$^13,15^. Estimates for *r* and *b* can be ascertained from thermal expansion and tensile measurements and take values of r=2.1±0.5 and b=7.5±0.1, 3.4±0.1, and 5.7 ±0.1 for LCE1, LCE2, and LCE3 respectively^8^.

**Fig. 5:**
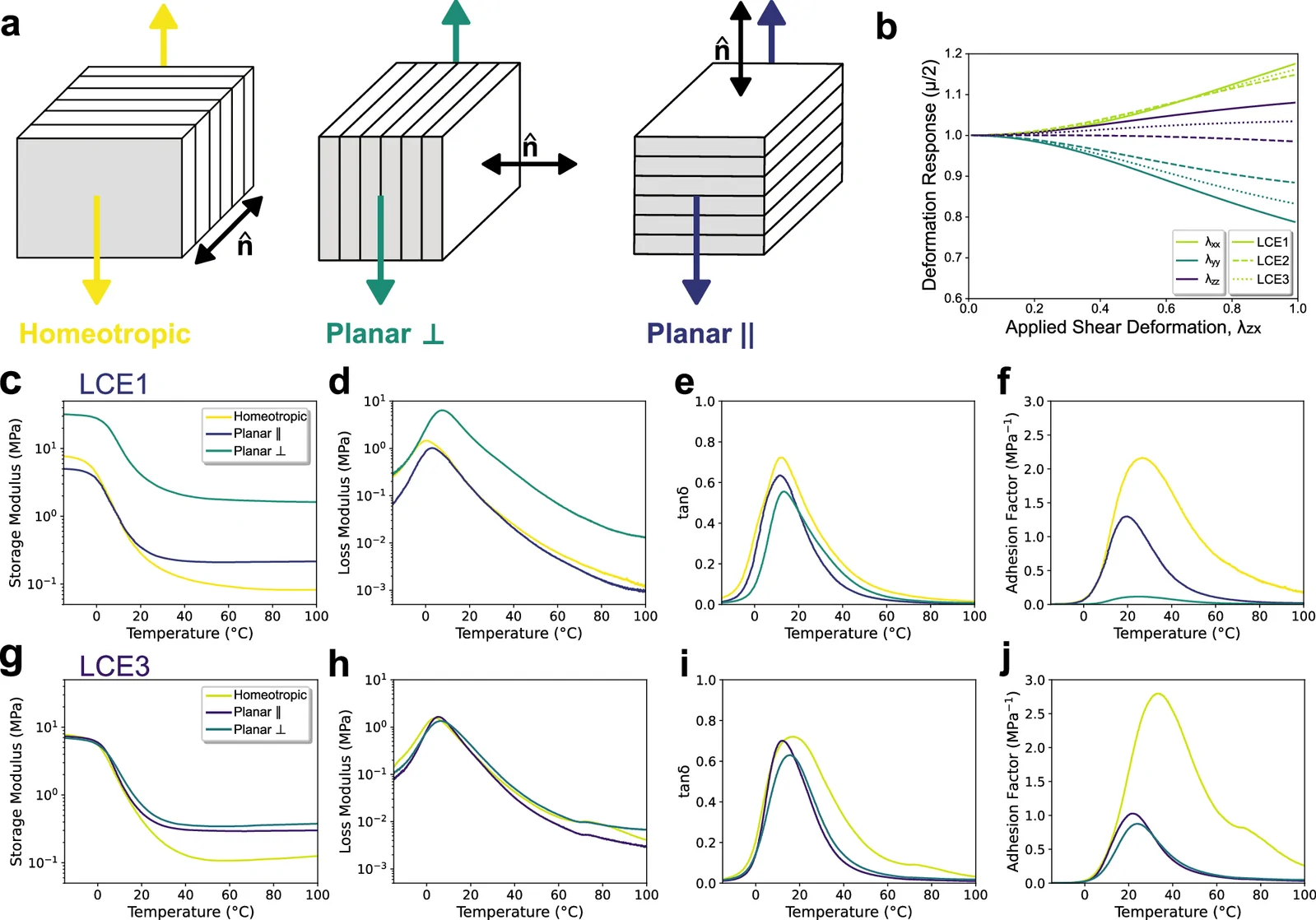
Shear mechanical properties of LCEs. **a** All possible geometries showing the relationship between the smectic layers and applied shear (arrows), with homeotropic (orange), planar ⊥ (green), and planar ∥ (red). **b** For the planar || case, the applied shear deformation is,$\,{\lambda }_{{zx}}$ and the minimised principal deformations in ${\lambda }_{{xx}}$ (turquoise), ${\lambda }_{{yy}}$ (pink), and ${\lambda }_{{zz}}$ (blue). The calculation is for a step length anisotropy *r* = 2.1 and smectic layer compression moduli *b* = 7.5 (solid), *b* = 3.4 (dotted), and *b* = 5.2 (dashed) which represent LCE1, LCE2, and LCE3 respectively^29^. **c**–**e**, **g**–**i** Show the storage modulus G’, the loss modulus G”, and dissipation tanδ = G”/G’, of LCE1 and LCE3 respectively, determined through shear dynamic mechanical analysis at 1 rads^−1^. **f**, **j** present an adhesion factor A = tanδ/G’ determined from the shear DMA data. For **c**–**j**, the different film alignments are identified through homeotropic (orange), planar ∥ (red), and planar ⊥ (green).

The different geometries in which it is possible to apply shear to SmA LCE films are shown in Fig. 5a. The relationship between the smectic layer normal (and therefore the initial director) and the applied shear has significant impact on the measured properties. In the homeotropic geometry, the layer normal is orthogonal to the shear, leading to an upper triangular deformation tensor (Eq. (2))^13^,

2 $$\underline{\underline{\lambda }}=\left(\begin{array}{ccc}{\lambda }_{xx} & 0 & {\lambda }_{xz}\\ 0 & {\lambda }_{yy} & 0\\ 0 & 0 & {\lambda }_{zz}\end{array}\right)$$

Utilising volume conservation, ${\lambda }_{{xx}}{\lambda }_{{yy}}{\lambda }_{{zz}}=1$, the free energy can be expressed as Eq. (3):

3 $$F=\frac{1}{2}\mu \left\{{\lambda }_{{xx}}^{2}+{\left({\lambda }_{{xx}}{\lambda }_{{zz}}\right)}^{-2}+{\lambda }_{{zz}}^{2}+r{\lambda }_{{xz}}^{2}+b{\left({\lambda }_{{zz}}-1\right)}^{2}\right\}$$

There is no coupling between the applied shear and the smectic layer modulus, so the free energy minimises to (Eq. (4)) where ${\lambda }_{{xx}}={\lambda }_{{zz}}=1$:

4 $$F=\frac{1}{2}\mu \left\{3+\,r{\lambda }_{{xz}}^{2}\right\}$$

This is the same as the nematic case, consequently there is no expected ‘punishment’ to the softness of the shear response in this geometry; it is expected to be analogous in nematic and SmA LCEs.

The planar ∥ geometry possesses the lower-triangular equivalent deformation tensor (Eq. (5))^13^:

5 $$\underline{\underline{\lambda }}=(\begin{array}{ccc}{\lambda }_{xx} & 0 & 0\\ 0 & {\lambda }_{yy} & 0\\ {\lambda }_{zx} & 0 & {\lambda }_{zz}\end{array})$$

The free energy now shows clear coupling between the applied shear and the evolution of the smectic layers (Eq. (6)).

6 $$F=\frac{1}{2}\mu \left\{{\lambda }_{{xx}}^{2}+\frac{1}{{\lambda }_{{xx}}^{2}{\lambda }_{{zx}}^{2}}+{\lambda }_{{zx}}^{2}+\frac{{\lambda }_{{zx}}^{2}({\lambda }_{{xx}}^{2}+r{\lambda }_{{zx}}^{2})}{{\lambda }_{{xx}}^{2}+{\lambda }_{{zx}}^{2}}+b{\left(\frac{{\lambda }_{{xx}}{\lambda }_{{zx}}}{\sqrt{{\lambda }_{{xx}}^{2}+{\lambda }_{{zx}}^{2}}}-1\right)}^{2}\right\}$$

Minimising the free energy requires numerical methods and the solution when r=2.1 and b=7.5 (solid), 3.4 (dashed), and 5.7 (dotted) are shown in Fig. 5b, where values of b have previously been determined experimentally through tensile strain measurements and fitting to the equation λ_xx_^2^λ_zz_^2^−1=b(1- λ_xx_λ_zz)_ (Equation SE3)^13^. There is coupling between the shear and the layer spacing, which is manifest as an increased modulus^13^. To continue beyond a shear deformation of ~0.2 the deformation along the layer normal begins to increase, LCE1 requires a higher deformation than LCE2 and LCE3–offering an explanation as to why LCE1 consistently fails at relatively low strains whereas LCE2 and LCE3 do not. LCE2, with the lowest layer spacing modulus, has a negligible increase in ${\lambda }_{{zz}}$ and is able to accommodate large changes in strain which gives freedom for the auxetic response to occur.

Figure 5c–e, g–i show the shear storage, $G{\prime}$, and loss, $G{\prime} {\prime}$, moduli measured for the SmA materials LCE1 and LCE3, together with the dissipation ratio, $\tan \delta={G}^{{\prime} }{\prime} /G{\prime}$, determined for an oscillation frequency of 1 rads^−1^ across the temperature range from −15 °C to 100 °C. The storage moduli take similar values for both materials plateauing above room temperature to values between around 0.1–1.0 MPa, but with a much higher anisotropy observed for LCE1 where the planar perpendicular case takes values around an order of magnitude higher than either of the other two geometries. The moduli are slightly higher than those reported for the nematic analogue, AN06^28^. There is a single peak in $\tan \delta$ which occurs at 12 ˚C in LCE1 and 15 ˚C in LCE3 that is associated with the glass transition. An adhesion factor, correlated with the peel and tack strength of LCEs used as pressure sensitive adhesives, can be defined by Eq. (7). The shear moduli are measured at small strain, and the assumption is made that the surface energy of each LCE type is the same across the geometries, which is valid for the nematic case.

7 $$A=\frac{\tan \delta }{G{\prime} (1\,{rad}{s}^{-1})}=\frac{G{\prime} {\prime} (1\,{rad}{s}^{-1})}{{(G{\prime} (1{rad}{s}^{-1}))}^{2}}$$

The adhesion factor is shown in Fig. 5f, j, for LCE1 and LCE3 respectively. There is significant anisotropy in the adhesion factors, with the most dramatic variation observed between the homeotropic (stickiest) and planar perpendicular (least sticky) versions of LCE1 where there is a factor of ~20 (LCE1) or ~3 (LCE3) difference between the two geometries. While the absolute values of the adhesion factor cannot be directly compared between the materials because of the contributions of surface energy to adhesion, it is noteworthy that the values obtained in the homeotropic case for LCE1 and for LCE3 are comparable in magnitude to the adhesion factor of an analogous homeotropic nematic system^28^. This result is in keeping with the chemical similarity of LCE3 and the material AN06 considered by Street et al.^28^ (the side group has a mesogenic moiety identical to LCE1 and 6-carbon spacer in common with LCE3), as well as the theoretical explanations presented earlier (Eq. (4)), where the shear behaviour in the homeotropic SmA case is seen to be the same as in the nematic case. Simply speaking, this result can be thought of as a consequence of the fact that a change in layer spacing is not needed to shear the homeotropic smectic LCE, unlike the planar cases.

In addition to calculating a temperature-dependent adhesion factor, the experimentally-determined adhesion properties of LCE1 and LCE3 were compared at room temperature with those of the nematic LCE AN06 reported by Street et al.^28^. First, peel tests, where a strip of LCE is peeled away from a glass surface at 90 °, were performed on homeotropic and planar samples of LCE3 with the planar director perpendicular to the peeling direction. The force per unit width required to debond LCE3 from the substrate is 0.0091 ± 0.004 Nmm^-1^ and 0.0140 ± 0.004 Nmm^-1^ in the planar and homeotropic cases respectively. The factor of 1.5 found between the homeotropic and planar cases compares well with the 2.0 times difference suggested by the adhesion factor at room temperature. This can be compared to literature results for the nematic AN06 where the planar orientation adheres more strongly than the homeotropic (0.043 Nmm^-1^ and 0.015 Nmm^-1^ respectively), values which are again strongly correlated with the adhesion factor. ^28^

**Fig. 6:**
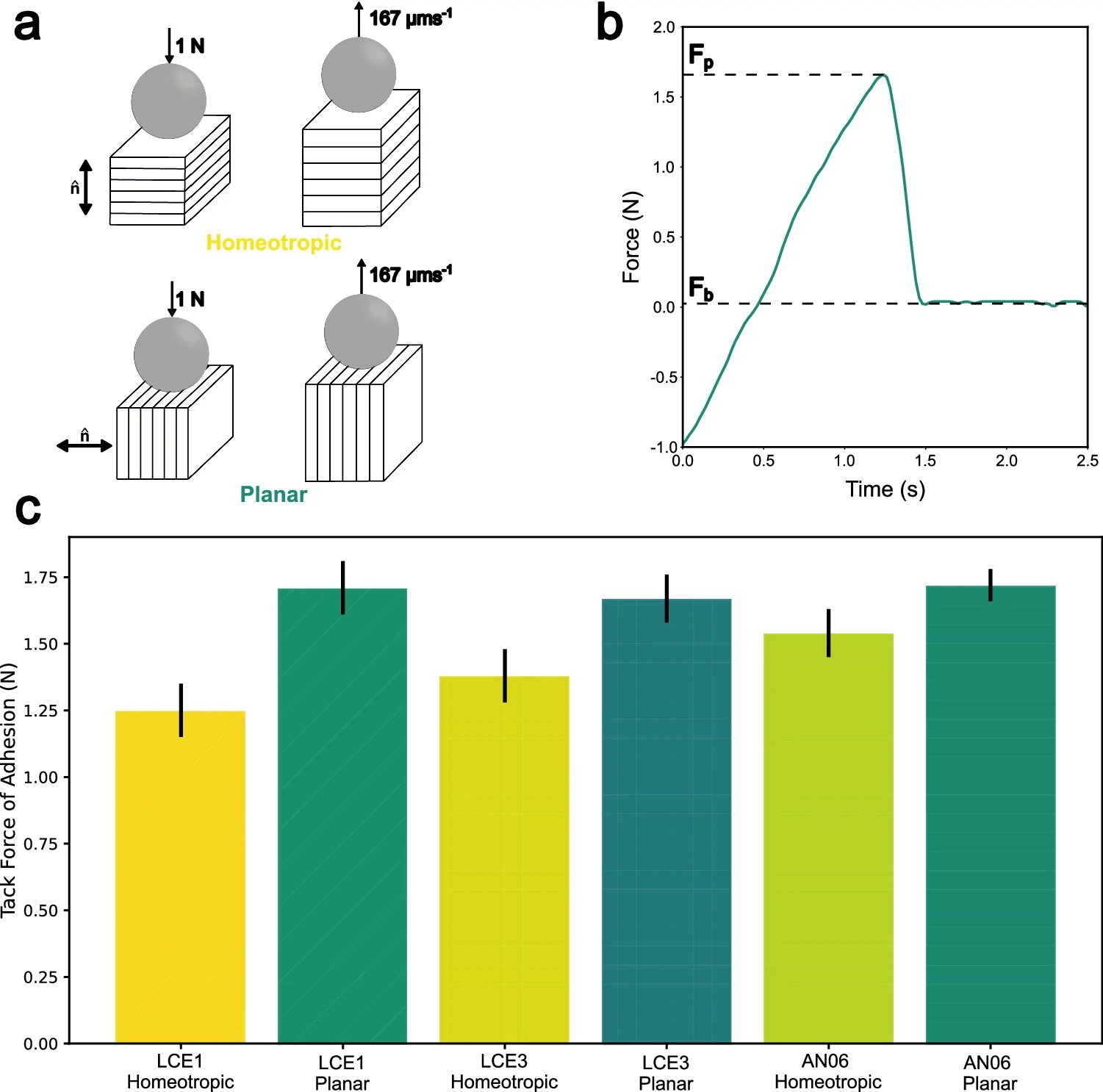
Tack test results for the LCEs. **a** Schematic of the spherical probe being applied and removed from LCE samples aligned in homeotropic (orange) and planar (green) geometries. In the homeotropic case the removal force is parallel to the director and layer normal compared to the planar case where the removal force is perpendicular to the director. **b** A typical tack-test result, after a dwell-time 30 s at a force of −1 N, the probe is removed and the force measured. The tack force of adhesion is the difference between the peak force (F_p_) and the baseline force (F_b_). **c** Tack force of adhesion results for LCE1 (diagonal hatches), LCE3 (square hatches), and NLCE (horizontal hatches), for homeotropic (orange) and planar (green) alignments. The error bars represent the standard error in the data for *n* = 3.

The adhesive properties of LCE1 and LCE3 were also probed through tack tests. Due to the geometry of the probe-tack test, removing the probe from a homeotropic LCE will incur the maximum penalty caused by the layer compression modulus (Fig. 6a), and the planar LCE will incur a minor penalty. Figure 6b shows a typical tack result where the spherical probe is removed from the sample at a constant speed and the force measured. Figure 6c shows the average tack force of adhesion for LCE1, LCE3, and AN06 reported by Street et al.^28^. In the planar alignment the tack strength is effectively the same for LCE1, LCE3, and AN06 (1.7 ± 0.1 N, 1.67 ± 0.09 N, and 1.72 ± 0.06 N) respectively. In the homeotropic geometry the tack strength in AN06 (1.54 ± 0.09 N) is similar to the tack strength in LCE3 (1.4 ± 0.1 N) and is significantly larger than in LCE1 (1.25 ± 0.09 N). This is attributed to the layer spacing modulus, b, which is much lower in LCE3 (*b* = 3.4 ± 0.1) than in LCE1 (*b* = 7.5 ± 0.1), influencing the anisotropic adhesion less. It is interesting to explore this finding in the context of the shear moduli; the peak magnitude of $\tan \delta$ is comparable for all three of the materials being considered at around 0.6–0.8, though the average value at room temperature (where the peel tests were performed) is 0.50, 0.64, and 0.72 for LCE1, LCE3, and AN06 (Street et al.) respectively. We note that in the planar configuration all LCEs are similar in tack despite the differences in $\tan \delta$ (0.55, 0.70, and 0.72 in LCE1, LCE3, and AN06) respectively). However in the homeotropic geometry, where the room temperature values of $\tan \delta$ are 0.45, 0.57, and 0.72, there is a positive correlation between the measured tack and $\tan \delta$. The ratio of the tack strength for the homeotropic and planar geometries is 1.4 and 1.2 for LCE1 and LCE3 respectively. This compares well with the 2.0 factor predicted at room temperature by the adhesion factor as determined via the shear moduli.

A final point to note is that these particular LCEs do not satisfy the Dahlquist criterion (${G}^{{\prime} } < 0.11{{{\rm{MPa}}}}$ and *T*_g_ ~ 0 °C) and so would not be suitable for use as PSAs at room temperature^28,34^, however the anisotropy in adhesion is starkly different to nematics which suggests that smectic adhesives may be an avenue for future PSA design.

## Discussion

When considering the results presented herein as a whole, this work offers considerations for both the controlled fabrication of smectic LCEs, as well as innovative approaches to the design of materials for applications. Predictions were made by de Gennes for the templating of smectic macromolecules in either nematic or smectic solvents:

1. *In the nematic case, if the chains are elongated in the direction of the nematic axis, we obtain, during crosslinking, a system stretched in this direction. But, during washing, the network can relax without major obstacle towards an isotropic conformation*^16^.
2. *in the smectic case where the chains are intercalated between the layers of the solvent, we would obtain … a very anisotropic material from the mechanical point of view*.^16^

We have tracked the evolution of the order present in the LCEs formed in a nematic solvent throughout the fabrication process, allowing us to elucidate the mechanism behind the frustration in these LCEs. Our x-ray scattering results show that the smectic ordering develops during curing and is retained with a comparable correlation length after the removal of unreactive nematic solvent. Thus, we can confirm that the formation of smectic layers in the LCEs is a direct result of the curing process (case 1 above) and that we might expect the mechanical properties of such a system to differ significantly from both nematic LCEs and smectic systems fabricated in case 2. This finding has exciting implications for the wider LCE research field, suggesting that LCE systems could be obtained that previously have not been observed. As has been reported here, this could in turn lead to innovative behaviours for LCEs, expanding their scope for applications.

Further to increasing our understanding of the evolution of frustration in LCEs, this work presents another family of LCEs with negative Poisson’s ratio, explicitly showing that auxetic behaviour at a molecular level is not limited to nematic LCEs^8,9,35^. Three of the smectic formulations reported are sufficiently robust to allow strain into an auxetic regime. In all of these cases, the LCE was formed in a non-smectic precursor mixture and we suggest that the frustrated nature of the resulting SmA LCE is in part responsible for the ability to achieve high strains and therefore observe auxeticity.

The importance of the discovery of these auxetic systems is rooted in the increased scope for molecular design that these findings imply. It had previously been expected that only nematic LCEs could exhibit auxetic behaviour as the auxetic threshold strains were higher than the typical strain-failure observed in smectic LCEs (~30%)^8^. However, the requirement of a nematic phase is a serious constraint; side-chain liquid crystal polymer systems such as the LCEs reported here are often dominated by smectic character. The observation of auxetic behaviour in smectic LCEs provides impetus to reconsider side groups that would previously have been considered as too smectogenic, expanding the scope of structural motifs that can be incorporated into molecular auxetics. This broader palette of monomers provides a wider basis with which to optimise material properties for specific applications. Whilst such studies are the focus of future work, improved material properties are already being seen in the frustrated smectic LCEs reported herein; for example, the auxetic threshold observed for LCE3 is the lowest reported for any auxetic LCE at a comparable strain rate. Auxetic smectic LCEs also offer moduli that are different both in magnitude and anisotropy from nematic systems, providing another dimension of modification for potential applications.

It has been established that the threshold strain at which the auxetic response is observed in nematic LCEs is dependent on dynamics, particularly the proximity to the glass transition temperature (*T*_g_) of the sample^8,33^. This manifests as materials with lower *T*_g_ exhibiting a lower auxetic threshold, when strained at a given temperature^8^. The situation appears to be more complex in these auxetic SmA LCEs as lowering *T*_*g*_ doesn’t necessarily result in a lower auxetic threshold. However, within this work we provide evidence to support a dependence on dynamics in the frustrated smectic LCEs; Increasing the rate at which the LCE is strained generally leads to an increase in the observed auxetic threshold, consistent with the nematic case^33^.

It is clear that one of the ‘nematic-like’ behaviours that can be retained in SmA LCEs is the potential to deform biaxially and therefore exhibit auxeticity. Although the anisotropic adhesion factors were considerably lower than have been observed in nematic LCEs, it is reasonable to suggest that approaches such as increasing spacer lengths which will modify *T*_g_ might be exploited in order to produce pressure sensitive SmA LCE adhesives that are able to satisfy the Dahlquist criterion. Through peel testing and tack testing, a high anisotropy was shown in the adhesion of SmA LCEs where the contribution of layer compression/expansion greatly affects the adhesive strength of films. Indeed, the smectic nature of the LCEs ensure a high anisotropy in the adhesion factors, potentially expanding the scope of LCE adhesive development that utilise smectics.

## Methods

### Materials

The 8-(4-Cyano-biphenyl-4’-yloxy)octyl acrylate (M1) used in this work was synthesised in accordance with previous literature^8^. The 4-[[6-(Acryloyloxy)hexyl]oxy]benzoic acid (A6OBA) was purchased from Sigma Aldrich (Guangzhou, China), dissolved in dichloromethane (DCM), and filtered through a 0.22 µm syringe filter to remove insoluble impurities. All other materials were used as purchased without further purification, and were obtained from one of the following suppliers: Sigma Aldrich (Gillingham, UK), Fisher Scientific (Loughborough, UK), Apollo Scientific (Stockport, UK), Ambeed (Arlington Heights, IL, USA), Fluorochem (Glossop, UK), Synthon Chemicals GmBH (Wolfen, Germany).

Monodomain films of LCE1, LCE2 and LCE3 were fabricated in both planar and homeotropic geometries (Fig. 1c, d) and seen to be transparent at strains up to 60% (Fig. 1e). As the order in LCE4 was induced through an applied mechanical strain, only planar samples were produced. The formulations used are depicted in Fig. 2 and the film production methods for all LCEs are described in detail in the methods section. The final films had glass transition temperatures below room temperature in all cases (see Table S1).

### LCE mold fabrication

The LCEs were synthesised in bespoke moulds, which were made in accordance with previous literature^5,8,10^. For the planar samples, glass microscope slide (7.5 cm × 2.5 cm × 1 mm), and a Melinex® ST725 substrate (7 cm × 2.5 cm × 250 µm) (DuPont Teijin Films, Redcar, UK) were spin coated on one surface with an aqueous 0.5 wt% polyvinyl alcohol (PVA) solution. After drying the coated substrates at 50 °C for 10 min, the substrates were uniaxially rubbed with a bespoke rubbing machine. These two substrates were then adhered, via Melinex® 401 spacers (7.5 cm × 0.2 cm × 100 µm) (DuPont Teijin Films, Redcar, UK) and UVS-91 adhesive, so that the PVA-rubbed surfaces were the inner surfaces of the constructed cell, and the rubbing directions yielded antiparallel planar alignment along the width of the mould. The adhesive was then cured by irradiation under 350 nm (2.5 Wcm^−2^) at 50 °C for 10 min, to yield the constructed LCE mould with a gap of ~100 µm thickness. For the homeotropic samples, the construction is identical except that the substrates also had ITO electrodes and a homeotropic alignment agent, Cetyltrimethylammonium Bromide (CTAB) was used in place of the PVA^10^. A voltage applied during polymerisation (40 V_rms_) supported the homeotropic alignment in the 100 μm thick layer^10^.

### LCE film synthesis

LCE1, LCE2, and LCE3 were fabricated in accordance with previous literature^8,12^, from the generalised mixtures displayed in Fig. 2. In a typical procedure, 2-Methyl−1,4-phenylene bis(4-((6-(acryloyloxy)hexyloxy)benzoate (RM82) (3.5 mol%), the appropriate monofunctional monomer (M1/M2/M3)(24.4 mol%), and 4’-Hexyloxy-(1,1’-biphenyl)-4-carbonitrile (6OCB) (54.6 mol%) were heated to 120 °C with stirring until a homogeneous isotropic phase was obtained. The mixture was cooled to 45 °C, followed by the addition of 2-ethylhexyl acrylate (EHA) (16 mol%) and methylbenzoyl formate (MBF) (1.5 mol%), and stirred for 5 min, ensuring a homogeneous, completely isotropic material was obtained. The precursor mixtures for LCE1, LCE2, and LCE3 all exhibited only nematic phases with the transition to the isotropic phase, T_NI_, reported in Table S1. The mixture was then filled into a mould at 50 °C via pipette, before being cooled to room temperature (23 C) and allowed to stand for 30 min. The samples were then cured in the nematic phase under 350 nm (2.5 Wcm^−2^) irradiation for 2 h. After curing, the samples were removed from the moulds (using a small amount of isopropanol if necessary to aid delamination from the substrates), and left to stand in a solution of DCM:isopropanol (30:70) overnight to remove the non-reactive 6OCB. The samples were then allowed to dry under ambient conditions for 5 h, to yield the LCE films.

LCE4 was fabricated using the strain-induced alignment methodology frequently employed in the literature^36,37^. A mixture of RM82 (2.5 mol%), 6OBA (5 mol%), A6OCB (51 mol%), EHA (40.3 mol%) and MBF (1.2 mol%) was heated to 85 °C and filled into the LCE mould. The sample was then cured at 85 C under 350 nm (2.5 Wcm^2^) irradiation for 2 h. The polymerised sample was then heated to 100 °C and strained to 60% (1.6x times its original length) to induce liquid crystalline order and cooled to room temperature to lock the alignment.

### Material characterisation

#### X-Ray diffraction

Small- (SAXS) and Wide- (WAXS) angle X-ray scattering experiments were performed on an Anton Paar SAXSpoint 5.0 system (K-α Cu source, λ = 1.5418 Å) with a Dectris EIGER2 R 1 M (1028 pixel × 1062 pixel array) detector. For LCE films, measurements were performed at room temperature on ~100 µm thick samples averaging 3 frames with exposures times of 300 s. For the precursor mixture, the nematic fluid was held in a QGCT 1.0 quartz capillary (1.0 mm diameter) (Capillary Tube Supplies Ltd., Cornwall, UK), averaging 3 frames with exposure times of 300 s each. A 2 mm beam size was used. A background scan was performed for both the SAXS and WAXS detector position which was subtracted from the measurements to minimise contributions from intrinsic background scattering and the Mylar protective film in front of the detector.

The correlation lengths parallel (${{{{\rm{\xi }}}}}_{{||}}$) to the director were deduced from SAXS data, according to Eq. (8), as was recently used by Kennedy et al. for LCEs^38^. This equation uses the full width half maximum (${w}_{{||}}$) of Gaussian fittings to the scattering intensity of the q ~ 1.6 nm^−1^ feature (the (001) Bragg peak due to the smectic layer spacing).

8 $${\xi }_{{||}}=\frac{2\pi }{{w}_{{||}}}$$

The scalar nematic order parameter, *S*, was calculated from the 2D WAXS pattern using the Kratky method^39^ shown in Eq. (9):

9 $$I\left(\chi \right)=	 {f}_{0}+\frac{1}{2}{f}_{2}{\cos }^{2}\left(\chi \right)+\frac{3}{8}{f}_{4}{\cos }^{4}\left(\chi \right)+\frac{5}{16}{f}_{6}{\cos }^{6}\left(\chi \right) \\ 	+\frac{35}{128}{f}_{8}{\cos }^{8}\left(\chi \right)+\frac{63}{256}{f}_{10}{\cos }^{10}\left(\chi \right)$$

Where *I* is intensity, *χ* is the azimuthal angle orthogonal with respect to the nematic director and *f*_n_ are constants determined by fitting Eq. (9). The *f*_n_ constants are used to determine 〈${\cos }^{2}(\beta )$〉 through Eq. (10):

10 $$\left\langle {\cos }^{2}\left(\beta \right)\right\rangle=\frac{{\sum }_{n=0}^{\infty }\frac{{f}_{2n}}{2n+3}}{{\sum }_{n=0}^{\infty }\frac{{f}_{2n}}{2n+1}}\,$$

where *β* is the angle between the axis of a molecule and the nematic director. Thus $S=\left\langle {P}_{2}\right\rangle$ can be determined through the Eq. (11):

11 $$S=\frac{1}{2}\left(3\left\langle {\cos }^{2}\left(\beta \right)\right\rangle -1\right)$$

In calculating *S*, the baseline was removed using a zero-offset method.

#### Macroscopic shape change and measurements of Poisson’s ratio

A bespoke mechanical strain apparatus which has been described in detail in previous publications^5^ consisting of two actuators capable of extending a sample with a minimum step size of 0.5 mm was used to determine the strain-strain behaviour of the LCE films. The apparatus allows for polarising microscopy and bright field images to be taken of the top-down view of the LCE, enabling a semi-quantitative analysis of the material optical properties including birefringence and transparency as a function of strain. LCE strips of approximate dimensions 20 mm × 2 mm × 100 μm were strained until failure at a rate of 0.05 mm min^−1^. Features in the LCE were tracked using imageJ to deduce the strain in the length and the thickness of the film, and a constant volume condition used to determine the strain in the thickness. The instantaneous Poisson’s ratio is deduced from the strain data as described in detail previously^10^.

#### Dynamic mechanical analysis

A TA Instruments Dynamic Mechanical Analysis 850, equipped with steel shear sandwich clamps, was used to shear two square samples of length 7 mm and thickness 100 μm using a centre movable plate. All measurements were performed in the linear viscoelastic range, as confirmed by performing an initial strain sweep. A shear oscillatory frequency of 1 rads^−1^ and temperature range of 100 °C cooling to −15 °C at 2.0 °Cmin^−1^ was used. The recorded temperature is for the air surrounding the sample and may be shifted compared to the internal temperature of the LCE, all measurements are self consistent and comparable. All experiments were performed three times, with a representative dataset chosen for analysis.

#### Polarised Raman spectroscopy

A Renishaw inVia Raman microscope in backscattering geometry was used with a 532 nm solid-state laser of laser power 2.5 mW to determine the Raman spectra of LCE1, LCE2, and LCE4. A 50x objective was focused onto the sample. The LCE samples were placed inside a manual straining rig and measurements taken in 10° intervals through a full 360° rotation for light polarised both parallel and perpendicular to the analyzer, allowing the order parameters to be deduced^11^.The director angle during strain was determined from the angular dependence of the phenyl breathing mode (Raman shift 1606 cm^−1^) mode in the Raman spectra^11^.

#### 90° peel measurements

The methodology previously described by Street et al. was used to determine the peel strength of the LCEs^28^. Briefly, glass slides were cleaned via sonication in isopropyl alcohol for 30 min. LCE strips of nominal dimensions 15 mm × 5 mm × 100 μm (homeotropic) and ~20 mm × 14 mm × 100 μm (planar) were adhered to the glass using a force of 3.9 kN for 5 min at room temperature (20.5 °C). To peel the LCE, a variable mass was attached to the underside of the LCE, the mass was increased until steady-state peeling was achieved with three repeats. The mass required to achieve peel was divided by the exact width of the sample to calculate the relative debonding force in units of Nmm^−1^. All LCE strips were removed through adhesive failure, with no residue left behind.

#### Probe tack measurements

A spherical stainless steel probe of diameter 25 mm was cleaned using isopropyl alcohol, and pressed into the surface of a LCE with a downward force of −1 N for a dwell period of 30 s using an Instron 5564 universal testing machine at room temperature (20 °C). After the dwell period the probe was removed at a constant speed of 167 μms^-1^. The dwell period and probe removal speed were chosen to match literature conditions that have been employed for other LCE chemistries^25,26,28,30^. The force of adhesion was determined from the maximum force recorded before separation (adhesive failure).

## Supplementary information

Supplementary Information

Peer Review File

## Data Availability

The data generated in this study have been deposited in the University of Leeds data repository, under the https://doi.org/10.5518/1851. Additionally, all data are available from the corresponding author upon request.

## References

[1] Evans, K. E. Auxetic polymers: a new range of materials. *Endeavour* **19**, 170–174 (1991).

[2] Ren, X., Das, R., Tran, P., Ngo, T. D. & Xie, Y. M. Auxetic metamaterials and structures: a review. *Smart Mater. Struct.* **27**, 023001 (2018).

[3] Turincio, A. Puma enters new era with cushioning technology Xetic. Puma SE, https://about.puma.com/en/newsroom/news/puma-enters-new-era-cushioning-technology-xetic (2020).

[4] Head GmbH. Auxetic 2.0 - The Science Behind the Sensational Feel. HEAD Sport GmbH, https://www.head.com/en/rs/stories/auxetic-the-science-behind-the-sensational-feel (2026).

[5] Mistry, D. et al. Coincident molecular auxeticity and negative order parameter in a liquid crystal elastomer. *Nat. Commun.* **9**, 5095 (2018). 10.1038/s41467-018-07587-y PMC6279820 30514842

[6] Cooper, E. J. et al. Controlling the optical properties of transparent auxetic liquid crystal elastomers. *Macromolecules* **57**, 2030–2038 (2024). 10.1021/acs.macromol.3c02226 PMC10938889 38495386

[7] Moorhouse, T. & Raistrick, T. Sub-micron diffractive optical elements facilitated by intrinsic deswelling of auxetic liquid crystal elastomers. *Adv. Opt. Mater.* **12**, 2400866 (2024).

[8] Berrow, S. R., Raistrick, T., Mandle, R. J. & Gleeson, H. F. Structure–property relationships in auxetic liquid crystal elastomers—the effect of spacer length. *Polymer* **16**, 1957 (2024). 10.3390/polym16141957 PMC11280505 39065273

[9] Berrow, S. R., Raistrick, T., Mandle, R. J. & Gleeson, H. F. Auxetic liquid crystal elastomers: overcoming barriers to scale-up. *ACS Appl. Polym. Mater.* **7**, 4517–4524 (2025). 10.1021/acsapm.5c00212 PMC11997952 40242050

[10] Wang, Z. et al. Direct observation of biaxial nematic order in auxetic liquid crystal elastomers. *Materials* **16**, 393 (2023). 10.3390/ma16010393 PMC9822019 36614732

[11] Raistrick, T., Zhang, Z., Mistry, D., Mattsson, J. & Gleeson, H. F. Understanding the physics of the auxetic response in a liquid crystal elastomer. *Phys. Rev. Res.* **3**, 023191 (2021).

[12] Berrow, S. R. et al. The effect of templating on the mechanical properties of smectic liquid crystal elastomers. *J. Mater. Chem. C. Mater.* **13**, 12365–12375 (2025).

[13] Adams, J. M. & Warner, M. Elasticity of smectic-A elastomers. *Phys. Rev. E Stat. Nonlin. Soft Matter Phys.* **71**, 021708 (2005). 10.1103/PhysRevE.71.021708 15783341

[14] Adams, J. M. & Warner, M. Soft elasticity in smectic elastomers. *Phys. Rev. E Stat. Nonlin. Soft Matter Phys*. **72**, 011703 (2005). 10.1103/PhysRevE.72.011703 16089983

[15] Warner, M. & Terentjev, E. M. *Liquid Crystal Elastomers* (Oxford University Press, 2003).

[16] De Gennes, P. G. Possibilities Offertes Par La Reticulation De Polymeres En Presence D’un Crystal Liquide. *Phys. Lett.* **28A**, 725–727 (1969).

[17] Ujiie, S. & Kato, T. Design and synthesis of side-chain liquid crystal polymers. in *Handbook* of Li*qu*id Cr*ystals* (eds. Goodby, J. W. et al.) vol. 7, 381–411 (Wiley-VCH, 2014).

[18] De Gennes, P. G. Reflexions sur un type de polymeres nematiques. *C. R. Acad. Sci. Ser. B* **281**, 101–103 (1975).

[19] Clarke, S. M. et al. Soft elasticity and mechanical damping in liquid crystalline elastomers. *J. Appl. Phys.* **89**, 6530–6535 (2001).

[20] Merkel, D. R., Shaha, R. K., Yakacki, C. M. & Frick, C. P. Mechanical energy dissipation in polydomain nematic liquid crystal elastomers in response to oscillating loading. *Polymer* **166**, 148–154 (2019).

[21] Saed, M. O. et al. Impact damping and vibration attenuation in nematic liquid crystal elastomers. *Nat. Commun*. **12**, 6676 (2021). 10.1038/s41467-021-27012-1 PMC8602409 34795251

[22] Terentjev, E. M. Liquid crystal elastomers: 30 years after. *Macromolecules* **58**, 2792–2806 (2025). 10.1021/acs.macromol.4c01997 PMC11948470 40160994

[23] Lagerwall, J. Liquid crystal elastomer actuators and sensors: Glimpses of the past, the present and perhaps the future. *Program. Mater.* **1**, 1–46 (2023).

[24] Guin, T. et al. Layered liquid crystal elastomer actuators. *Nat. Commun.* **9**, 2531 (2018). 10.1038/s41467-018-04911-4 PMC6023890 29955053

[25] Ohzono, T., Saed, M. O. & Terentjev, E. M. Enhanced dynamic adhesion in nematic liquid crystal elastomers. *Adv. Mater.* **31**, 1902642 (2019). 10.1002/adma.201902642 31183920

[26] Pranda, P. A. et al. Directional adhesion of monodomain liquid crystalline elastomers. *ACS Appl. Mater. Interfaces* **16**, 6394–6402 (2024). 10.1021/acsami.3c16760 38266384

[27] Choi, S. et al. Harnessing extreme internal damping in polyrotaxane-incorporated liquid crystal elastomers for pressure-sensitive adhesives. *Adv. Funct. Mater*. **35**, 2413824 (2025).

[28] Street, A., Mistry, D., Mattsson, J. & Gleeson, H. F. Geometry-dependent adhesion in transparent monodomain liquid crystal elastomers. *Adv. Funct. Mater*. **36**, e22644 (2025).

[29] Mistry, D. et al. Isotropic liquid crystal elastomers as exceptional photoelastic strain sensors. *Macromolecules* **53**, 3709–3718 (2020).

[30] Guo, H., Saed, M. O. & Terentjev, E. M. Mechanism of pressure-sensitive adhesion in nematic elastomers. *Macromolecules* **56**, 6247–6255 (2023). 10.1021/acs.macromol.3c01038 PMC10448750 37637306

[31] Leadbetter, A. J., Frost, J. C., Gaughan, J. P., Gray, G. W. & Mosley, A. The structure of smectic A phases of compounds with cyano end groups. *J. Phys. Fr.* **40**, 375–380 (1979).

[32] Berrow, S. R., Mandle, R. J., Raistrick, T., Reynolds, M. & Gleeson, H. F. Toward monodomain nematic liquid crystal elastomers of arbitrary thickness through PET-RAFT polymerization. *Macromolecules* **57**, 5218–5229 (2024). 10.1021/acs.macromol.4c00245 PMC11171763 38882196

[33] Raistrick, T. et al. Resolving the mechanical response of liquid crystal elastomers – semi-soft elastic or auxetic. *Soft Matter* **21**, 8849–8866 (2025). 10.1039/d5sm00677e 41186386

[34] Chang, E. P. Viscoelastic windows of pressure-sensitive adhesives. *J. Adhes.* **34**, 189–200 (1991).

[35] Liu, M. & Zhao, Y. Unusual stretching-induced molecular orientation behavior of a side-chain liquid crystal elastomer and its reorientation-enabled auxeticity. *Macromolecules* **58**, 905–918 (2025).

[36] Herbert, K. M. et al. Synthesis and alignment of liquid crystalline elastomers. *Nat. Rev. Mater.* **7**, 23–38 (2022).

[37] Ding, J. et al. Locking alignment of liquid crystal actuators using hydrogen bonds to enable room-temperature shape programmability and enhanced work capacity. *Adv. Funct. Mater*. **35**, 2414510 (2025).

[38] Kennedy, D. T., Hoang, J. D., Toney, M. F. & White, T. J. Deconvoluting the contribution of polymerization temperature to liquid crystalline elastomer actuation. *Macromolecules* **57**, 10032–10040 (2024).

[39] Sims, M. T., Abbott, L. C., Richardson, R. M., Goodby, J. W. & Moore, J. N. Considerations in the determination of orientational order parameters from X-ray scattering experiments. *Liq. Cryst.* **46**, 11–24 (2019).

