# Supplementary material for: Frustrated smectic liquid crystal elastomers as multifunctional mechanical metamaterials
Source: Nat Commun. 2026 Sep 11;17:10726. doi: 10.1038/s41467-026-76776-x (PMC13658772; doi:10.1038/s41467-026-76776-x)
Supplement: Supplementary file 1 [file 41467_2026_76776_MOESM1_ESM.pdf]

Supplementary Information

Frustrated Smectic Liquid Crystal Elastomers as Multifunctional Mechanical Metamaterials

A. Street[1§], S. R. Berrow[1§*], Z. Wang,[1] E. J. Cooper[1], J. Mattsson[1], and H. F. Gleeson[1]

[1]School of Physics and Astronomy, University of Leeds, LS2 9JT

[§]These authors contributed equally

*S.R.Berrow@leeds.ac.uk

Supporting Information available: Further Experimental Information, Differential Scanning Calorimetry, Polarised light optical microscopy, Supplementary X-ray Scattering, Optical evaluation of the LCEs during strain, Polarised Raman spectroscopy.

Table Of Contents

Further Experimental Information 2
Differential Scanning Calorimetry 3
Polarised Light Optical Microscopy 5
Supplementary X-Ray Scattering 7
Polarised Raman Spectroscopy 10
Smectic Layer Compressibility Modulus Calculation 12
Supplementary References 12

Further Experimental Information

Optical Microscopy

Polarised light optical microscopy (POM) was performed using a Leica DM2700P polarised light microscope (Leica Microsystems (UK) Ltd., Milton Keynes, UK), equipped with 10x and 50x objectives, and a rotatable stage. For LCE samples, the films were mounted on a glass slide, and analysed under ambient conditions, using a 5x objective. For phase identification of the liquid crystal mixtures, a Mettler Toledo FP82HT Hot Stage (Mettler-Toledo Ltd., Leicester, UK), controlled by a Mettler Toledo FP90 central processor (Mettler-Toledo Ltd., Leicester, UK) was used to control the temperature of the sample with a relative accuracy of 0.1 °C. In this case, the sample was mounted between a glass microscope slide and a glass cover slip, and samples were analysed using 50x objective. Images were recorded using a Nikon D3500 Digital Camera (Nikon UK Ltd., Surbiton, UK), using DigiCamControl software. Conoscopy was carried out in transmission with a 50x objective and high numerical aperture condenser, allowing highly convergent light to be incident on the sample. A Bertrand lens imaged the back-focal plane, allowing observation of the conoscopic figure.

Thermal Analysis

Differential scanning calorimetry (DSC) measurements were performed using a TA Instruments Q2000 DSC instrument (TA Instruments, Wilmslow UK), equipped with a RCS90 Refrigerated cooling system (TA Instruments, Wilmslow UK). The instrument was calibrated against Indium and adamantane standards, and data were processed using TA Instruments Universal Analysis Software. Samples were analysed under a nitrogen atmosphere, in hermetically sealed aluminium TZero crucibles (TA Instruments, Wilmslow, UK) and subjected to three analysis cycles.

For the analysis of the uncured LCE mixtures, each cycle consisted of: a heating phase from 0–100 °C at a heating rate of 10 °C/min; an isothermal phase at 100 °C for 2 minutes; a cooling phase from 100–0 °C at 10 °C/min; and an isothermal phase at 0 °C for 2 minutes. Clearing temperatures are reported as onset values.

For LCE analysis, each cycle consisted of: a heating phase from -50–150 °C at a heating rate of 10 °C/min; an isothermal phase at 150 °C for 2 minutes; a cooling phase from 150 – -50 °C at 10 °C/min; and an isothermal phase at -50 °C for 2 minutes. The glass transition temperatures of the polymers were recorded as the onset value, on the heating phase of the second cycle.

Differential Scanning Calorimetry

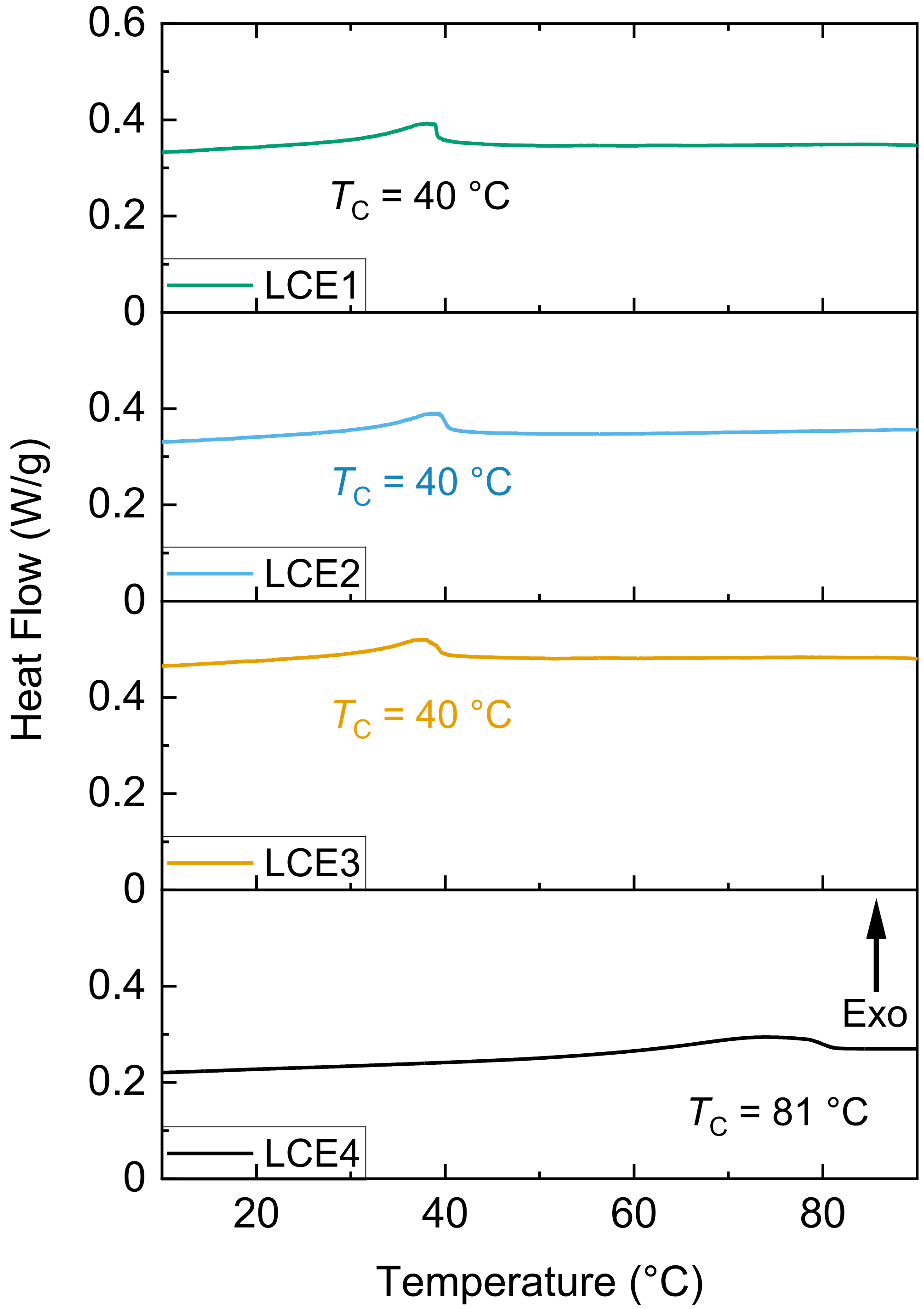

Figure S1 Precursor Mixture Differential Scanning Calorimetry *– A representative cooling cycle of the DSC thermograms for each precursor mixture.*

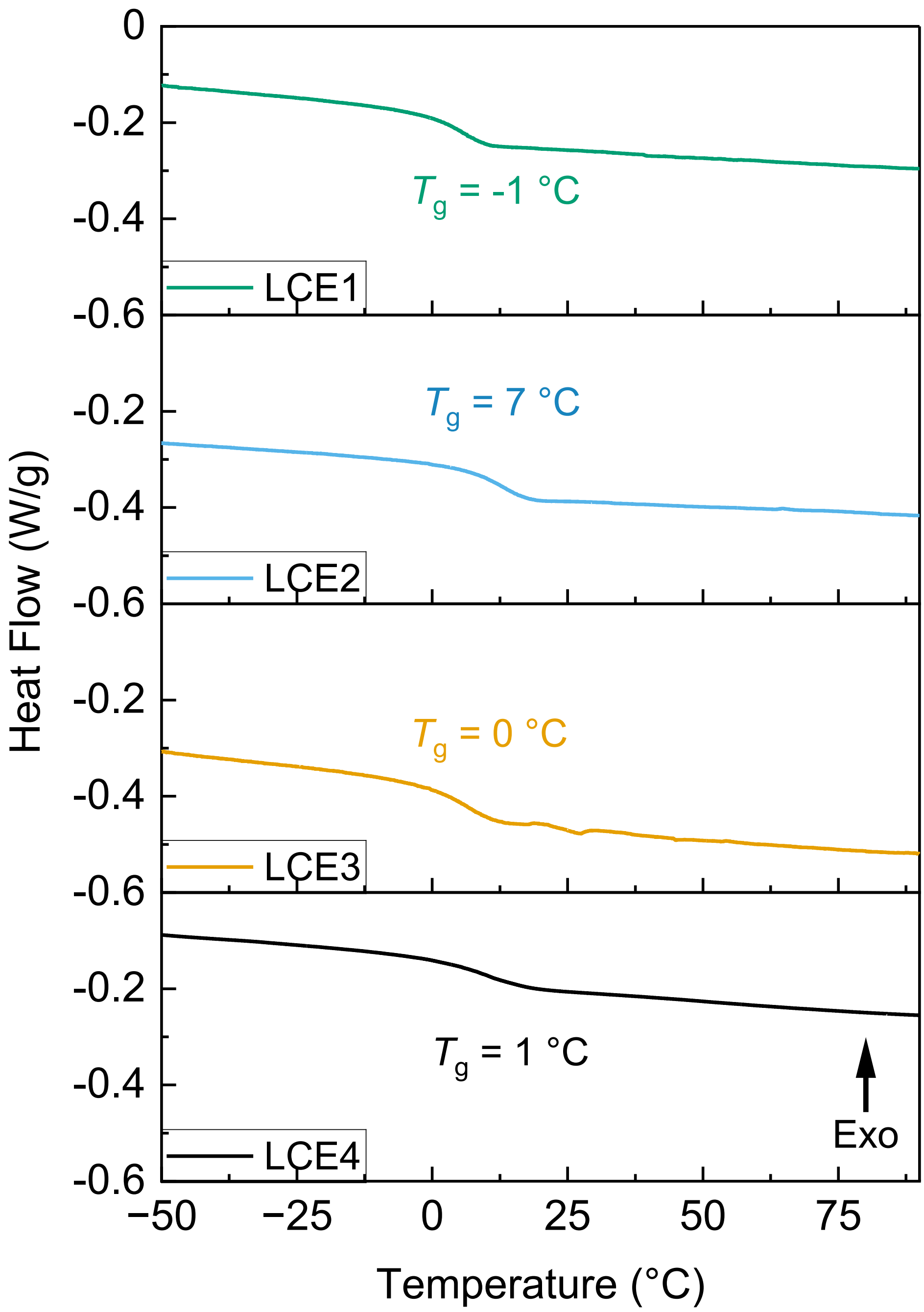

Figure S2 Cured LCE Differential Scanning Calorimetry *- A representative heating cycle of the DSC thermograms for each LCE*

Table S1 *– The glass transition temperatures (T_g) reported as onset values on heating by DSC for each LCE sample and the clearing temperature (T_c) of the precursor mixtures employed in their synthesis.*

Sample	T_g (°C)	T_c (°C)
**LCE1**	-1	40
**LCE2**	7	40
**LCE3**	0	40
**LCE4**	1	81

Polarised Light Optical Microscopy

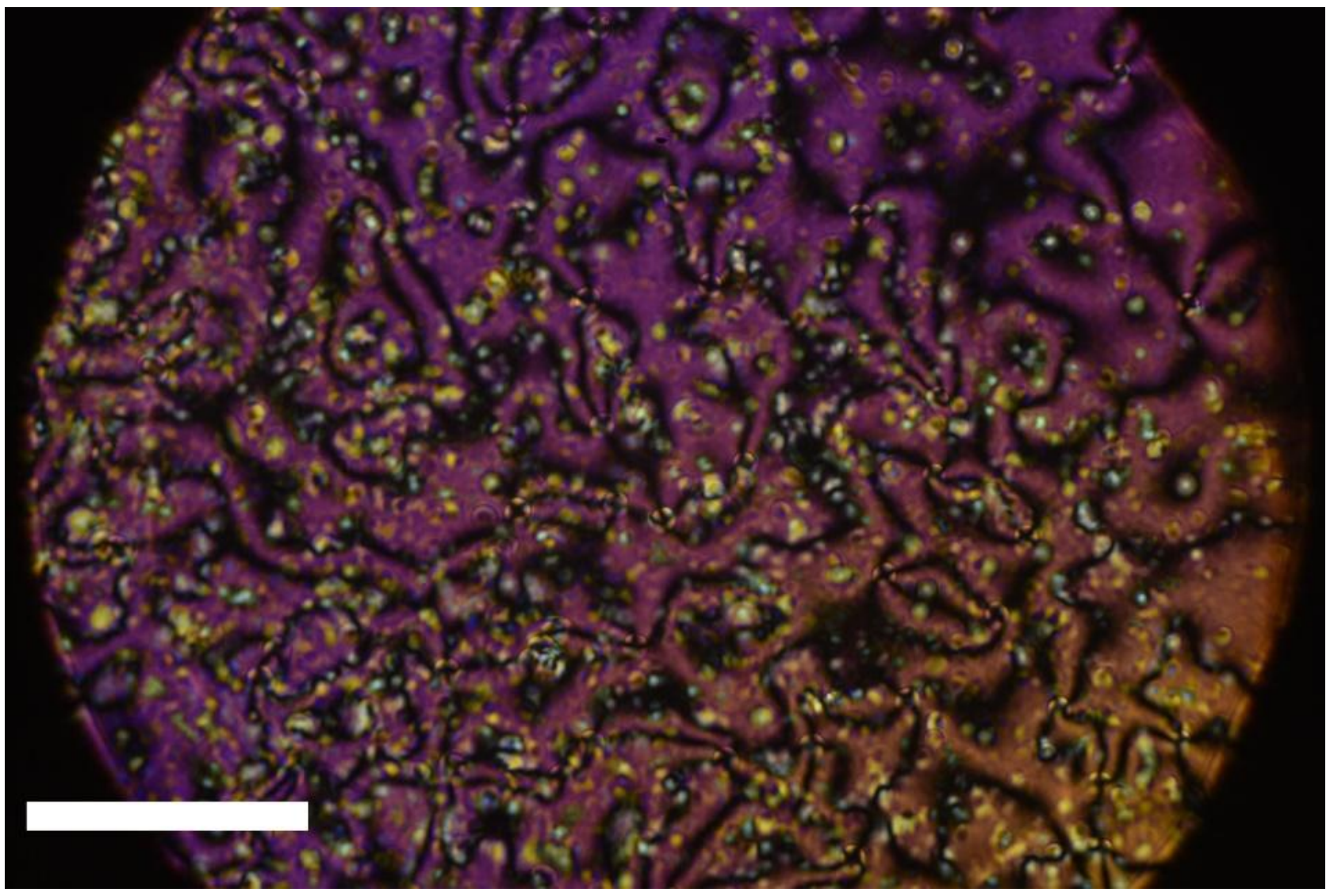

Figure S3 LCE1 Precursor Mixture Nematic Texture *– The precursor mixture used in the production of LCE1 displaying a nematic Schlieren texture when viewed under crossed polarisers when sandwiched between untreated glass substrates at room temperature. The scale bar represents 100 μm.*

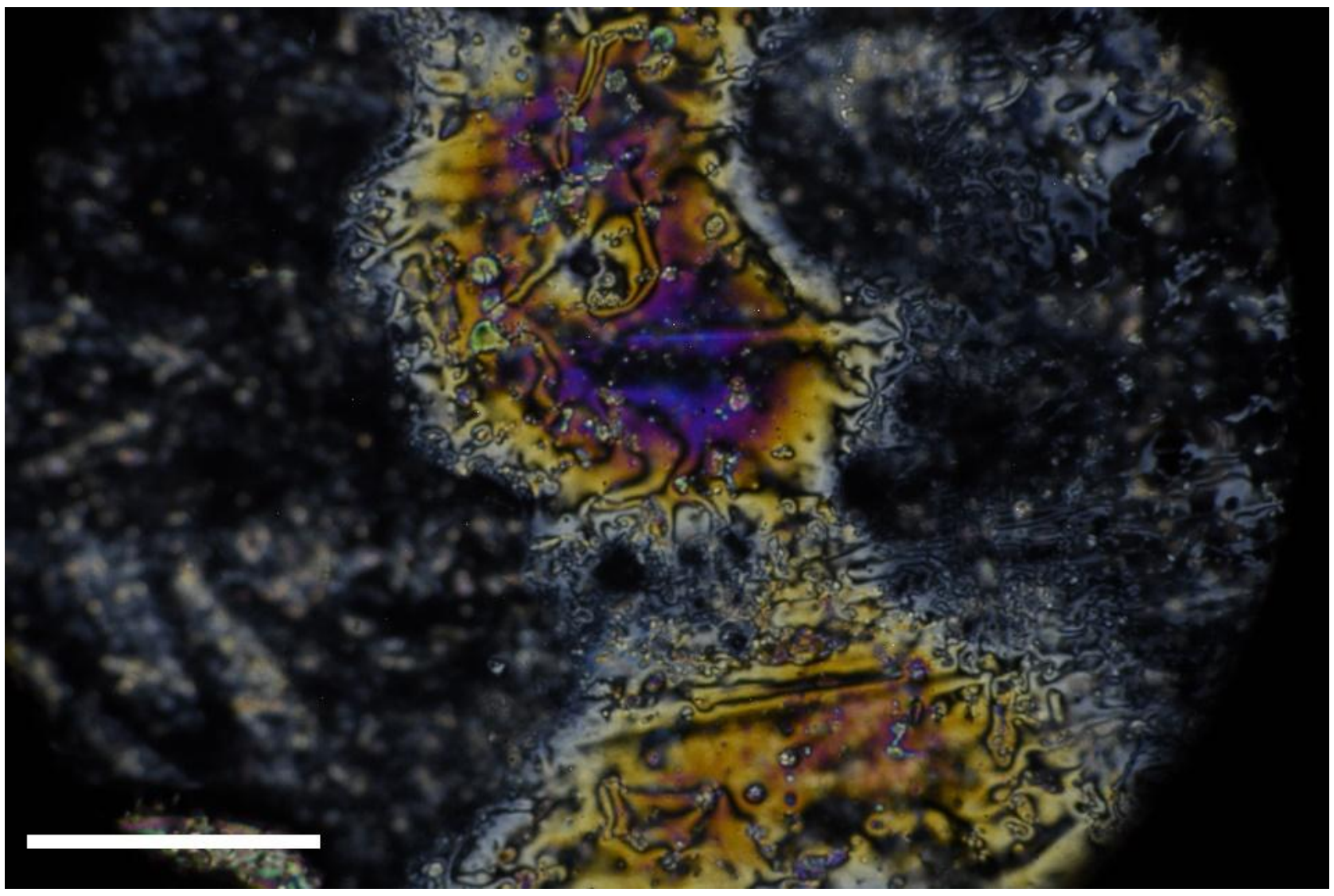

Figure S4 LCE2 Precursor Mixture Nematic Texture *- The precursor mixture used in the production of LCE2 displaying a nematic Schlieren texture when viewed under crossed polarisers when sandwiched between untreated glass substrates at room temperature. The scale bar represents 1000 μm.*

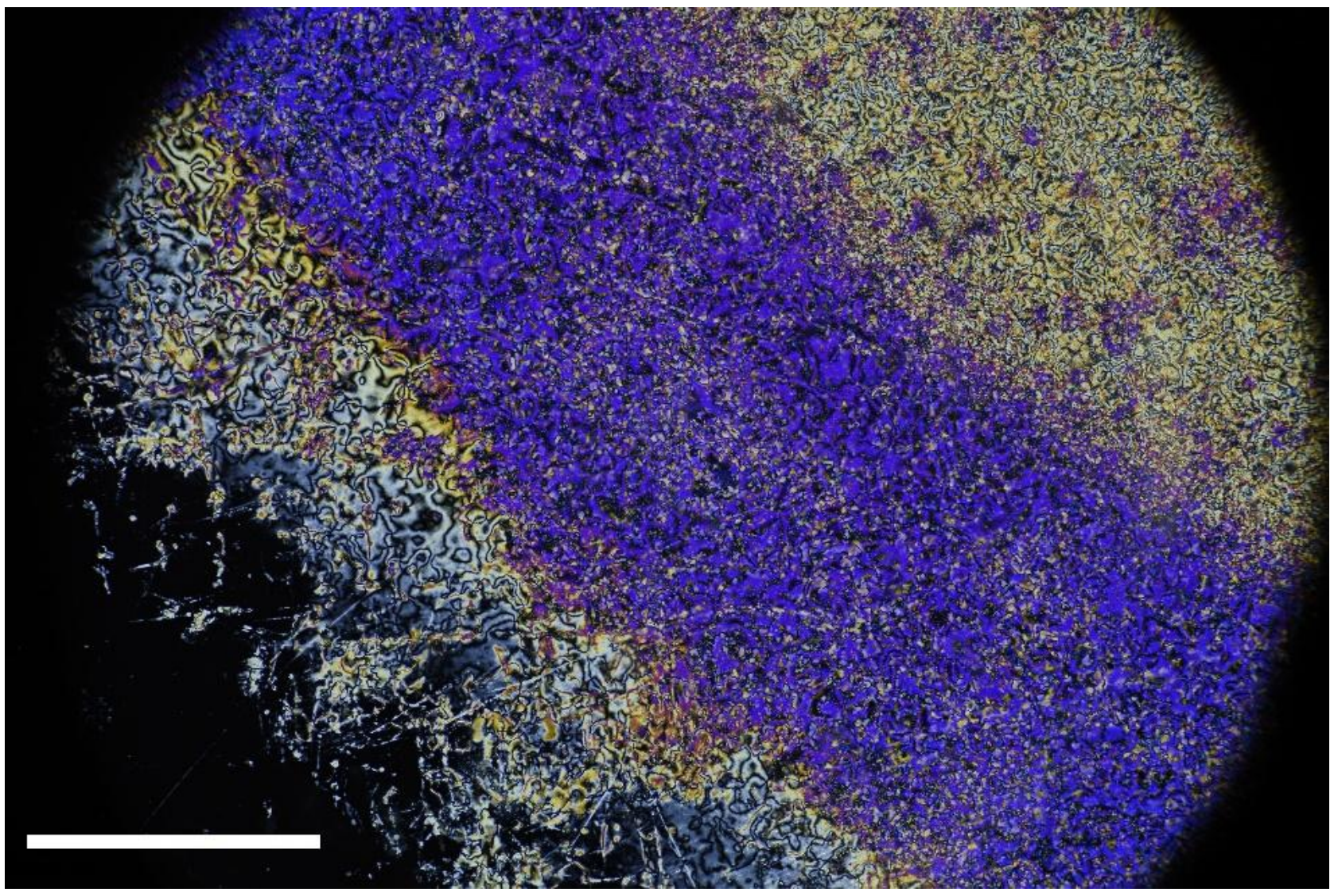

Figure S5 LCE3 Precursor Mixture Nematic Texture *- The precursor mixture used in the production of LCE3 displaying a nematic Schlieren texture when viewed under crossed polarisers when sandwiched between untreated glass substrates at room temperature. The scale bar represents 1000 µm.*

Supplementary X-Ray Scattering

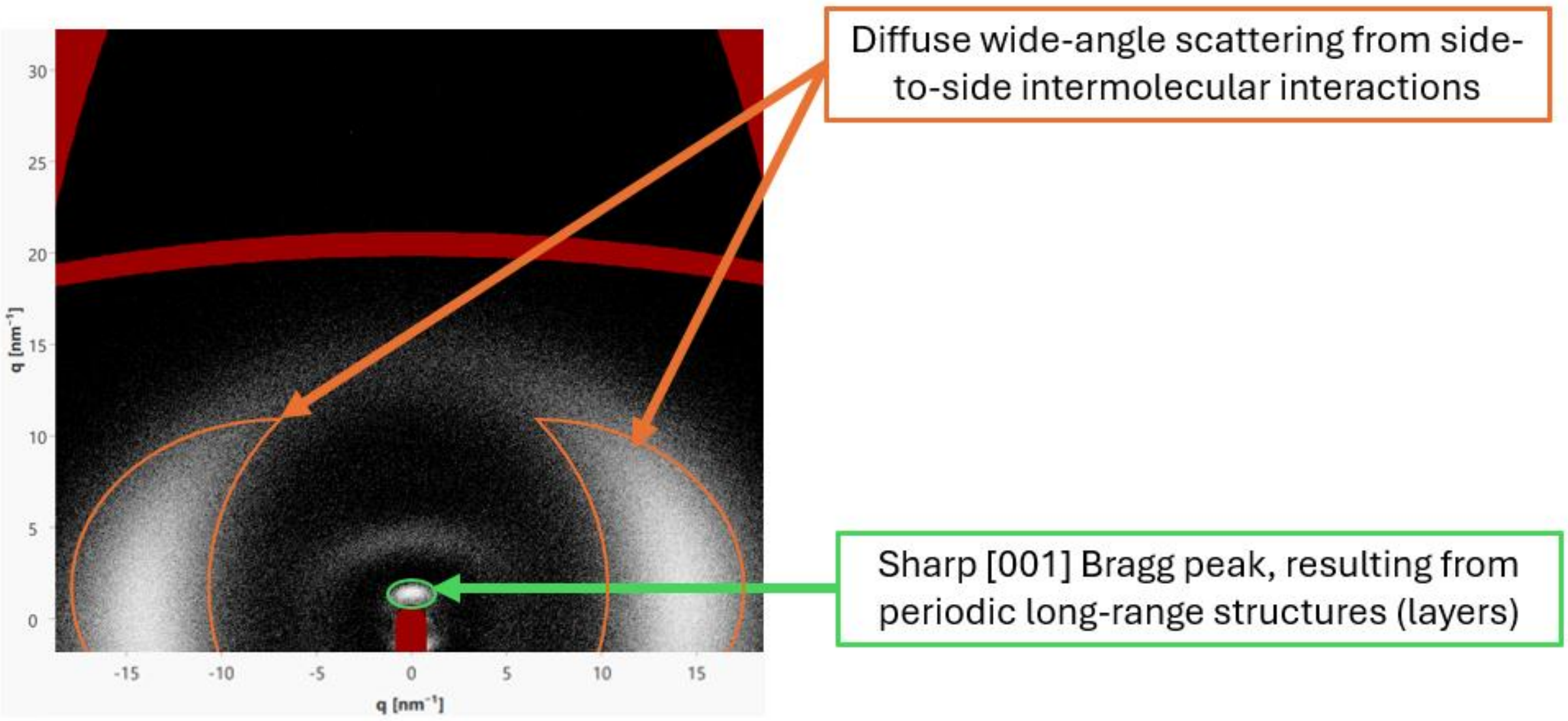

Figure S6 Important Features in X-Ray Scattering Data *– An annotated wide-angle X-ray scattering figure, highlighting important characteristics of the data and the corresponding structural features to which they are attributed.*

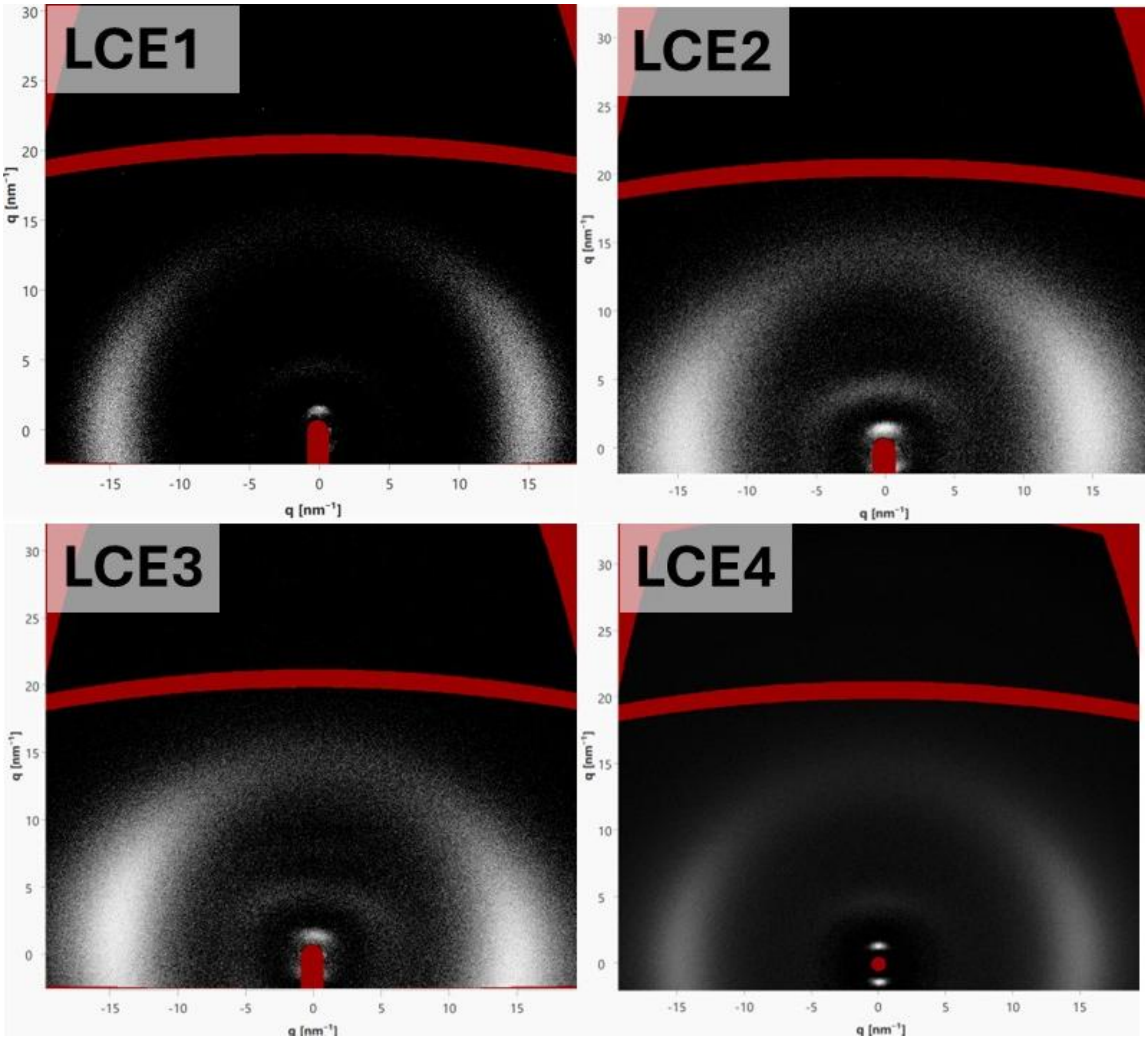

Figure S7 X-Ray Scattering Patterns for LCE1-LCE4 *– Two-dimensional wide-angle X-ray scattering data for each final LCE. The areas of the scattering patterns marked in red denote areas of the detector for which no data is recorded, due to instrument constraints. No significant features are observed in these regions.*

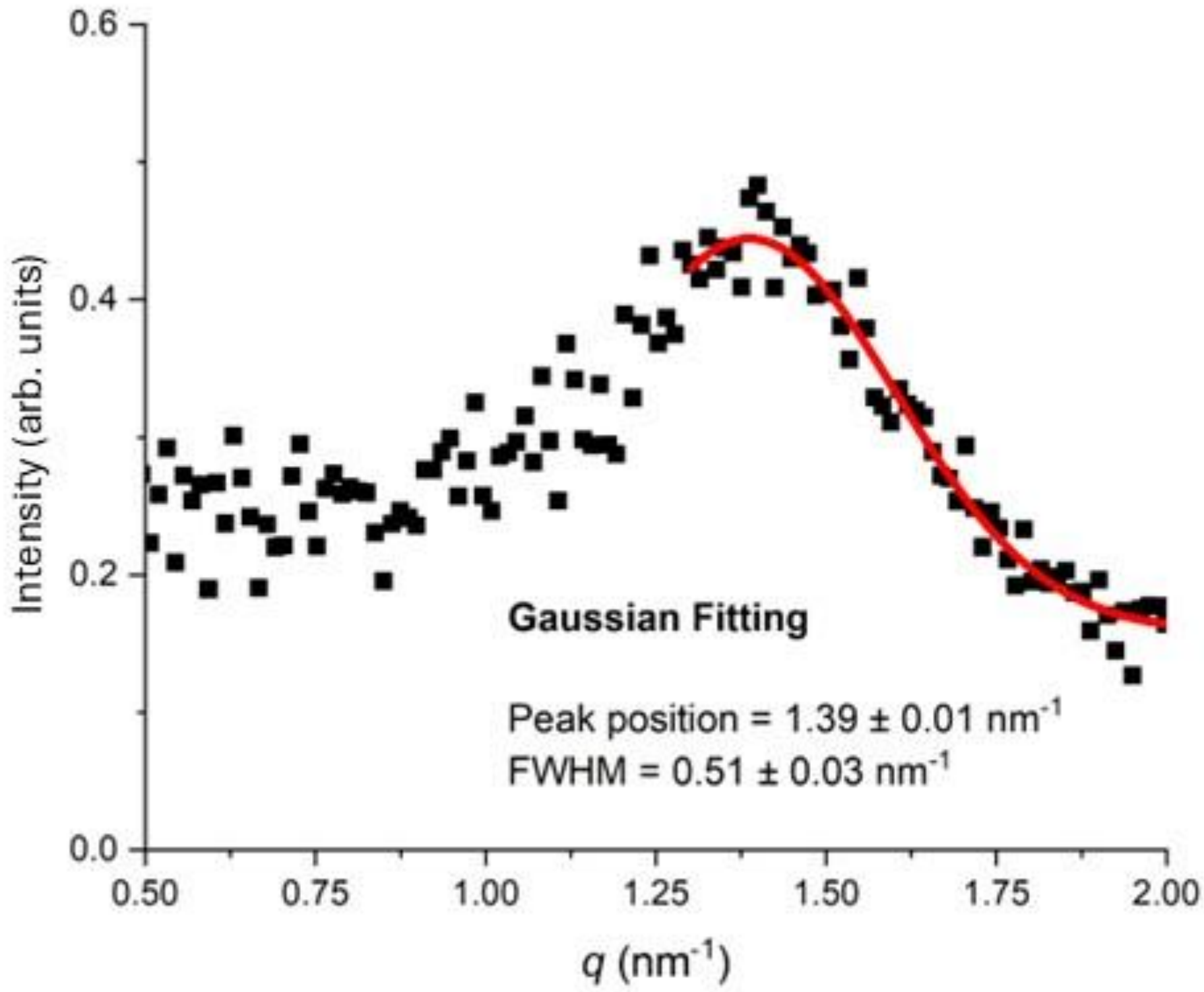

Figure S8 SAXS Fitting Example *– An example dataset showing a Gaussian fitting of the small-angle X-ray scattering diffractograms, which is used to calculate correlation lengths for the LCEs described in this work. In this case, the data shown is for a sample of LCE3.*

Polarised Raman Spectroscopy

A Renishaw inVia Raman microscope in backscattering geometry was used with a 532 nm solid-state laser of laser power 2.5 mW to determine the Raman spectra of LCE1, LCE2, and LCE4. A 50x objective was focused onto the sample. 5x 10 second accumulations were used. The LCE samples were placed inside a manual straining rig and measurements taken for each selected strain in 10 ° intervals through a full 360 ° rotation for light polarised both parallel and perpendicular to the analyzer. The depolarization ratio is the ratio of the scattered intensity polarised perpendicular to parallel, $R = \frac{I_{\perp}}{I_{\parallel}}$. The intensity is dependent on the differential polarisability ratio, r, the director angle, θ , the second-order order parameter, $\langle P_{200} \rangle$, and the fourth-order order parameter, $\langle P_{400} \rangle$ (Equations SE1 and SE2 for perpendicular and parallel respectively). The $v_{1\mathrm{g}}$ symmetric phenyl breathing mode produces a Raman peak at 1606 cm^{-1} (Figure S9) and is suitable for determining the order parameter of liquid crystals.[1]

Monitoring the angle of maximum intensity of the 1606 cm^{-1} peak allows the director orientation to be monitored under strain and during the experiment.[2]

$$I_{\perp} = \frac{1}{15}(1-r)^2 + \langle P_{200} \rangle \left[\frac{1}{21}(1-r^2)\right] + \langle P_{400} \rangle \left[\frac{1}{280}(1-r)^2(3 - 35\cos 4\,\theta)\right] \qquad \text{SE1}$$

$$I_{\parallel} = \frac{1}{5} + \frac{4r}{15} + \frac{8r^2}{15} + \langle P_{200} \rangle \left[\frac{1}{21}(3 + r - 4r^2)(1 + 3\cos 2\,\theta)\right] + \langle P_{400} \rangle \left[\frac{1}{280}(1-r)^2(9 + 20\cos 2\,\theta + 35\cos 4\,\theta)\right] \qquad \text{SE2}$$

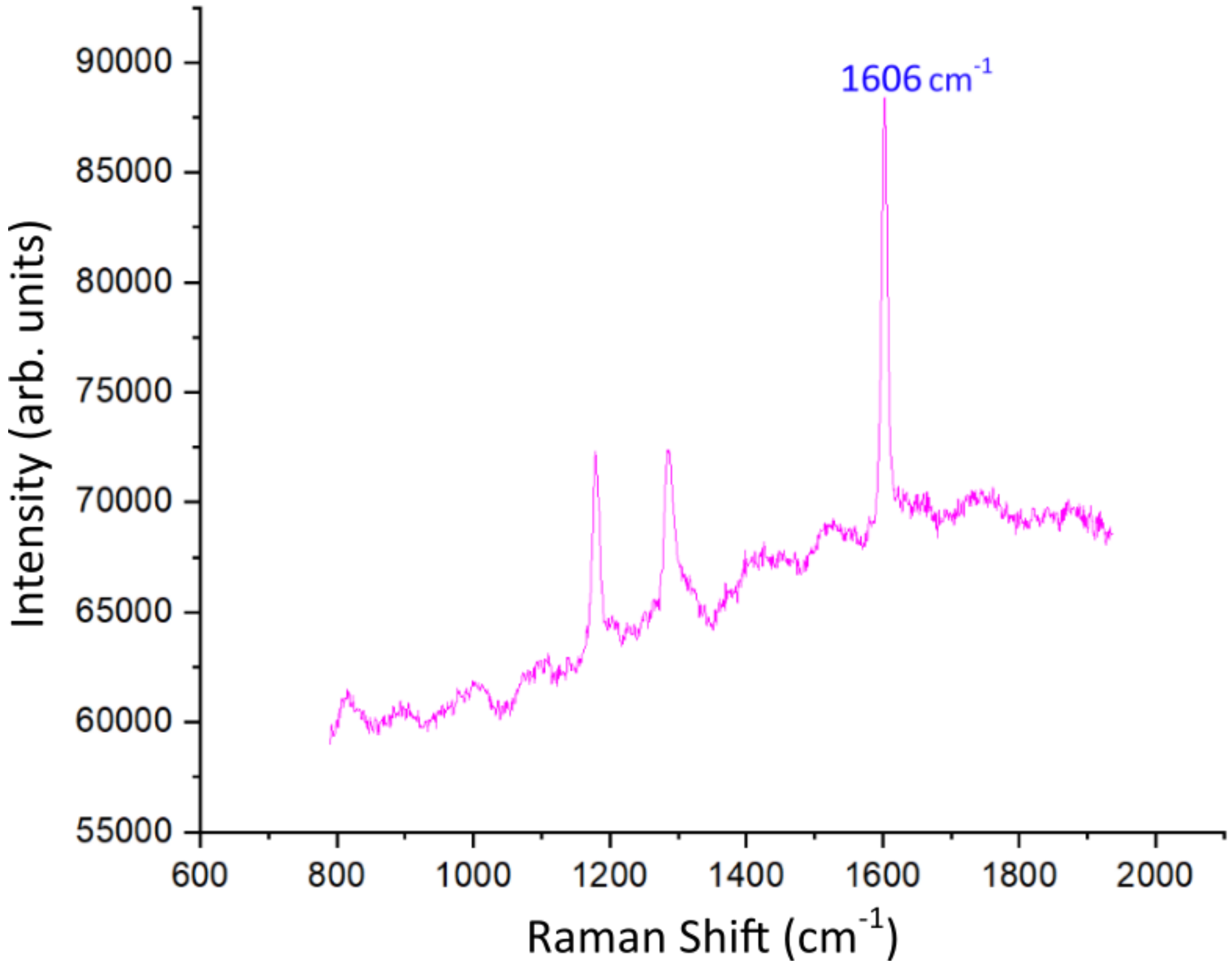

***Figure S9 Raman Spectroscopy** – A typical Raman spectrum, in this case for the unstrained LCE4. The peak at 1606 cm^{-1} corresponds to the phenyl breathing mode and the long axis of the mesogens.* The peak height is calculated from the difference to the baseline.

Smectic Layer Compressibility Modulus Calculation

The reduced smectic layer compressibility modulus, b, is shown to impact the free energy of a smectic LCE under an imposed deformation, as such it is useful to calculate the value of b for a given smectic LCE. To estimate a value of b, stress-strain measurements of a planar LCE strained perpendicular to the initial director are used where the strain is defined along the *x*-axis and the director along the *z*-axis. Minimization of the free energy of such an imposed deformation yields the equation[3]:

$$\lambda_{xx}^2 \lambda_{zz}^2 - 1 = b(1 - \lambda_{xx}\lambda_{zz}). \qquad \text{SE3}$$

By measuring the deformation in each principal direction, a value of b can be deduced by a least-squares regression to the above equation. This method has previously been used by Berrow *et al.* To calculate the values of b for LCE1, LCE2, and LCE3[4].

Supplementary References

1. Zhang, Z. & Gleeson, H. F. Understanding liquid crystal order parameters deduced from different vibrations in polarised Raman spectroscopy. *Liq. Cryst.* **46**, 219–233 (2019).
2. Raistrick, T., Zhang, Z., Mistry, D., Mattsson, J. & Gleeson, H. F. Understanding the physics of the auxetic response in a liquid crystal elastomer. *Phys. Rev. Res.* **3**, 023191 (2021).
3. Adams, J. M. & Warner, M. Elasticity of smectic-A elastomers. *Phys. Rev. E Stat. Nonlin. Soft Matter Phys.* **71**, 021708 (2005).
4. Berrow, S. R. *et al.* The effect of templating on the mechanical properties of smectic liquid crystal elastomers. *J. Mater. Chem. C Mater.* **13**, 12365–12375 (2025).
