# Supplementary material for: Frustrated smectic liquid crystal elastomers as multifunctional mechanical metamaterials
Source: Nat Commun. 2026 Sep 11;17:10726. doi: 10.1038/s41467-026-76776-x (PMC13658772; doi:10.1038/s41467-026-76776-x)
Supplement: Supplementary file 2 [file 41467_2026_76776_MOESM2_ESM.pdf]

nature portfolio

Peer Review File

Frustrated Smectic Liquid Crystal Elastomers as Multifunctional Mechanical Metamaterials

Corresponding Author: Dr Stuart Berrow

This file contains all reviewer reports in order by version, followed by all author rebuttals in order by version.

Version 0:

Reviewer comments:

Reviewer #1

(Remarks to the Author)
To the authors,

The paper by Street et al. presents a method for producing solid auxetic materials using a new class of frustrated smectic-A liquid crystal elastomers (LCEs). This work extends their previous studies on auxetic nematic LCEs. The authors employ essentially the same chemical formulation and mesogenic units, modified with slightly longer alkyl tails to stabilize smectic rather than nematic order. The resulting smectic-A LCEs display negative Poisson's ratios at strains above ~0.4, representing the first reported observation of auxetic behaviour in smectic LCEs.
In my view, this study is important and offers significant potential for designing new classes of smart adhesive and impact-damping materials. I therefore recommend publication in Nature Communications after the authors address the following comments.

Major reviews.

1- The molecular auxetic behaviour in nematic LCEs arises from reorientation of the nematic director under tensile strain. By contrast, smectic A LCEs possess strongly layered intermolecular structures that restrict molecular reorientation and introduce additional deformation modes such as layer undulation, dilation, or slip. These structural differences would be expected to influence both the onset and magnitude of auxetic behaviour. Can the authors comment on the rate dependent response in smectic A versus nematic LCEs? The manuscript would benefit from clarification on whether the observed auxetic response is strain rate dependent. In nematic LCEs, director rotation is known to have a finite relaxation time and can therefore exhibit viscoelastic and time dependent behaviour. It would be helpful for the authors to discuss whether similar or distinct rate effects arise in smectic A LCEs, given their additional layered constraints.

2- On page 10, line 189, the phrase "mechanmechanical behaviour" appears to be a typographical error. Could the authors clarify what specific mechanical behaviour they are referring to in this section?

3- Page 10, line 181 — Please clarify how Tg was determined? You report Tg values of 7 °C, 0 °C, and 1 °C for LCE2, LCE3, and LCE4, respectively. However, the tan δ peak in the DMA traces (typically associated with Tg) appears at higher temperatures. Could the authors explicitly explain how Tg was determined?

4- In Figure 4, the DMA data appear to be collected over a limited temperature range. To properly characterise the full thermomechanical behaviour of these LCEs, I strongly suggest extending the temperature sweep. A range from –50 °C to +100 °C would allow the authors to capture: the full glassy regime, the glass transition, any sub Tg β relaxations, the rubbery plateau, and any high temperature softening associated with mesophase transitions.

5- The statement (see page 13, line 247)"The loss factor, tan δ, shows a single peak that is associated with the glass transition at ~0 °C in both materials" is not consistent with the data. From the plotted curves, the tan δ peak appears substantially above 0 °C (≈ 10–15 °C). Please revise this statement to reflect the actual peak position. To avoid ambiguity, I recommend the authors to increases sweep range: the temperature sweep range appears too narrow to locate the loss peak unambiguously. Starting the DMA sweep at ~–50 °C and extending to at least +100 °C would better capture the glassy regime, and the rubbery plateau, thereby allowing a clear and reproducible identification of the peak position.

6- Using Equation 7 to calculate the adhesion factor by inserting the measured values of tan δ and G″ is useful; however, it may not necessarily correlate with actual adhesion performance. I believe this paper would be significantly improved if the authors conducted a simple adhesion test to directly determine adhesion as a function of temperature. Such an adhesion test could include a tack-force measurement or a peel-force test.

Reviewer #2

(Remarks to the Author)
This study reports for the first time the auxetic behavior of frustrated smectic-A liquid crystalline elastomers (SmA LCEs), expanding the family of molecular auxetic materials while uncovering their anisotropic shear modulus and adhesive properties. The research holds significant scientific value and application potential, and the overall work is well-founded for submission to Nature Communications. However, there remain aspects requiring further refinement and supplementation in terms of innovation, mechanistic elucidation, data completeness and research systematicity. The specific review comments are as follows:
1. This paper reports a novel auxetic liquid crystalline elastomer (LCE) based on the frustrated smectic-A (SmA) phase, which exhibits notable innovation in the field of auxetic materials. However, the discussion section lacks a sufficient comparison of its core advantages over conventional nematic auxetic elastomers. It is recommended to systematically elucidate the specific advantages and potential application scenarios of frustrated SmA-phase LCEs relative to nematic LCEs, so as to highlight the scientific value and application potential of this work.
2. The paper states that the threshold strains for the Poisson's ratio of LCE2, LCE3 and LCE4 are 0.41±0.05, 0.50±0.05 and 1.05±0.05, respectively. However, it fails to identify the core factors responsible for the differences in threshold strain among the various samples, merely mentioning that there is no obvious correlation with the glass transition temperature. It is recommended to conduct an in-depth analysis of the causes for such differences in threshold strain by combining relevant structural parameters of the samples, such as crosslink density and smectic correlation length.
3. Although Figure 3 shows the effect of strain on the thickness and width of the samples, it lacks characterization results of the micromorphology before and after the strain exceeds the threshold, making it difficult to directly reflect the structural evolution process. Supplementary characterization of the actual state of the samples at this stage is suggested, including molecular orientation and smectic layer structural changes at the microscale, as well as sample morphology and transparency at the macroscale, to provide direct experimental evidence for the intrinsic mechanism of thickness variation.
4. Using this method, how large of the film can be prepared?
5. Errors exist in the ordinate labels of Figure 4d and Figure 4h. The authors are suggested to carefully check the axis labels and physical units of all figures and tables throughout the manuscript to avoid similar labeling errors and ensure the accuracy and readability of data presentation.

Reviewer #3

(Remarks to the Author)
The manuscript reports the synthesis, characterization and analysis of frustrated smectic liquid crystal elastomers which show negative Poisson's ratio beyond a threshold strain and anisotropic adhesion. The study reports a new class of liquid crystal elastomers with auxetic behaviors (negative Poisson's ratio) beyond previously reported nematic liquid crystal elastomers (LCEs). While there have been many studies reporting auxetic behaviors at structure level by designing "metamaterials", materials with auxetic behaviors at atomic or molecular level are rare. Thus, there could be potential new opportunities from the findings which can be of interest to the readership of Nature Communications. The manuscript would be suitable for publication after addressing the following questions, comments, and suggestions.

1. The title of the manuscript is "Frustrated Smectic Liquid Crystal Elastomers as Multifunctional Mechanical Metamaterials". However, there seem to be no supporting data regarding multifunctional aspects of the materials. It will be helpful to add relevant information.

2. It will be helpful to strengthen the introduction and the discussion part regarding the advantages of the frustrated smectic liquid crystal elastomers compared with nematic liquid crystal elastomers so that readers can appreciate the potential importance of the findings.

3. In the introduction, it was mentioned that molecular auxetic materials could open new opportunities, but there was no data or demonstration in this paper that show clear evidence. It will be helpful and critical to add data that show unique potential of molecular auxetic materials that no other "micro/macro-structural" auxetic materials can have.

4. Furthermore, it will be helpful to have examples where frustrated smectic liquid crystal elastomers show superior properties. In the introduction, it was discussed that auxetic materials can offer mechanical advantages such as resistance to impact, delamination and tear. However, there is no data comparing performance of the reported material with data from other materials including other liquid crystal elastomers. So, having such quantitative comparisons will provide useful guidelines to potential users.

5. Here are some minor suggestions/questions/comments to improve the clarity for readers:

1) Lines 13, 31: be specific about the type of strain
2) Line 46: discuss what are the factors that determine the auxetic strain-threshold
3) Line 63: check the typo
4) Line 64: check the font
5) Figure 1: it will be helpful to have a diagram visualizing geometric frustration of the smectic LCEs that the authors report so that readers can have a better understanding of the underlying mechanism and the molecular structure of the material.
6) Line 168: check whether figures are labeled correctly.
7) Eq. 1: define mu, T, r, and b
8) Lines 214-218: keep the font consistent with other parts
9) Line 236: How do we know the layer spacing modulus? from the slope of the plot?
10) Lines 377, 380: Stress-strain?

Version 1:

Reviewer comments:

Reviewer #1

(Remarks to the Author)

I have reviewed the revised manuscript and the authors' responses. All my previous concerns have been satisfactorily addressed. The manuscript now presents a clear and convincing study of auxetic behaviour in smectic liquid crystal elastomers and will be of broad interest. I have no further comments and recommend publication in Nature Communications.

Reviewer #2

(Remarks to the Author)
The authors have well addressed all my concerns. I think its current version can be accepted.

Reviewer #3

(Remarks to the Author)
The authors addressed the comments/suggestions/questions by the reviewer.
I recommend the publication of the manuscript as it is.
Congratulations!

Open Access This Peer Review File is licensed under a Creative Commons Attribution 4.0 International License, which permits use, sharing, adaptation, distribution and reproduction in any medium or format, as long as you give appropriate

credit to the original author(s) and the source, provide a link to the Creative Commons license, and indicate if changes were made.
In cases where reviewers are anonymous, credit should be given to 'Anonymous Referee' and the source.
The images or other third party material in this Peer Review File are included in the article’s Creative Commons license, unless indicated otherwise in a credit line to the material. If material is not included in the article’s Creative Commons license and your intended use is not permitted by statutory regulation or exceeds the permitted use, you will need to obtain permission directly from the copyright holder.
To view a copy of this license, visit https://creativecommons.org/licenses/by/4.0/

Dr Stuart R. Berrow
School of Physics and Astronomy
University of Leeds
Leeds, LS2 9JT
S.R.Berrow@leeds.ac.uk
Friday 19th June 2026

Dear referees,

Firstly, we would like to thank you for your time in considering, our manuscript titled 'Frustrated Smectic Liquid Crystal Elastomers as Multifunctional Mechanical Metamaterials' submitted for consideration in *Nature Communications* for the Nature portfolio collections on 'Advances in Liquid Crystal Elastomers'. Following your comments, we have revised the manuscript, which is provided with the changes highlighted in yellow, to aid their identification.

Below we provide a point-by-point description as to how your comments have been addressed in the revised manuscript. Your comments are provided in black text, and our response provided in blue text, with excerpts copied from the manuscript text then being highlighted in yellow for clarity.

We hope that you agree that our manuscript addresses the comments, and is now suitable for publication in *Nature Communications*.

Reviewer One

To the authors,

The paper by Street et al. presents a method for producing solid auxetic materials using a new class of frustrated smectic-A liquid crystal elastomers (LCEs). This work extends their previous studies on auxetic nematic LCEs. The authors employ essentially the same chemical formulation and mesogenic units, modified with slightly longer alkyl tails to stabilize smectic rather than nematic order. The resulting smectic-A LCEs display negative Poisson's ratios at strains above ~0.4, representing the first reported observation of auxetic behaviour in smectic LCEs.
In my view, this study is important and offers significant potential for designing new classes of smart adhesive and impact-damping materials. I therefore recommend publication in Nature Communications after the authors address the following comments.

We thank reviewer one for their positive and constructive assessment of our manuscript, and for the suggestions given as to how to strengthen the manuscript. Below we provide a point-by-point discussion as to how we have addressed the suggested revisions.

Major reviews.

1- The molecular auxetic behaviour in nematic LCEs arises from reorientation of the nematic director under tensile strain. By contrast, smectic A LCEs possess strongly layered intermolecular structures that restrict molecular reorientation and introduce additional deformation modes such as layer undulation, dilation, or slip. These structural differences would be expected to influence both the onset and magnitude of auxetic behaviour. Can the authors comment on the rate dependent response in smectic A versus nematic LCEs? The manuscript would benefit from clarification on

whether the observed auxetic response is strain rate dependent. In nematic LCEs, director rotation is known to have a finite relaxation time and can therefore exhibit viscoelastic and time dependent behaviour. It would be helpful for the authors to discuss whether similar or distinct rate effects arise in smectic A LCEs, given their additional layered constraints.

We agree completely that the geometric constraints imposed by the layered structures of smectic A LCEs would be expected to influence the auxetic behaviour in these samples, in addition to factors such as strain-rate dependence. We have included some additional data obtained at different strain rates (half, double and four times the original strain rate) on samples of LCE2 to explore such phenomena. For the convenience of the reviewer, below we provide the updated data and a copy of our revised discussion:

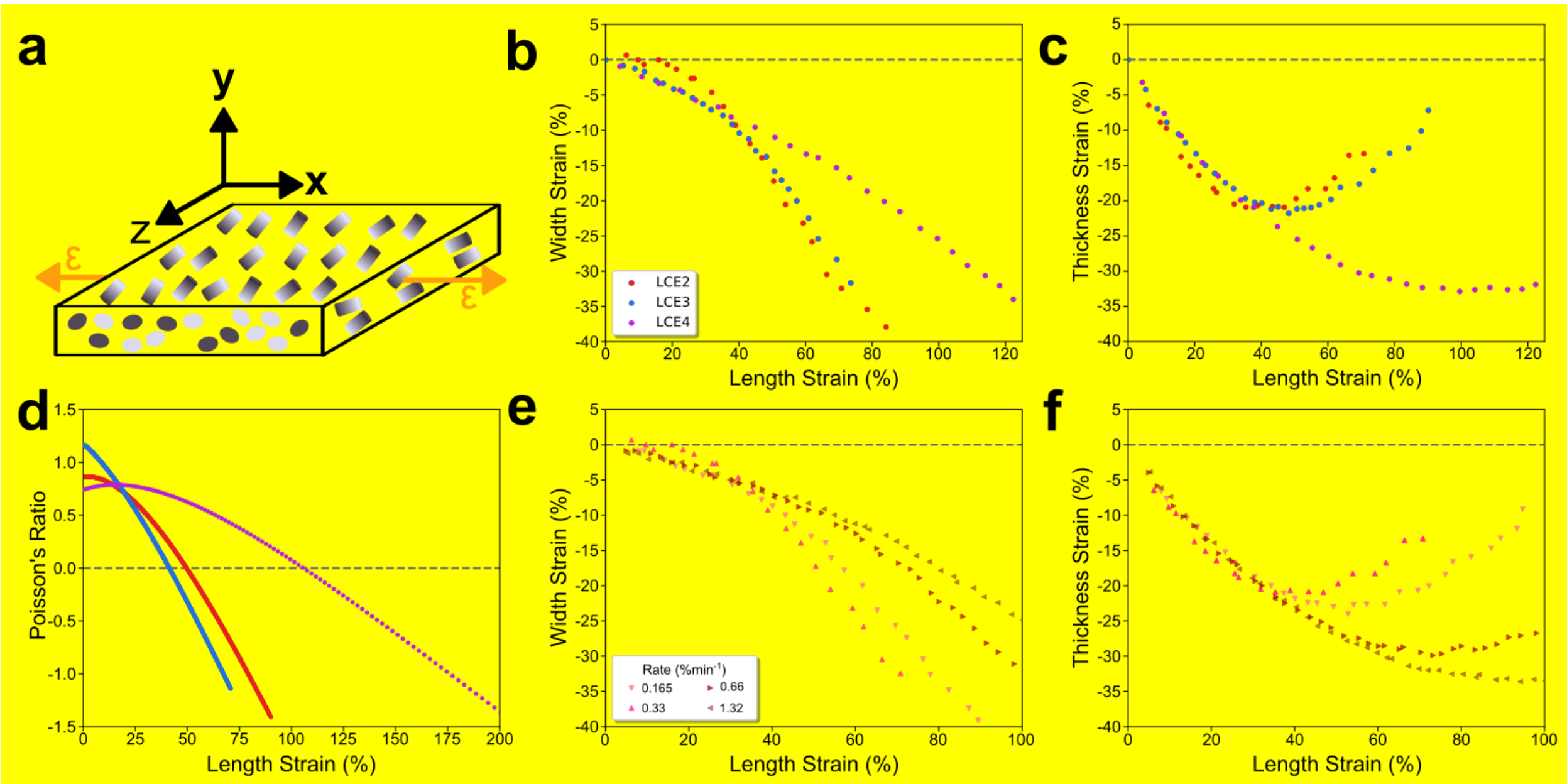

Figure 3. (a) A schematic representation of the experimental conditions employed in the analysis of auxeticity in the LCE samples, defining the naming convention of the x (length), y (thickness) and z (width) axes, where ε represents the applied strain and the rods depict the alignment of the mesogens in the LCE. Panels (b) and (c) show the effect of an applied length (x) strain on the sample dimensions in the width (z) and thickness (y) dimensions respectively for each sample when strained at a rate of 0.33%/min at room temperature. An increase in sample thickness occurs above threshold length strains of 41±5%, 50±5% and 105±5% for LCE2, LCE3 and LCE4 respectively. LCE1 consistently failed at strains of ~30%. d) Shows the instantaneous Poisson's ratio in the y-axis (v) deduced for each sample as a function of the applied x-strain. e) and f) Show the measured strain (in width and length respectively) for LCE2 strained at different rates: 0.165%/min (down triangles), 0.33%/min (upright triangles), 0.66%/min (right-facing triangles), and 1.32%/min (left-facing triangles). The auxetic thresholds become 53±5%, 47±5%, and 73±5% for 0.5x, 1x, and 2x respectively, an auxetic threshold is not observed at a rate of 4x up to a strain of 100% although the initial strain behavior is similar.

'For auxetic nematic LCEs, it has previously been observed that the auxetic threshold strain is dependent on several factors, most notably dynamics, for example the temperature or the rate at which strain is applied to the sample.[8,33] Given that the layered structure present in smectic LCEs adds more geometric considerations than in nematic LCEs, we were interested to undertake some initial studies to explore whether the strain rate similarly impacts the auxetic response in these smectic LCE systems. In these experiments, samples of LCE2 were examined at different strain rates, namely 0.165%/min, 0.33%/min, 0.66%/min and 1.32%/min, and the data are shown in Figure 3e and f.

In nematic auxetic LCEs, it has been reported that as the strain rate is increased, the auxetic threshold of the nematic LCE increases, as faster strain rates can be thermorheologically related to lower temperatures.[33] The data reported in Figure 3e and f suggest that in the frustrated smectic LCEs there is a similar strain-rate dependence. Taking the rate of 0.33%/min as the base strain rate as this is the rate used to compare samples of different composition (Figure 3b, c and d), it is observed that as the strain rate is increased (doubled and quadrupled to 0.66%/min and 1.32%/min respectively), the auxetic threshold also increases. This increase is such that for the sample strained at 1.32%/min, no auxetic response is observed prior to sample failure. Interestingly, we find that if the strain rate is halved from the original 0.33%/min to 0.165%/min, the auxetic threshold remains comparable. One may expect a decrease in the auxetic threshold based on this change in strain rate, so this result is somewhat surprising. This finding may result from a constraint imposed by the layered structure present in the frustrated smectic sample, or it may simply be that the change in strain rate imposed was insufficient to substantially change the dynamics of the system. These initial findings suggest that a more complex relationship potentially exists between strain rate and auxetic threshold in these frustrated smectic systems than in the nematic case. A future, more detailed study considering factors such as strain rate and temperature will be conducted to deduce this relationship.

Furthermore, it has been previously reported that the auxetic response in nematic LCEs appears to be governed by proximity to the glass transition temperature (Tg) of the sample, with a higher Tg leading to higher auxetic thresholds.[8,33] For the smectic auxetic LCEs reported here, there is no facile relationship between Tg and the auxetic threshold; LCE2, LCE3 and LCE4 have a Tg of 7 °C, 0 °C and 1 °C by differential scanning calorimetry respectively (Table S1), yet the auxetic thresholds follow the order LCE3 < LCE2 < LCE4, indicating a non-monotonic relationship. These results therefore suggest that the auxetic response in smectic LCEs may be influenced by more characteristics than those known to influence the auxetic response in nematic LCEs. Attempts to elucidate these characteristics will be the subject of future studies.'

A full exploration of the properties and dynamics of frustrated smectic auxetic LCEs is beyond the scope of this report. Understanding full details of the nematic systems required a combined effort of 8 years of research, 14 journal articles and 4 patents, and a similarly large undertaking would be required to fully explore these smectic systems, given their increased complexity. Therefore, we have included preliminary measurements of the dynamics, offering some of the additional details requested by the reviewer, but more detailed investigations must be the subject of future work.

2- On page 10, line 189, the phrase "mechanmechanical behaviour" appears to be a typographical error. Could the authors clarify what specific mechanical behaviour they are referring to in this section?

We thank reviewer one for identifying the typographical error, this has been corrected in the revised manuscript. The mechanical behaviour in this section refers to the Shear Moduli of the LCEs, as detailed in the Subheading on line 271 (page 13) in the revised manuscript.

3- Page 10, line 181 — Please clarify how Tg was determined? You report Tg values of 7 °C, 0 °C, and 1 °C for LCE2, LCE3, and LCE4, respectively. However, the tan δ peak in the DMA traces (typically associated with Tg) appears at higher temperatures. Could the authors explicitly explain how Tg was

determined?

We have clarified the manuscript, explaining that the Tg values of 7 °C, 0 °C and 1 °C are values determined from differential scanning calorimetry, and are taken as onset values on heating, in accordance with the methodology previously adopted for nematic auxetic LCEs (for example, DOI: 10.3390/polym16141957). We have also acknowledged that Tg determined via DSC doesn't necessarily coincide with the peak of tan delta from DMA, though the two are associated with the same physical process. We have copied the revised parts of the manuscript below for the reviewers' convenience:

'For the smectic auxetic LCEs reported here, there is no facile relationship between Tg and the auxetic threshold; LCE2, LCE3 and LCE4 have a Tg of 7 °C, 0 °C and 1 °C by differential scanning calorimetry respectively (Table S1), yet the auxetic thresholds follow the order LCE3 < LCE2 < LCE4, indicating a non-monotonic relationship.'

'There is a single peak in tanδ which occurs at 12 ℃ in LCE1 and 15 ℃ in LCE3 that is associated with the glass transition.'

4- In Figure 4, the DMA data appear to be collected over a limited temperature range. To properly characterise the full thermomechanical behaviour of these LCEs, I strongly suggest extending the temperature sweep. A range from –50 °C to +100 °C would allow the authors to capture: the full glassy regime, the glass transition, any sub Tg β relaxations, the rubbery plateau, and any high temperature softening associated with mesophase transitions.

We thank reviewer one for their insight into the DMA data, and have now increased the temperature range of the DMA data, with experiments now encompassing the range of -15 to +100 °C which is well below all glass transition temperatures. We did not explore lower temperatures as previous broadband dielectric spectroscopic experiments on a similar (nematic) LCE revealed β relaxations at very low temperatures, -83 °C (DOI: 10.3390/molecules26237313). Consequently, any information about the β relaxations in this system are unlikely to be revealed, even if we went explored temperatures as low as the instrument minimum (–100 °C).

Whilst we agree it would be ideal to be able to collect data for temperatures as low as -50 °C, below the -15°C to which our current data is collected, the samples are sufficiently glassy that sample slippage becomes problematic, leading to large quantities of noise in the data. Furthermore, we believe that with the newly extended data series we have provided (Figure 5 in the revised manuscript, and copied below for convenience), we fully capture interesting phenomena, seeing a clear plateau in the storage modulus, as well as the full breadth of the tan(δ) peak.

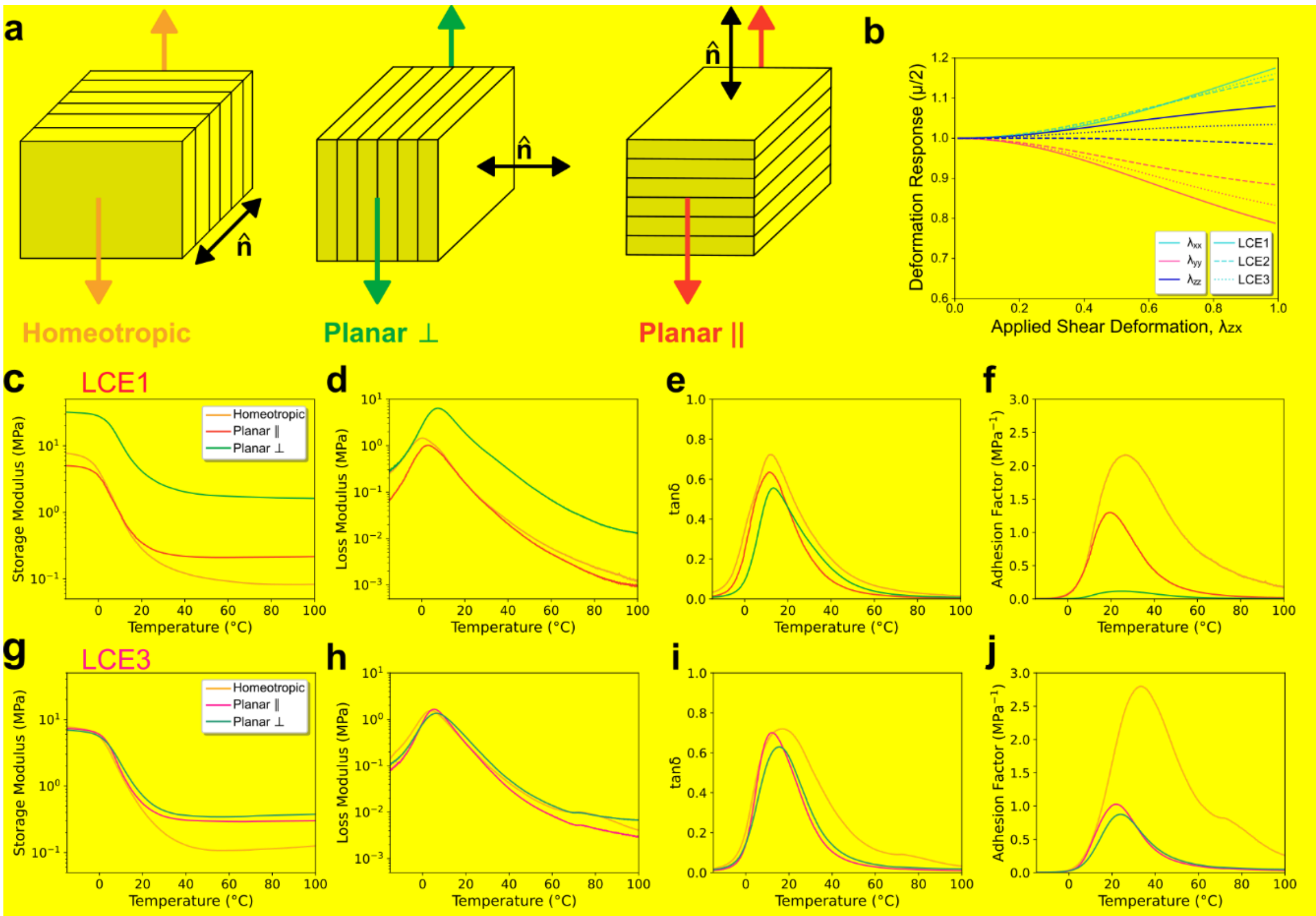

Figure 5. (a) All possible geometries showing the relationship between the smectic layers and applied shear (arrows), with homeotropic (orange), planar ⊥ (green), and planar ∥ (red). (b) For the planar | | case, the applied shear deformation is, λ_{zx} and the minimised principal deformations in λ_{xx} (turquoise), λ_{yy} (pink), and λ_{zz} (blue). The calculation is for a step length anisotropy r = 2.1 and smectic layer compression moduli b = 7.5 (solid), b=3.4 (dotted), and b=5.2 (dashed) which represent LCE1, LCE2, and LCE3 respectively.[29] (c) to (e) and (g) to (i) show the storage modulus G', the loss modulus G'', and dissipation tanδ = G''/G', of LCE1 and LCE3 respectively, determined through shear dynamic mechanical analysis at 1 rads^{-1}. (f) and (j) present an adhesion factor A = tanδ/G' determined from the shear DMA data. For c) to j), the different film alignments are identified through homeotropic (orange), planar ∥ (red), and planar ⊥ (green).

5- The statement (see page 13, line 247)"The loss factor, tan δ, shows a single peak that is associated with the glass transition at ~0 °C in both materials" is not consistent with the data. From the plotted curves, the tan δ peak appears substantially above 0 °C (≈ 10–15 °C). Please revise this statement to reflect the actual peak position. To avoid ambiguity, I recommend the authors to increases sweep range: the temperature sweep range appears too narrow to locate the loss peak unambiguously. Starting the DMA sweep at ~–50 °C and extending to at least +100 °C would better capture the glassy regime, and the rubbery plateau, thereby allowing a clear and reproducible identification of the peak position.

Again, we thank reviewer one for the useful feedback regarding the DMA experiments, and have extended the temperature range of the experiments as detailed above. The excerpt referred to by reviewer one now appears at line 332 (page 17) in the revised manuscript, has been updated to list the peak in the tanδ for LCE1 and LCE3, and is copied below for the reviewer's convenience:

'There is a single peak in tanδ which occurs at 12 °C in LCE1 and 15 °C in LCE3 that is associated with the glass transition.'

6- Using Equation 7 to calculate the adhesion factor by inserting the measured values of tan δ and G" is useful; however, it may not necessarily correlate with actual adhesion performance. I believe this paper would be significantly improved if the authors conducted a simple adhesion test to directly determine adhesion as a function of temperature. Such an adhesion test could include a tack-force measurement or a peel-force test.

We thank reviewer one for these suggestions, and agree that undertaking direct adhesion tests would indeed strengthen the assessment of the adhesive properties of the LCEs in this manuscript. To that end, we have undertaken probe tack and peel strength measurements on samples of LCE1 and LCE3. The results of these measurements have been added to the manuscript and are copied below for the reviewer's convenience:

'In addition to calculating a temperature-dependent adhesion factor, the experimentally-determined adhesion properties of LCE1 and LCE3 were compared at room temperature with those of the nematic LCE AN06 reported by Street *et al.*[28] First, peel tests, where a strip of LCE is peeled away from a glass surface at 90 °, were performed on homeotropic and planar samples of LCE3 with the planar director perpendicular to the peeling direction. The force per unit width required to debond LCE3 from the substrate is 0.0091 ± 0.004 Nmm^{-1} and 0.0140 ± 0.004 Nmm^{-1} in the planar and homeotropic cases respectively. The factor of 1.5 found between the homeotropic and planar cases compares well with the 2.0 times difference suggested by the adhesion factor at room temperature. This can be compared to literature results for the nematic AN06 where the planar orientation adheres more strongly than the homeotropic (0.043 Nmm^{-1} and 0.015 Nmm^{-1} respectively), values which are again strongly correlated with the adhesion factor.[28]

The adhesive properties of LCE1 and LCE3 were also probed through tack tests. Due to the geometry of the probe-tack test, removing the probe from a homeotropic LCE will incur the maximum penalty caused by the layer compression modulus (Figure 6a), and the planar LCE will incur a minor penalty. Figure 6b shows a typical tack result where the spherical probe is removed from the sample at a constant speed and the force measured. Figure 6c shows the average tack force of adhesion for LCE1, LCE3, and AN06 reported by Street *et al.*[28] In the planar alignment the tack strength is effectively the same for LCE1, LCE3, and AN06 (1.7 ± 0.1 N, 1.67 ± 0.09 N, and 1.72 ± 0.06 N) respectively. In the homeotropic geometry the tack strength in AN06 (1.54 ± 0.09 N) is similar to the tack strength in LCE3 (1.4 ± 0.1 N) and is significantly larger than in LCE1 (1.25 ± 0.09 N). This is attributed to the layer spacing modulus, b, which is much lower in LCE3 (b = 3.4 ± 0.1) than in LCE1 (b = 7.5 ± 0.1), influencing the anisotropic adhesion less. It is interesting to explore this finding in the context of the shear moduli; the peak magnitude of $tan\delta$ is comparable for all three of the materials being considered at around 0.6-0.8, though the average value at room temperature (where the peel tests were performed) is 0.50, 0.64, and 0.72 for LCE1, LCE3, and AN06 (Street *et al.*). It is noted that in the planar configuration all LCEs are similar in tack despite the differences in $tan\delta$ (0.55, 0.70, and 0.72 in LCE1, LCE3, and AN06) respectively). However in the homeotropic geometry, where the room temperature values of $tan\delta$ are 0.45, 0.57, and 0.72, there is a positive correlation between the measured tack and $tan\delta$. The ratio of the tack strength for the homeotropic and planar geometries is 1.4 and 1.2 for LCE1 and LCE3 respectively. This compares well with the 2.0 factor predicted at room temperature by the adhesion factor as determined *via* the shear moduli.

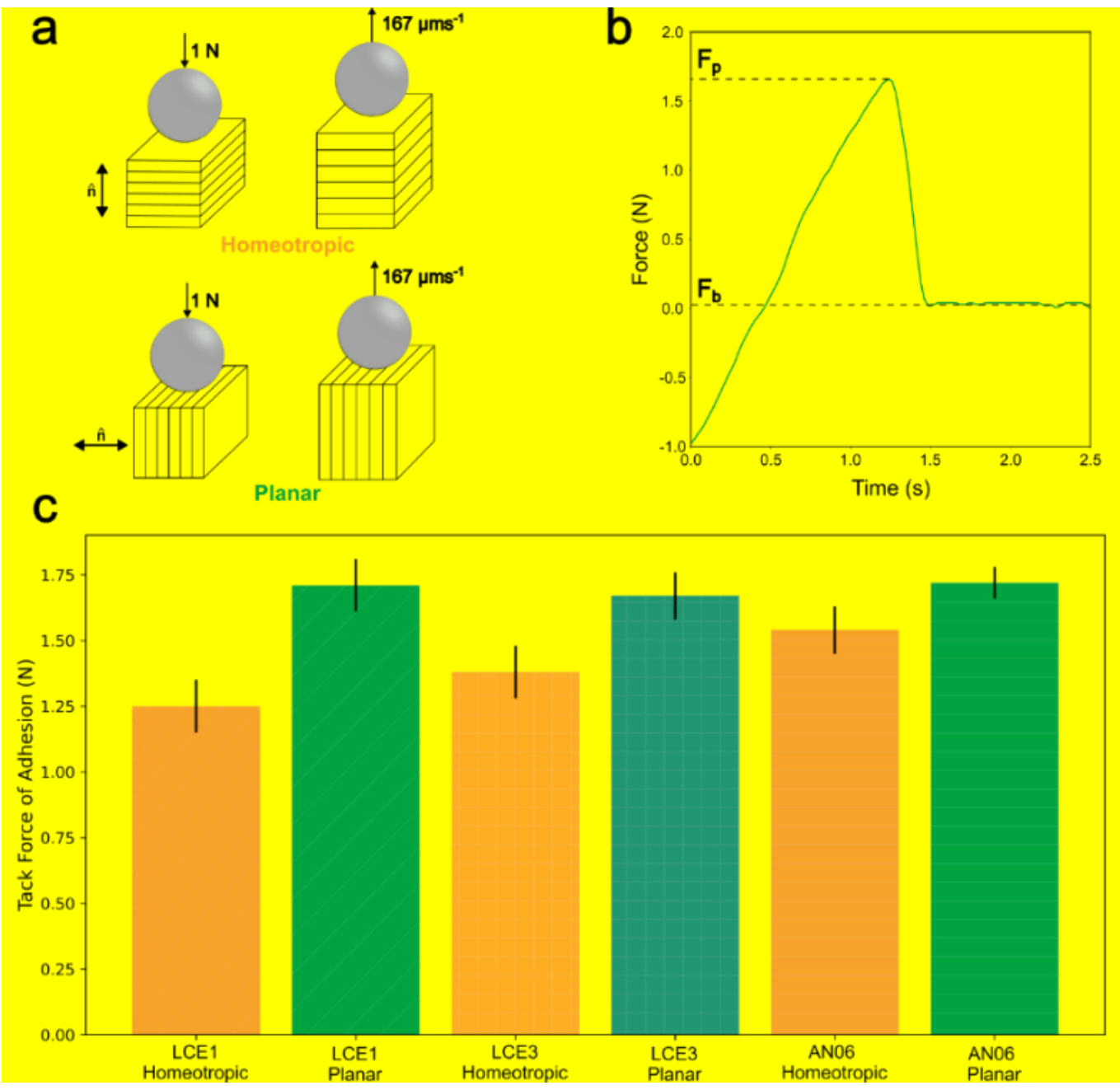

Figure 6. a) Schematic of the spherical probe being applied and removed from LCE samples aligned in homeotropic (orange) and planar (green) geometries. In the homeotropic case the removal force is parallel to the director and layer normal compared to the planar case where the removal force is perpendicular to the director. b) A typical tack-test result, after a dwell-time 30 s at a force of -1 N, the probe is removed and the force measured. The tack force of adhesion is the difference between the peak force (F_p) and the baseline force (F_b). c) Tack force of adhesion results for LCE1 (diagonal hatches), LCE3 (square hatches), and NLCE (horizontal hatches), for homeotropic (orange) and planar (green) alignments.'

Reviewer Two

This study reports for the first time the auxetic behavior of frustrated smectic-A liquid crystalline elastomers (SmA LCEs), expanding the family of molecular auxetic materials while uncovering their anisotropic shear modulus and adhesive properties. The research holds significant scientific value and application potential, and the overall work is well-founded for submission to Nature Communications. However, there remain aspects requiring further refinement and supplementation in terms of innovation, mechanistic elucidation, data completeness and research systematicity. The specific review comments are as follows:

We thank reviewer two for the constructive and insightful appraisal of our manuscript. The suggestions made by the reviewers have been considered, and below we provide a point-by-point breakdown as to how they have been addressed in the revised manuscript.

1. This paper reports a novel auxetic liquid crystalline elastomer (LCE) based on the frustrated smectic-A (SmA) phase, which exhibits notable innovation in the field of auxetic materials. However, the discussion section lacks a sufficient comparison of its core advantages over conventional nematic auxetic elastomers. It is recommended to systematically elucidate the specific advantages and potential application scenarios of frustrated SmA-phase LCEs relative to nematic LCEs, so as to highlight the scientific value and application potential of this work.

We thank reviewer two for this comment, and we agree that the discussion surrounding the significance of these LCEs exhibiting a frustrated smectic phase rather than a nematic phase could have been stronger in the original manuscript.

We feel that the main advantage to the frustrated smectic LCEs discussed in this work comes from the point of view of broadening the scope for controlling the auxetic response via chemical modification. Previous studies have shown that the auxetic response in nematic LCE can be modified by alterations to the chemical structure of the LCEs (DOI: 10.3390/polym16141957). However, it has also been shown that many of the chemical modifications attempted can lead to smectic phases as opposed to nematic phases (DOI: 10.3390/polym16141957, 10.1039/D5TC01484K).

Before discovering these first examples of frustrated smectic LCEs that could be strained sufficiently to show an auxetic response, we had expected that the chemical modification of the auxetic response was limited to the small pool of monomers that yielded nematic LCEs. The discovery of auxeticity in frustrated smectic LCEs, allows investigations into structure property relationships to be broadened and new LCEs to be designed. It is also possible (but yet to be explored) that smectic auxetic LCEs may have better properties than nematics. This potential for modification has been reflected in our revised manuscript, copied below for the reviewer's convenience:

'The importance of the discovery of a new auxetic system is rooted in the increased scope for molecular design that these findings imply. It had previously been expected that only nematic LCEs could exhibit auxetic behavior as the auxetic threshold strains were higher than the typical strain-failure observed in smectic LCEs (~30%).[8] However, the requirement of a nematic phase is a serious constraint; side-chain liquid crystal polymer systems such as the LCEs reported here are often dominated by smectic character. The observation of auxetic behavior in smectic LCEs provides impetus to reconsider side groups that would previously have been considered as too smectogenic, opening the door for new structural motifs to be incorporated into molecular auxetics. This broader palette of monomers provides a wider basis with which to optimize material properties for specific applications. Whilst such studies are the focus of future work, improved material properties are already be being seen in the frustrated smectic LCEs reported herein; for example, the auxetic threshold observed for LCE3 is the lowest reported for any auxetic LCE at a comparable strain rate. Auxetic smectic LCEs also offer moduli that are different both in magnitude and anisotropy from nematic systems, providing another dimension of modification for potential applications.'

As noted, in addition to the advantages in terms of possible chemical modification, we would also be interested to examine the potential advantages in terms of material properties that frustrated smectic LCEs could possess. Whilst this work has begun, detailed investigations are beyond the scope of this initial manuscript, as the factors that could influence these LCEs are wide-ranging, and it has taken a large body of work spanning 8 years, 14 journal articles and 4 patents to elucidate similar information in nematic LCEs.

2. The paper states that the threshold strains for the Poisson's ratio of LCE2, LCE3 and LCE4 are 0.41±0.05, 0.50±0.05 and 1.05±0.05, respectively. However, it fails to identify the core factors responsible for the differences in threshold strain among the various samples, merely mentioning that there is no obvious correlation with the glass transition temperature. It is recommended to conduct an in-depth analysis of the causes for such differences in threshold strain by combining relevant structural parameters of the samples, such as crosslink density and smectic correlation

length.

We agree that in depth studies of the factors influencing the auxetic response in LCEs would be of significant interest for both the LCE community, and the scientific community in general. During the revision process, we have undertaken some initial experiments towards the goal of better understanding these factors, by assessing the effect of strain rate on the auxetic response. These new findings and a brief discussion of them have been added to the manuscript, and are copied below for the reviewer's convenience:

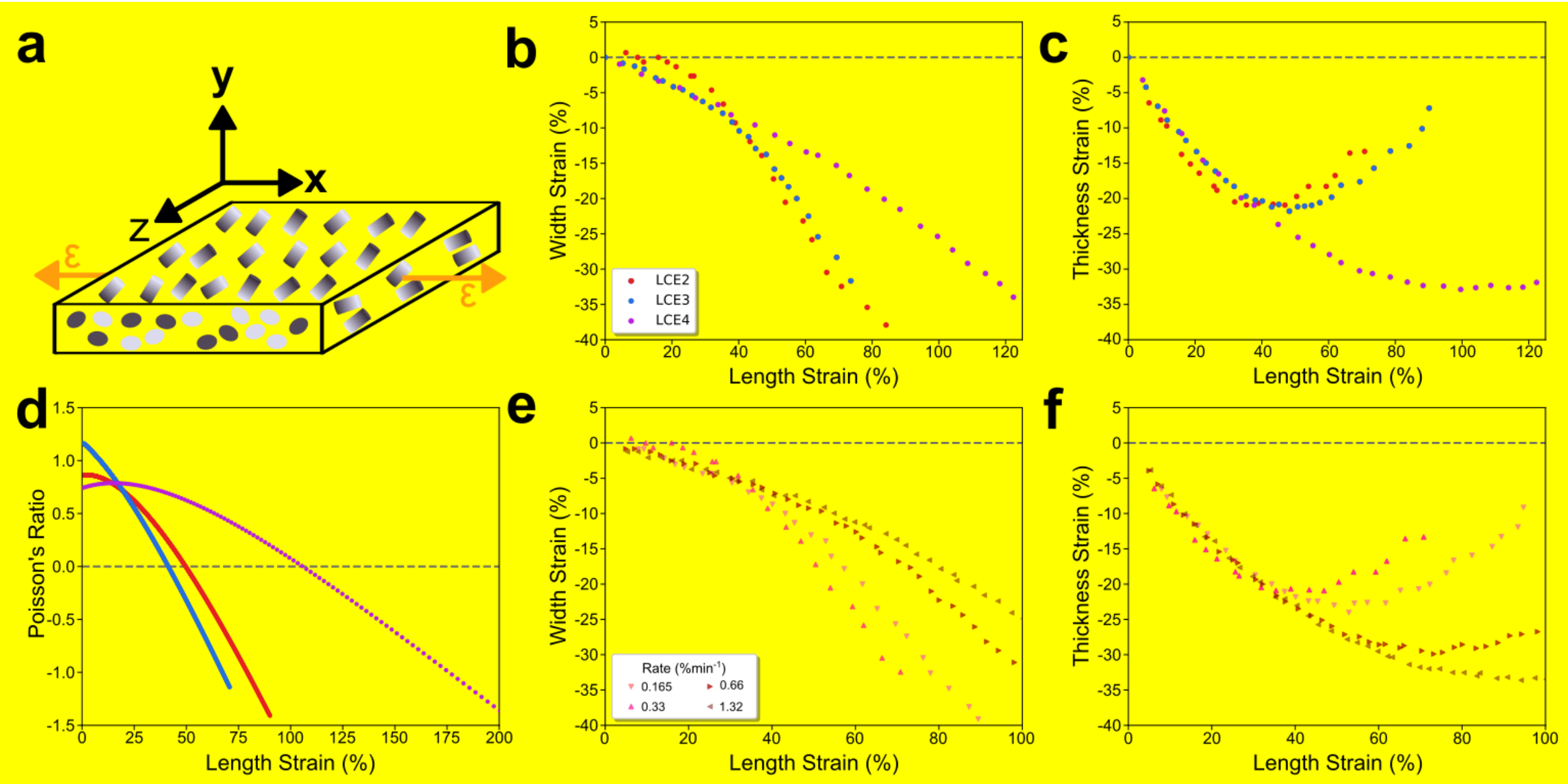

Figure 3. (a) A schematic representation of the experimental conditions employed in the analysis of auxeticity in the LCE samples, defining the naming convention of the x (length), y (thickness) and z (width) axes, where ε represents the applied strain and the rods depict the alignment of the mesogens in the LCE. Panels (b) and (c) show the effect of an applied length (x) strain on the sample dimensions in the width (z) and thickness (y) dimensions respectively for each sample when strained at a rate of 0.33%/min at room temperature. An increase in sample thickness occurs above threshold length strains of 41±5%, 50±5% and 105±5% for LCE2, LCE3 and LCE4 respectively. LCE1 consistently failed at strains of ~30%. d) Shows the instantaneous Poisson's ratio in the y-axis (ν) deduced for each sample as a function of the applied x-strain. e) and f) Show the measured strain (in width and length respectively) for LCE2 strained at different rates: 0.165%/min (down triangles), 0.33%/min (upright triangles), 0.66%/min (right-facing triangles), and 1.32%/min (left-facing triangles). The auxetic thresholds become 53±5%, 47±5%, and 73±5% for 0.5x, 1x, and 2x respectively, an auxetic threshold is not observed at a rate of 4x up to a strain of 100% although the initial strain behavior is similar.

'For auxetic nematic LCEs, it has previously been observed that the auxetic threshold strain is dependent on several factors, most notably dynamics, for example the temperature or the rate at which strain is applied to the sample.[8,33] Given that the layered structure present in smectic LCEs adds more geometric considerations than in nematic LCEs, we were interested to undertake some initial studies to explore whether the strain rate similarly impacts the auxetic response in these smectic LCE systems. In these experiments, samples of LCE2 were examined at different strain rates, namely 0.165%/min, 0.33%/min, 0.66%/min and 1.32%/min, and the data are shown in Figure 3e and f.

In nematic auxetic LCEs, it has been reported that as the strain rate is increased, the auxetic threshold of the nematic LCE increases, as faster strain rates can be thermorheologically related to lower temperatures.[33] The data reported in Figure 3e and f suggest that in the frustrated smectic LCEs there is a similar strain-rate dependence. Taking the rate of 0.33%/min as the base strain rate as

this is the rate used to compare samples of different composition (Figure 3b, c and d), it is observed that as the strain rate is increased (doubled and quadrupled to 0.66%/min and 1.32%/min respectively), the auxetic threshold also increases. This increase is such that for the sample strained at 1.32%/min, no auxetic response is observed prior to sample failure. Interestingly, we find that if the strain rate is halved from the original 0.33%/min to 0.165%/min, the auxetic threshold remains comparable. One may expect a decrease in the auxetic threshold based on this change in strain rate, so this result is somewhat surprising. This finding may result from a constraint imposed by the layered structure present in the frustrated smectic sample, or it may simply be that the change in strain rate imposed was insufficient to substantially change the dynamics of the system. These initial findings suggest that a more complex relationship potentially exists between strain rate and auxetic threshold in these frustrated smectic systems than in the nematic case. A future, more detailed study considering factors such as strain rate and temperature will be conducted to deduce this relationship.

Furthermore, it has been previously reported that the auxetic response in nematic LCEs appears to be governed by proximity to the glass transition temperature (Tg) of the sample, with a higher Tg leading to higher auxetic thresholds.[8,33] For the smectic auxetic LCEs reported here, there is no facile relationship between Tg and the auxetic threshold; LCE2, LCE3 and LCE4 have a Tg of 7 °C, 0 °C and 1 °C by differential scanning calorimetry respectively (Table S1), yet the auxetic thresholds follow the order LCE3 < LCE2 < LCE4, indicating a non-monotonic relationship. These results therefore suggest that the auxetic response in smectic LCEs may be influenced by more characteristics than those known to influence the auxetic response in nematic LCEs. Attempts to elucidate these characteristics will be the subject of future studies.'

As discussed in response to reviewer one, we feel that given the body of literature it has taken for the auxetic response to be understood for the nematic LCEs (14 journal articles, 4 patents), a detailed assessment of several factors, as reviewer two is suggesting we include, would be beyond the scope of this manuscript. For example, reviewer two specifies the effect of cross-link density would be of interest, which we agree with. However, such an investigation in the auxetic nematic LCEs itself forms a singular journal article (DOI: 10.1039/D5SM00677E), thus we feel to include such a study as part of a larger body of work such as this one, would not be doing justice to either study.

3. Although Figure 3 shows the effect of strain on the thickness and width of the samples, it lacks characterization results of the micromorphology before and after the strain exceeds the threshold, making it difficult to directly reflect the structural evolution process. Supplementary characterization of the actual state of the samples at this stage is suggested, including molecular orientation and smectic layer structural changes at the microscale, as well as sample morphology and transparency at the macroscale, to provide direct experimental evidence for the intrinsic mechanism of thickness variation.

We thank reviewer two for this suggestion. Further characterisation of the LCEs under strain has been conducted, which has allowed a more detailed discussion of the mechanism behind the auxetic response in these samples to be added to the manuscript. These experiments take into account the molecular reorientation and observations regarding smectic layer spacing changes during strain. The updated discussion and data is copied below for the reviewer's convenience:

'The auxetic response in nematic LCEs is driven by a biaxial deformation, in which an increasing proportion of mesogens align in the transverse axis when strain is applied perpendicular to the initial

director.[10,11] As frustrated smectic LCEs exhibit a layered structure on a molecular level, we were interested to examine if this same biaxial deformation existed in these materials, and hence was the cause of the auxetic response. A combination of polarized light optical microscopy, conoscopy, and polarised Raman spectroscopy was used to examine the emergence of biaxiality in these samples (Figure 4).'

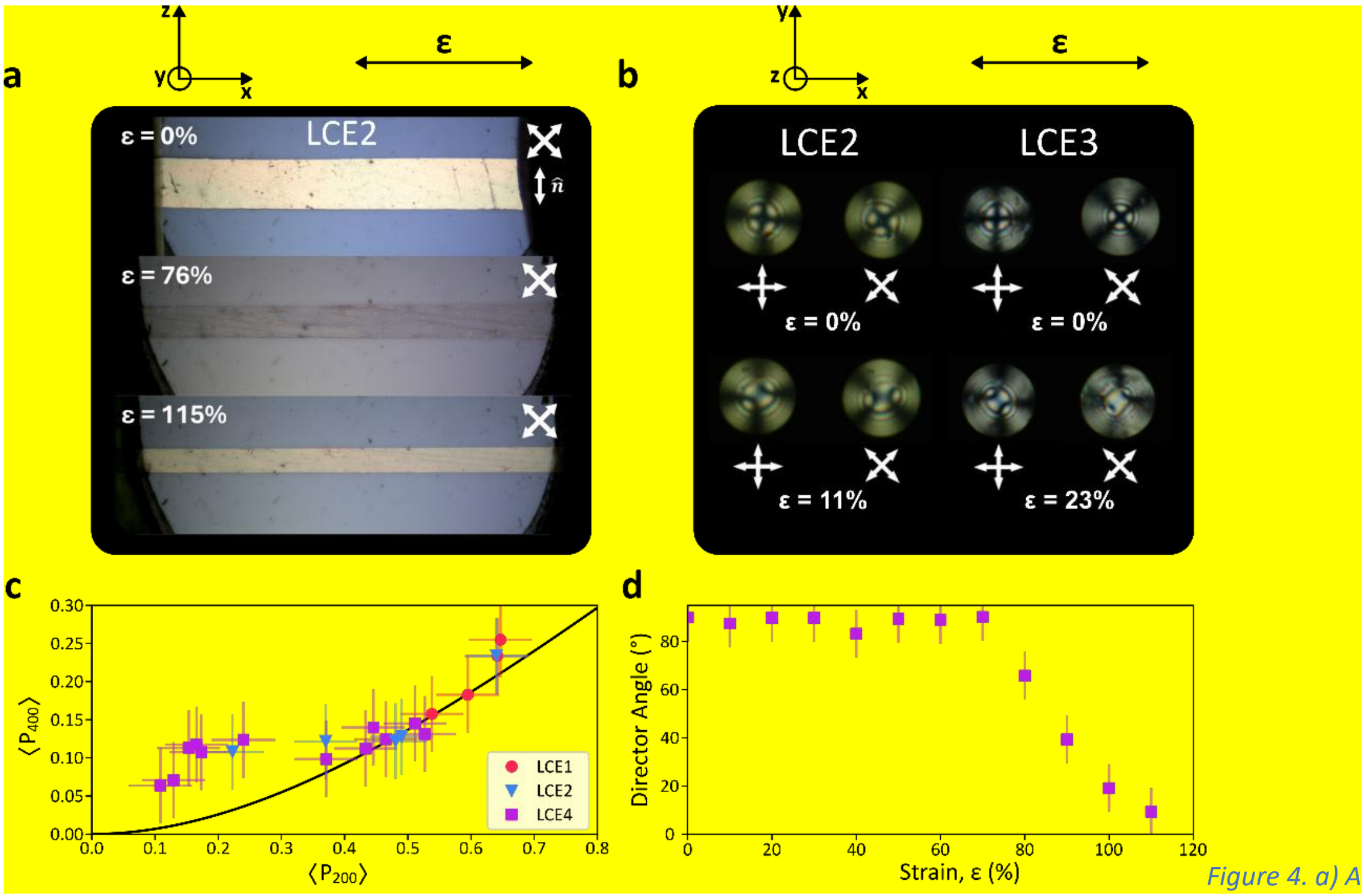

Figure 4. a) A sample of planar LCE2 examined as a function of strain (ε) under crossed-polarisers, with the initial director (n) indicated by the arrow on the topmost image. The transition from a bright state to a dark state, and then back to the bright state is clearly observed, consistent with behaviour observed for nematic auxetic LCE. b) Conoscopic figures for homeotropic LCE2 and LCE3, showing a Maltese cross indicative of uniaxial order when unstrained and two melatopes when strained (ε ≠ 0%), indicative of the emergence of biaxial order.

Figure 4. a) A sample of planar LCE2 examined as a function of strain (ε) under crossed-polarisers, with the initial director (n) indicated by the arrow on the topmost image. The transition from a bright state to a dark state, and then back to the bright state is clearly observed, consistent with behaviour observed for nematic auxetic LCE. b) Conoscopic figures for homeotropic LCE2 and LCE3, showing a Maltese cross indicative of uniaxial order when unstrained and two melatopes when strained (ε ≠ 0%), indicative of the emergence of biaxial order. c) The fourth-order uniaxial order parameter, $<P_{400}>$, as a function of the second-order uniaxial order parameter, $<P_{200}>$, for LCE1 (red circles), LCE2 (blue inverted triangles), and LCE4 (purple squares) determined by polarised Raman spectroscopy. Both order parameters reduce in magnitude with increasing strain. A clear deviation from the Maier-Saupe theoretical prediction (black line) is seen as the LCE is strained, this is indicative of the emergence of biaxial order. d) The apparent director angle as a function of strain in LCE4, an apparent rapid reorientation begins at 70% strain.

When samples are visualised under crossed polarisers (Figure 4a for LCE2), the samples initially show a uniform bright state. Upon the application of strain, this bright state persists until a critical strain (76%) at which point, the birefringence of the sample becomes zero and a uniform dark state is observed. Upon further strain, the sample begins to show birefringence colour again. These observations suggest a director reorientation process is occurring upon strain, and are entirely analogous to observations made for nematic auxetic LCEs.[5] The emergence of biaxiality in the samples is directly observed via conoscopic studies of homeotropically aligned samples. Prior to the application of strain, the samples show a Maltese cross in the conoscopic figure (Figure 4b), indicative of uniaxial order. Upon application of strain, the splitting of the Maltese cross into two melatopes indicates biaxial order, consistent with observations for nematic auxetic LCEs[10].

As conoscopy can only visualise biaxiality up to relatively low strains (after which the melatopes leave the field of view), the order parameters were measured for the LCEs at strains up to 120% using polarized Raman Spectroscopy. Figure 4c) shows the $\langle P_{200} \rangle$ and $\langle P_{400} \rangle$ order parameter(s) for the LCE samples as a function of strain. Plotting $\langle P_{200} \rangle$ versus $\langle P_{400} \rangle$ shows that as strain is increased, the order parameters reduce in magnitude and deviate from Maier-Saupe theory, a behaviour that can also be attributed to the emergence of biaxial order during strain, and again consistent with the observations made for nematic auxetic LCEs.[11] The in-plane director angle can also be monitored from the Raman scattering data, Figure 4d. The behaviour observed is typical of that previously reported in LCEs as a mechanical Fréedericksz transition, now known to be associated with a biaxial deformation.[10,11]

These combined studies provide conclusive evidence that a biaxial deformation is driving the auxetic response in these frustrated smectic LCEs. We attribute the layer frustration, and the resulting softening of layer compression moduli, as being a driving factor that allows the biaxial response to continue to high strains, and hence for the auxetic response to be observed. It is also of note that some of the frustrated smectic LCEs have previously been examined under strain via X-ray scattering,[12] and show minimal evidence of buckling instabilities, or changes in layer spacing (with layer spacing varying by at most 0.5% during the experiment). However, these experiments are only able to elucidate information at small strains due to experimental difficulties, with more intensive studies set to be the focus of future work.'

With regards to macroscale parameters as suggested by the reviewer, we have included example images taken throughout the strain experiment (Figure 1f and Figure 4a), showing that the LCEs remain transparent throughout the straining process, strongly indicative of no change or evolution of pores on micrometre length scales, during the straining process. All of the above findings are consistent with the observations made for nematic auxetic LCEs, allowing us to conclude that the mechanism of the auxetic response is the same as that in the nematic LCEs. We cannot think of any further micromorphological studies that would shed more light on the mechanism. We have previously used atomic force microscopy and scanning electron microscopy on nematic samples (DOI: 10.1038/s41467-018-07587-y) which confirmed that no pores evolved at nm length scales and expect no new information if such (highly technically challenging) measurements were attempted on these samples.

4. Using this method, how large of the film can be prepared?

Whilst this work concerns samples that are made to the dimensions of a microscope slide (7.5 cm x 2.5 cm), it has been demonstrated that the fabrication process is scalable; our spin-out company, Auxetec Ltd., regularly fabricates auxetic LCE sheets up to A4 size using an equivalent process to the lab-based one reported in this work. They have also recently reported successful trials of roll-to-roll production of similar LCEs, using a process adapted for larger scale manufacturing. An example of larger scale samples can be seen at the following link: https://www.youtube.com/watch?v=luMq9XA0hKI. As that work is commercial rather than academic, we chose not to include it in this article, but could make reference the video, if the editors felt it appropriate.

5. Errors exist in the ordinate labels of Figure 4d and Figure 4h. The authors are suggested to carefully check the axis labels and physical units of all figures and tables throughout the manuscript

to avoid similar labeling errors and ensure the accuracy and readability of data presentation.

We apologise for the oversight in our original manuscript, and thank reviewer two for their thorough assessment of the manuscript which enabled them to discover these errors. We have corrected the highlighted labels, which now appear in Figure 5d and 5h, and have ensured that all figures in the revised manuscript are free from any such errors.

Reviewer Three

The manuscript reports the synthesis, characterization and analysis of frustrated smectic liquid crystal elastomers which show negative Poisson's ratio beyond a threshold strain and anisotropic adhesion. The study reports a new class of liquid crystal elastomers with auxetic behaviors (negative Poisson's ratio) beyond previously reported nematic liquid crystal elastomers (LCEs). While there have been many studies reporting auxetic behaviors at structure level by designing "metamaterials", materials with auxetic behaviors at atomic or molecular level are rare. Thus, there could be potential new opportunities from the findings which can be of interest to the readership of Nature Communications. The manuscript would be suitable for publication after addressing the following questions, comments, and suggestions.

We would like to thank reviewer three for their positive and constructive assessment of our manuscript. The suggestions made have been considered and incorporated into the manuscript where appropriate, and below we provide a point-by-point discussion as to how each suggestion has been addressed.

1. The title of the manuscript is "Frustrated Smectic Liquid Crystal Elastomers as Multifunctional Mechanical Metamaterials". However, there seem to be no supporting data regarding multifunctional aspects of the materials. It will be helpful to add relevant information.

We chose this title as the work details two functionalities of frustrated smectic LCEs; auxeticity and adhesion. Upon reflection, we understand reviewer three's comment, as we included relatively little information on adhesion in the original manuscript. We have now enhanced the discussion surrounding the adhesive properties of these materials, copied below for the reviewer's convenience, and hence have strengthened the multifunctional aspect of the manuscript.

'In addition to calculating a temperature-dependent adhesion factor, the experimentally-determined adhesion properties of LCE1 and LCE3 were compared at room temperature with those of the nematic LCE AN06 reported by Street *et al.*[28] First, peel tests, where a strip of LCE is peeled away from a glass surface at 90 °, were performed on homeotropic and planar samples of LCE3 with the planar director perpendicular to the peeling direction. The force per unit width required to debond LCE3 from the substrate is 0.0091 ± 0.004 Nmm^{-1} and 0.0140 ± 0.004 Nmm^{-1} in the planar and homeotropic cases respectively. The factor of 1.5 found between the homeotropic and planar cases compares well with the 2.0 times difference suggested by the adhesion factor at room temperature. This can be compared to literature results for the nematic AN06 where the planar orientation adheres more strongly than the homeotropic (0.043 Nmm^{-1} and 0.015 Nmm^{-1} respectively), values which are again strongly correlated with the adhesion factor.[28]

The adhesive properties of LCE1 and LCE3 were also probed through tack tests. Due to the geometry of the probe-tack test, removing the probe from a homeotropic LCE will incur the maximum penalty caused by the layer compression modulus (Figure 6a), and the planar LCE will incur a minor penalty. Figure 6b shows a typical tack result where the spherical probe is removed from the sample at a constant speed and the force measured. Figure 6c shows the average tack force of adhesion for LCE1, LCE3, and AN06 reported by Street *et al.*[28] In the planar alignment the tack strength is effectively the same for LCE1, LCE3, and AN06 (1.7 ± 0.1 N, 1.67 ± 0.09 N, and 1.72 ± 0.06 N) respectively. In the homeotropic geometry the tack strength in AN06 (1.54 ± 0.09 N) is similar to the tack strength in LCE3 (1.4 ± 0.1 N) and is significantly larger than in LCE1 (1.25 ± 0.09 N). This is attributed to the layer spacing modulus, b, which is much lower in LCE3 (b = 3.4 ± 0.1) than in LCE1 (b = 7.5 ± 0.1), influencing the anisotropic adhesion less. It is interesting to explore this finding in the context of the shear moduli; the peak magnitude of $tan\delta$ is comparable for all three of the materials being considered at around 0.6-0.8, though the average value at room temperature (where the peel tests were performed) is 0.50, 0.64, and 0.72 for LCE1, LCE3, and AN06 (Street *et al.*). It is noted that in the planar configuration all LCEs are similar in tack despite the differences in $tan\delta$ (0.55, 0.70, and 0.72 in LCE1, LCE3, and AN06) respectively). However in the homeotropic geometry, where the room temperature values of $tan\delta$ are 0.45, 0.57, and 0.72, there is a positive correlation between the measured tack and $tan\delta$. The ratio of the tack strength for the homeotropic and planar geometries is 1.4 and 1.2 for LCE1 and LCE3 respectively. This compares well with the 2.0 factor predicted at room temperature by the adhesion factor as determined *via* the shear moduli.

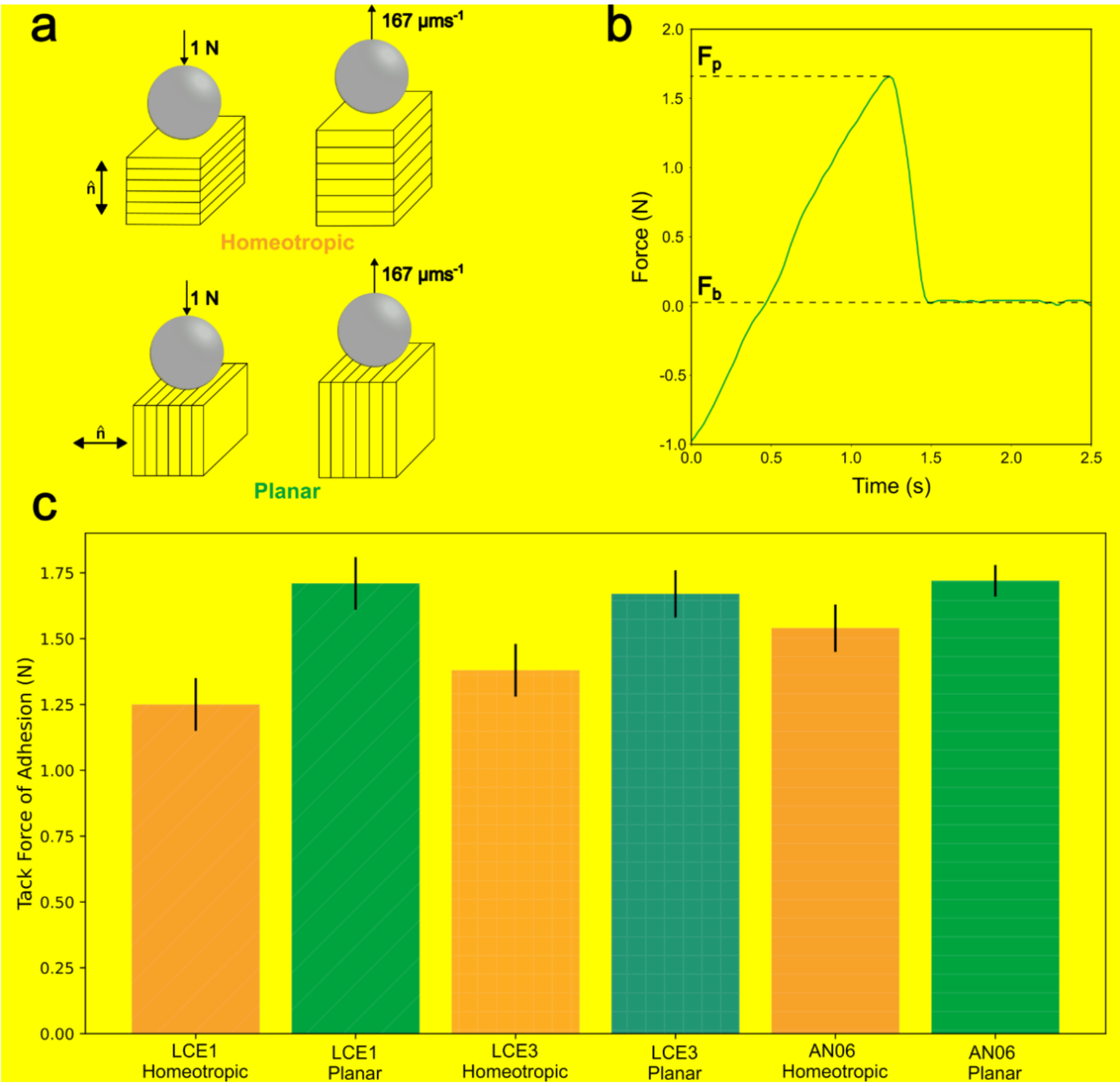

Figure 6. a) Schematic of the spherical probe being applied and removed from LCE samples aligned in homeotropic (orange) and planar (green) geometries. In the homeotropic case the removal force is parallel to the director and layer normal compared to the planar case where the removal force is perpendicular to the director. b) A typical tack-test result, after a dwell-time 30 s at a force of -1 N,the probe is removed and the force measured. The tack force of adhesion is the difference between the peak force (F_p) and the baseline force (F_b). c) Tack force of adhesion results for LCE1 (diagonal hatches), LCE3 (square hatches), and NLCE (horizontal hatches), for homeotropic (orange) and planar (green) alignments.'

2. It will be helpful to strengthen the introduction and the discussion part regarding the advantages of the frustrated smectic liquid crystal elastomers compared with nematic liquid crystal elastomers so that readers can appreciate the potential importance of the findings.

We thank reviewer three for this comment, and, especially given a similar comment was made by reviewer two, we agree that the discussion surrounding the significance of these LCEs exhibiting a frustrated smectic phase rather than a nematic phase could have been stronger in the original manuscript. We previously expected that chemical modifications to auxetic LCEs were limited to those that facilitated the retention of nematic phase, especially as smectic LCEs were expected to fail at strains lower than typical auxetic thresholds (~0.3). The significance of the results in this manuscript is that we have now shown that smectic LCEs are capable of exhibiting auxetic behaviour, allowing a much larger variety of monomers and chemical moieties, and hence more possibilities in

the design of new auxetic LCEs. We have strengthened our discussion of this to address the reviewer's comment, copied below for the reviewer's convenience:

'The importance of the discovery of a new auxetic system is rooted in the increased scope for molecular design that these findings imply. It had previously been expected that only nematic LCEs could exhibit auxetic behavior as the auxetic threshold strains were higher than the typical strain-failure observed in smectic LCEs (~30%).[8] However, the requirement of a nematic phase is a serious constraint; side-chain liquid crystal polymer systems such as the LCEs reported here are often dominated by smectic character. The observation of auxetic behavior in smectic LCEs provides impetus to reconsider side groups that would previously have been considered as too smectogenic, opening the door for new structural motifs to be incorporated into molecular auxetics. This broader palette of monomers provides a wider basis with which to optimize material properties for specific applications. Whilst such studies are the focus of future work, improved material properties are already be being seen in the frustrated smectic LCEs reported herein; for example, the auxetic threshold observed for LCE3 is the lowest reported for any auxetic LCE at a comparable strain rate. Auxetic smectic LCEs also offer moduli that are different both in magnitude and anisotropy from nematic systems, providing another dimension of modification for potential applications.'

3. In the introduction, it was mentioned that molecular auxetic materials could open new opportunities, but there was no data or demonstration in this paper that show clear evidence. It will be helpful and critical to add data that show unique potential of molecular auxetic materials that no other "micro/macro-structural" auxetic materials can have.

We thank reviewer three for this comment. In the original manuscript, the relevant section of the introduction originally read:

'The discovery of a family of side chain nematic liquid crystal elastomers (Figure 1a and b[5]) as the first synthetic molecular auxetics was a breakthrough that transformed the range of possible applications for auxetic materials. In addition to the known benefits of impact and delamination resistance seen by all auxetics, LCEs are the only known transparent[6] auxetics, are scalable from the nanometer regime upwards[7], are highly tunable[8], and offer the potential of facile large-scale manufacture[9].'

Whilst we felt that the references highlighted within the relevant excerpt of text did signpost the advantages of these molecular auxetics, we understand that reviewer three is suggesting that some slight elaboration on these advantages could be useful for readers. We have therefore strengthened the discussion of these advantages, with the addition of the following discussion:

'These additional features make auxetic LCEs exciting options for applications where porous auxetics may be unsuitable. For example, the transparency of the auxetic LCEs (94% at 589 nm)[6] makes them exciting candidates in applications where optical clarity is imperative, something that traditional porous auxetics cannot achieve.'

4. Furthermore, it will be helpful to have examples where frustrated smectic liquid crystal elastomers show superior properties. In the introduction, it was discussed that auxetic materials can offer mechanical advantages such as resistance to impact, delamination and tear. However, there is no data comparing performance of the reported material with data from other materials including other liquid crystal elastomers. So, having such quantitative comparisons will provide useful guidelines to

potential users.

This report emphasises that we have discovered a new family of auxetic LCEs and that the potential to employ additional chemical design offers an opportunity to develop materials with different properties. Whether a particular property is superior or not depends on the application, and as with all new materials, applications follow discovery by some years. We have demonstrated in this report that, for example, the smectic LCEs have highly anisotropic shear moduli and that the magnitudes of the moduli span different scales, offering the potential to tune the mechanical response over a wider range than the nematic LCEs allow:

'The storage moduli take similar values for both materials plateauing above room temperature to values between around 0.1 – 1.0 MPa, but with a much higher anisotropy observed for LCE1 where the planar perpendicular case takes values around an order of magnitude higher than either of the other two geometries. The moduli are slightly higher than those reported for the nematic analogue, AN06.[28]'

We also noted very low values of Tg and auxetic threshold in these smectic LCEs. While the elucidating the structure-property relationships will be the subject of significant future work, even at this early stage, we have demonstrated that some properties of these smectic LCEs are superior to some of the auxetic nematic LCEs reported. For example, these smectic LCEs exhibit lower auxetic threshold values than analogous nematic systems examined at the same strain rate:

'The sample thickness initially decreases for all systems but above a threshold value (50 ± 5%, 41 ± 5%, and 105 ± 5% for LCE2, LCE3, and LCE4, respectively) the sample thickness begins to increase, i.e. the Poisson's ratios (Figure 3 (d)) become negative. These results therefore demonstrate for the first time that when sufficiently high strains can be achieved in some frustrated SmA side-chain LCEs, an auxetic response can be readily observed. It is noteworthy that the auxetic threshold values of LCE2 and LCE3 are lower than any previously reported for nematic LCEs (≥56%),8,10,32 showing an improved response of these systems.'

5. Here are some minor suggestions/questions/comments to improve the clarity for readers:

1) Lines 13, 31: be specific about the type of strain

The type of strain has been clarified as suggested, copied below for the reviewer's convenience:

'Auxetics are materials that show a negative Poisson's ratio, i.e. thickening when under tensile strain.'

'Auxetics are a class of mechanical metamaterials that exhibit negative rather than positive Poisson's ratios, meaning they expand under tensile strain rather than contracting, offering mechanical advantages that include resistance to impact, delamination and tear.[1,2]'

2) Line 46: discuss what are the factors that determine the auxetic strain-threshold

While elucidating the factors that influence the auxetic threshold in these systems is important, that is a topic of ongoing research and a detailed study goes beyond this report. Understanding the relevant factors in nematic LCEs represents a substantial body of work, covering 14 journal articles and 4 patents, owing to the array of factors that influence the auxetic response and we expect understanding smectic systems to offer a similarly complex challenge. Nonetheless, we have conducted some further experiments that demonstrate the impact of strain rate on the auxetic

response, as suggested by reviewer one. We believe this is an important first step in elucidating the factors that influence the auxetic response. The relevant discussion is copied below for the reviewer's convenience:

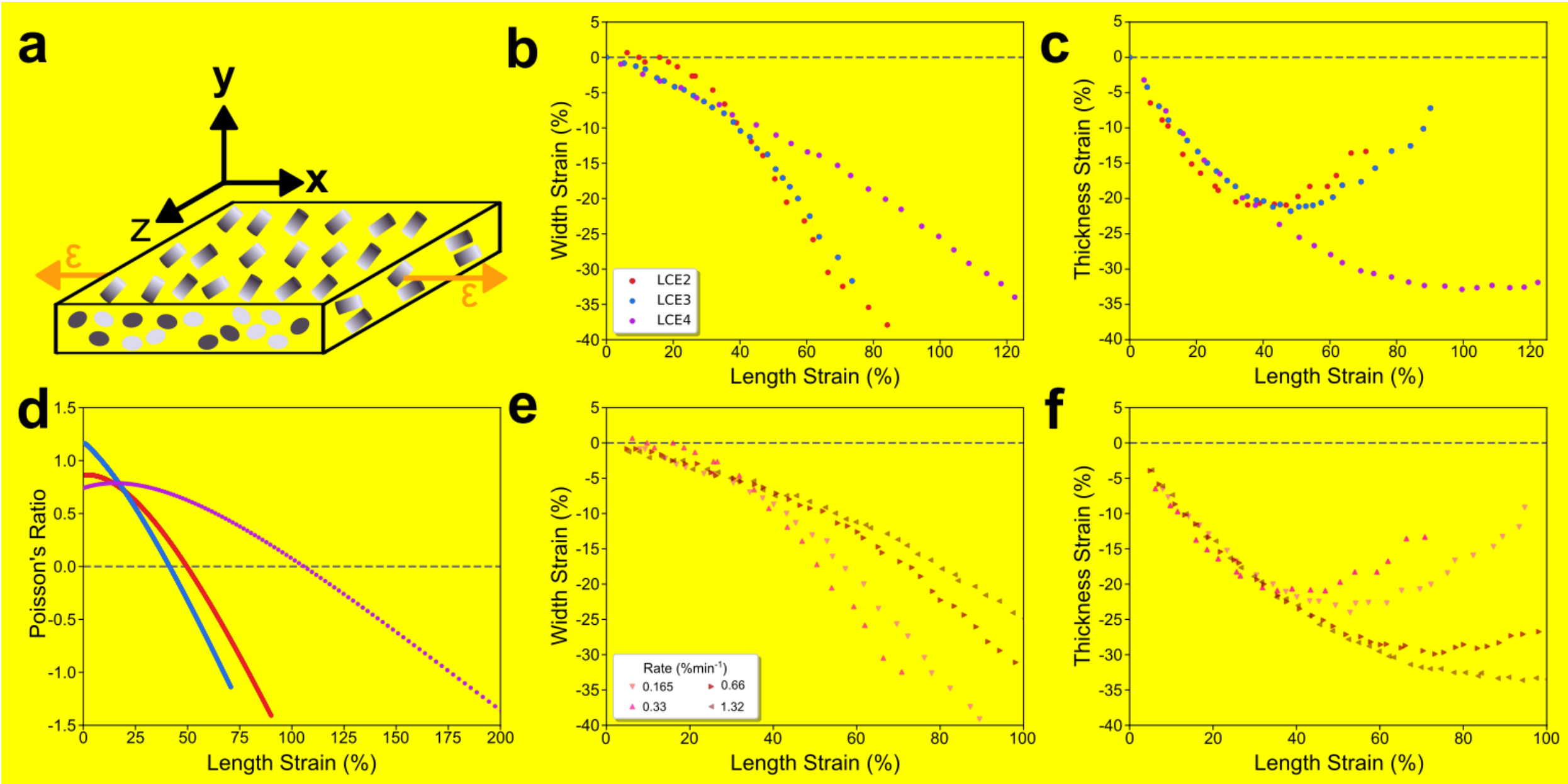

Figure 3. (a) A schematic representation of the experimental conditions employed in the analysis of auxeticity in the LCE samples, defining the naming convention of the x (length), y (thickness) and z (width) axes, where ε represents the applied strain and the rods depict the alignment of the mesogens in the LCE. Panels (b) and (c) show the effect of an applied length (x) strain on the sample dimensions in the width (z) and thickness (y) dimensions respectively for each sample when strained at a rate of 0.33%/min at room temperature. An increase in sample thickness occurs above threshold length strains of 41±5%, 50±5% and 105±5% for LCE2, LCE3 and LCE4 respectively. LCE1 consistently failed at strains of ~30%. d) Shows the instantaneous Poisson's ratio in the y-axis (v) deduced for each sample as a function of the applied x-strain. e) and f) Show the measured strain (in width and length respectively) for LCE2 strained at different rates: 0.165%/min (down triangles), 0.33%/min (upright triangles), 0.66%/min (right-facing triangles), and 1.32%/min (left-facing triangles). The auxetic thresholds become 53±5%, 47±5%, and 73±5% for 0.5x, 1x, and 2x respectively, an auxetic threshold is not observed at a rate of 4x up to a strain of 100% although the initial strain behavior is similar.

'For auxetic nematic LCEs, it has previously been observed that the auxetic threshold strain is dependent on several factors, most notably dynamics, for example the temperature or the rate at which strain is applied to the sample.[8,33] Given that the layered structure present in smectic LCEs adds more geometric considerations than in nematic LCEs, we were interested to undertake some initial studies to explore whether the strain rate similarly impacts the auxetic response in these smectic LCE systems. In these experiments, samples of LCE2 were examined at different strain rates, namely 0.165%/min, 0.33%/min, 0.66%/min and 1.32%/min, and the data are shown in Figure 3e and f.

In nematic auxetic LCEs, it has been reported that as the strain rate is increased, the auxetic threshold of the nematic LCE increases, as faster strain rates can be thermorheologically related to lower temperatures.[33] The data reported in Figure 3e and f suggest that in the frustrated smectic LCEs there is a similar strain-rate dependence. Taking the rate of 0.33%/min as the base strain rate as this is the rate used to compare samples of different composition (Figure 3b, c and d), it is observed that as the strain rate is increased (doubled and quadrupled to 0.66%/min and 1.32%/min respectively), the auxetic threshold also increases. This increase is such that for the sample strained at 1.32%/min, no auxetic response is observed prior to sample failure. Interestingly, we find that if the strain rate is halved from the original 0.33%/min to 0.165%/min, the auxetic threshold remains

comparable. One may expect a decrease in the auxetic threshold based on this change in strain rate, so this result is somewhat surprising. This finding may result from a constraint imposed by the layered structure present in the frustrated smectic sample, or it may simply be that the change in strain rate imposed was insufficient to substantially change the dynamics of the system. These initial findings suggest that a more complex relationship potentially exists between strain rate and auxetic threshold in these frustrated smectic systems than in the nematic case. A future, more detailed study considering factors such as strain rate and temperature will be conducted to deduce this relationship.

Furthermore, it has been previously reported that the auxetic response in nematic LCEs appears to be governed by proximity to the glass transition temperature (Tg) of the sample, with a higher Tg leading to higher auxetic thresholds.[8,33] For the smectic auxetic LCEs reported here, there is no facile relationship between Tg and the auxetic threshold; LCE2, LCE3 and LCE4 have a Tg of 7 °C, 0 °C and 1 °C by differential scanning calorimetry respectively (Table S1), yet the auxetic thresholds follow the order LCE3 < LCE2 < LCE4, indicating a non-monotonic relationship. These results therefore suggest that the auxetic response in smectic LCEs may be influenced by more characteristics than those known to influence the auxetic response in nematic LCEs. Attempts to elucidate these characteristics will be the subject of future studies.'

3) Line 63: check the typo

We thank reviewer three for highlighting this typographical error, which has now been corrected.

4) Line 64: check the font

We thank reviewer three for highlighting this formatting error, which has now been corrected.

5) Figure 1: it will be helpful to have a diagram visualizing geometric frustration of the smectic LCEs that the authors report so that readers can have a better understanding of the underlying mechanism and the molecular structure of the material.

Reviewer three makes an important point that visualising the frustration in these smectic LCE systems is difficult. However, for precisely that reason, we feel it is not possible to provide an accurate schematic representation of the order present in these LCEs. The frustration of the layered order in these smectic systems is evidenced by the relatively short-ranged correlations we observed based on well-established X-ray scattering measurements. However, exactly how this order diminishes across the bulk material, is not something we have been able to support with conclusive evidence. Gaining such evidence would require extremely technically challenging experiments, that currently are beyond the scope of this work. As such, we do not feel it appropriate to suggest a visual representation of the order in these systems without the conclusive evidence to support our choice of representation.

6) Line 168: check whether figures are labeled correctly.

We apologise for the oversight in our original manuscript, and thank reviewer three for their thorough assessment of the manuscript which enabled them to discover these errors. We have corrected the labels, which in the revised manuscript appear in Figure 5, and have ensured that all figures in the revised manuscript are free from any such errors.

7) Eq. 1: define mu, T, r, and b

The terms mu, r and b were defined in the original manuscript in the sentences immediately following equation 1, copied below for the reviewer's convenience:

'The first term is the same trace formula as in nematic LCEs where μ is the linear shear modulus, $\underline{\underline{l}}$ the step length tensor which is expressed in terms of the step length anisotropy, r, $\underline{\underline{l}}$ = Diag(1, 1, r), and $\underline{\underline{\lambda}}$ is the deformation tensor[13]. The second term relates to the smectic layer spacing, with layer spacing modulus $B = b/\mu$, and the initial director is defined along the z-axis, i.e. $n_0 = (0,0,1)^T$. The expression $1/\left|\underline{\underline{\lambda}}^{-T} \cdot n_0\right|$ represents the smectic layer spacing d relative to the unstrained spacing d_0, d/d_0.[13,15]'

We do however acknowledge that the definition of the term *T,* which represents the matrix transpose*,* was not obvious in our original manuscript, and we thank reviewer three for identifying this. We have updated the relevant discussion, to rectify this:

'The first term is the same trace formula as in nematic LCEs where μ is the linear shear modulus, $\underline{\underline{l}}$ the step length tensor which is expressed in terms of the step length anisotropy, r, $\underline{\underline{l}}$ = Diag(1, 1, r), $\underline{\underline{\lambda}}$ is the deformation tensor and $\underline{\underline{\lambda}}^T$ is the transpose of $\underline{\underline{\lambda}}$[13]. The second term relates to the smectic layer spacing, with layer spacing modulus $B = b/\mu$, and the initial director is defined along the z-axis, i.e. $n_0 = (0,0,1)^T$. The expression $1/\left|\underline{\underline{\lambda}}^{-T} \cdot n_0\right|$ represents the smectic layer spacing d relative to the unstrained spacing d_0, d/d_0.[13,15]'

8) Lines 214-218: keep the font consistent with other parts

We thank reviewer three for highlighting this formatting error, which has now been corrected

9) Line 236: How do we know the layer spacing modulus? from the slope of the plot?

Estimates for the layer spacing modulus were performed by fitting second order polynomials to tensile strain data, and have been previously reported (DOI: 10.1039/D5TC01484K), based on methods deduced by Warner and Adams in 2005 (DOI: 10.1103/PhysRevE.71.021708). We understand that in our original manuscript this could have been made clearer, and thus have acknowledged the previous work more clearly, copied below for the reviewer's convenience:

'Minimising such an equation requires numerical methods and the solution when r=2.1 and b=7.5 (solid), 3.4 (dashed), and 5.7 (dotted) are shown in Figure 4 (b), where values of b have previously been determined experimentally[12].'

10) Lines 377, 380: Stress-strain?

The term 'strain-strain' used on line 377 in the original manuscript (now line 524 in the revised version) and thereafter is correct in the context of these experiments. The relevant section of the manuscript is detailing the measurement of macroscopic changes in samples dimension upon an applied strain (to yield the data plotted in Figure 1). Thus, we are measuring the effect of an applied strain, on the induced strain in the transverse axes, hence the strain-strain nature of the data. We have renamed this subsection 'Macroscopic shape change and measurements of Poisson's ratio' in

an attempt to eliminate future readers from making the same assumption that ‘stress-strain’ was the intended phrasing.

Dr Stuart R. Berrow
School of Physics and Astronomy
University of Leeds
Leeds, LS2 9JT
S.R.Berrow@leeds.ac.uk
Wednesday 22nd July 2026

Dear referees,

Firstly, we would like to thank you once again for your time in considering, our manuscript titled 'Frustrated Smectic Liquid Crystal Elastomers as Multifunctional Mechanical Metamaterials' submitted for consideration in *Nature Communications* for the Nature portfolio collections on 'Advances in Liquid Crystal Elastomers'.

Thank you all for your positive appraisals of our work. We are delighted that you agree that our manuscript can be accepted for publication in nature communications.

Sincerely, on behalf of myself and my co-authors,

Dr Stuart Ryan Berrow
Research Fellow, University of Leeds

Reviewer One

I have reviewed the revised manuscript and the authors' responses. All my previous concerns have been satisfactorily addressed. The manuscript now presents a clear and convincing study of auxetic behaviour in smectic liquid crystal elastomers and will be of broad interest. I have no further comments and recommend publication in Nature Communications.

We thank reviewer one for their positive appraisal of our work.

Reviewer Two

The authors have well addressed all my concerns. I think its current version can be accepted.
We thank reviewer two for their positive appraisal of our work.

Reviewer Three

The authors addressed the comments/suggestions/questions by the reviewer.
I recommend the publication of the manuscript as it is.
Congratulations!

We thank reviewer three for their positive appraisal of our work.
